# Active Transport of Macrocycles into Micelles Using Molecular Pumps

**DOI:** 10.1002/anie.202512899

**Published:** 2025-09-23

**Authors:** James S. W. Seale, Swagat Sharma, Christopher K. Lee, Han Han, Tyler Jaynes, Eric W. Roth, Saman Shafie, Yunyan Qiu, Luke Malaisrie, Madison I. Bardot, Long Zhang, Yi‐Kang Xing, Dong Jun Kim, Samuel I. Stupp, R. Dean Astumian, Evan A. Scott, William R. Dichtel, J. Fraser Stoddart

**Affiliations:** ^1^ Department of Chemistry Northwestern University Evanston Illinois 60208 USA; ^2^ Department of Biomedical Engineering Northwestern University Evanston Illinois 60208 USA; ^3^ School of Chemistry University of New South Wales Sydney NSW 2052 Australia; ^4^ Department of Chemistry The University of Hong Kong Hong Kong SAR China; ^5^ Department of Chemistry National University of Singapore 3 Science Drive 3 Singapore Republic of Singapore 117543; ^6^ Department of Chemistry Department of Materials Science and Engineering Department of Medicine Center for Regenerative Nanomedicine Northwestern University Evanston Illinois 60208 USA; ^7^ Department of Physics and Astronomy University of Maine Orono ME USA; ^8^ NanoSTAR Institute, Department of Biomedical Engineering University of Virginia School of Medicine Charlottesville VA USA; ^9^ Stoddart Institute of Molecular Science Department of Chemistry Zhejiang University Hangzhou 310021 China; ^10^ ZJU‐Hangzhou Global Scientific and Technological Innovation Center Hangzhou 311215 China

**Keywords:** Active transport, Artificial molecular machines, Artificial molecular pumps, Micelles, Nonequilibrium processes

## Abstract

During the past decade, researchers have designed and synthesised a variety of artificial molecular pumps capable of the active transport of macrocycles (rings) from free solution into mechanically interlocked states. In their ability to drive non‐equilibrium transport, these artificial molecular pumps imitate natural transmembrane transporters, which are widespread in living organisms. Despite this resemblance, ring‐threading molecular pumps have not previously been operated in aqueous supramolecular assemblies in imitation of their natural counterparts. Here, we demonstrate the active transport of charged macrocycles from aqueous solution into micellar assemblies of polymer chains, which remain stable after pumping has occurred. While micelles are used routinely to encapsulate and solubilise hydrophobic small molecules in aqueous solution, this report, by contrast, shows that artificial molecular pumps can harness external energy to drive hydrophilic molecules into micelles where they are stored at concentrations far from equilibrium.

## Graphical abstract

Artificial molecular pumps attached to polymeric micelles were found capable of transporting charged macrocycles into the micelles. The pumps operate with only modestly reduced pumping efficiency as compared to nonmicellar counterparts, indicating that ring‐threading molecular pumps can transport charged solutes into supramolecular assemblies, akin to nature's transmembrane transporters which actively transport solutes into and out of cells.

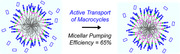

## Introduction

A variety of artificial chemical systems capable of the active transport of small molecules or ions have been reported in the scientific literature. Some have been shown to transport ions across nanoscale liposomal membranes^[^ ^1^, ^2^ ^]^ while others can transport molecules or ions through macroscale organic phases in U‐tube set‐ups.^[^ ^3^, ^4^, ^5^, ^6^ ^]^ Another class that has emerged in the last decade is artificial ring‐threading molecular pumps^[^ ^5^, ^7^, ^8^, ^9^, ^10^, ^11^, ^12^ ^]^ (AMPs). These are small molecules capable of the controlled capture and non‐equilibrium transport of macrocycles (rings). One marked feature of this class of molecular pumps is a mechanism of operation in which rings are forced into threaded states and are stored mechanically bonded to the collecting chain attached to the molecular pump after operation. The pumping ability of AMPs imitates that of the ubiquitous^[^ ^13^ ^]^ and diverse^[^ ^14^ ^]^ biological molecular machines that control the transport of molecules and ions into and out of cells. Since the first report of an AMP a decade ago,^[^ ^7^ ^]^ a variety of pumps^[^ ^8^, ^9^, ^10^, ^11^, ^15^, ^16^, ^17^ ^]^ capable of the active transport and storage of a range of rings have been reported. Although AMPs are mechanistically^[^ ^18^ ^]^ and functionally similar to natural membrane transporters, they are structurally very different. The mechanical interlocking of the pump with its small‐molecule cargo represents a mode of active transport apparently absent from natural systems. Further, AMPs—having masses on the order of 1 kDa as compared with the tens or hundreds of kDa typical in the case of transmembrane transporters—are much smaller than their natural counterparts and can be thought of as minimal molecular pumps. Indeed, the AMP employed in this investigation has a mass of 860 Da, while one isoform of halorhodopsin—one of nature's smaller membrane transporters—weighs in^[^ ^19^ ^]^ at 26,962 Da. In light of their novel, abiotic transport mechanism and their relatively simple designs, the range of functions for which AMPs can be adapted is an ongoing subject of investigation. AMPs have been shown to imbue the thermal processes of binding and release of macrocycles with directionality in a variety of contexts: whether acting as independent small molecules in free solution,^[^ ^7^, ^8^, ^9^, ^10^, ^11^, ^15^, ^16^, ^17^ ^]^ at the termini of polymeric chains,^[^ ^20^, ^21^ ^]^ or in dense arrays attached to solid surfaces.^[^ ^22^, ^23^ ^]^ In this paper, we report that AMPs can drive the active transport of hydrophilic molecules from aqueous solution into micellar supramolecular assemblies.

**Figure 1:**
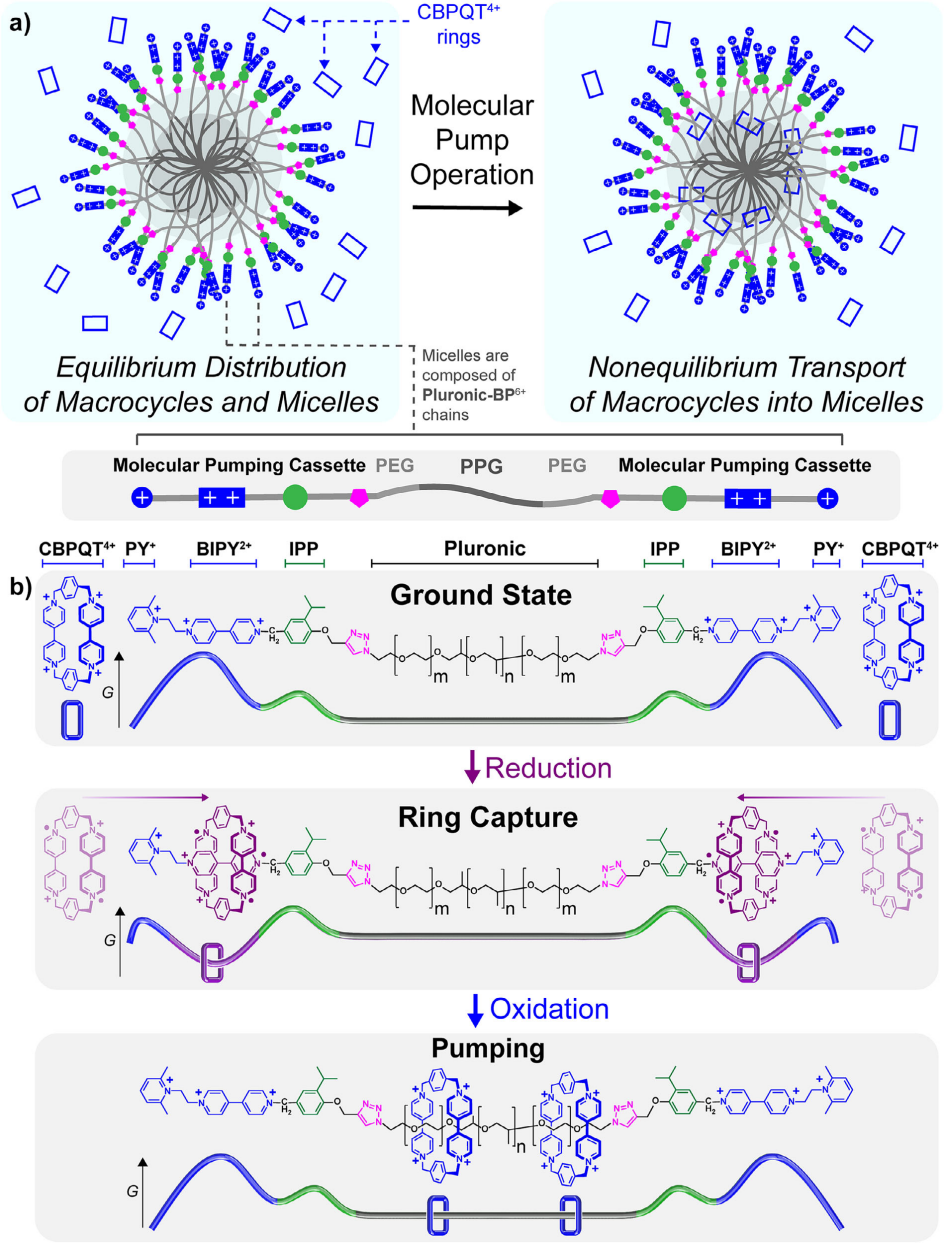
Mechanism of micelle‐bound molecular pump operation. a), Graphical representation of the active transport of **CBPQT** ^4+^ rings as their Cl^−^ salts into polymeric micelles composed of **Pluronic‐BP** ^6+^ chains as their TFA^−^ salts. Prior to molecular pump operation, an equilibrium distribution of **Pluronic‐BP** ^6+^ micelles and **CBPQT** ^4+^ rings exists in solution. After molecular pump operation, a non‐equilibrium distribution of species exists in which CBPQT^4+^ rings are stored inside the polymeric micelles. A cross‐section of the micelle corona and core are illustrated for the sake of clarity. b), Structural formula showing the key structural fragments and stages of operation of the molecular pump. The cyclobis(paraquat‐*p*‐phenylene) rings, the 2,6‐dimethypyridinium units, the bipyridinium units, the isopropylphenylene units, and the triblock copolymer chains are labelled as CBPQT^4+^/ PY^+^/ BIPY^2+^/ IPP / Pluronic, respectively. A qualitative energy landscape representing the energy of transit of threaded CBPQT^4+^ rings along the **Pluronic‐BP^6+^** chains is shown beneath each structural formula. Upon reduction, the **CBPQT^2(+•)^** rings in the bulk solution form trisradical tricationic inclusion complexes with the BIPY^+•^ units in the pumping cassettes. Upon oxidation of the system back to its ground state, the captured CBPQT^4+^ rings are forced to exit the BIPY^2+^ sites on account of strong Coulombic repulsion. Because of the additional Coulombic repulsion provided by the terminal PY^+^ units, the released CBPQT^4+^ rings travel with unidirectionality towards the central Pluronic chains where they are trapped in metastable threaded states. The difference in the relative barrier heights between the oxidised and reduced states confers the necessary kinetic asymmetry to allow for pumping. An idealised 100% efficient pumping cycle is shown in which a ring is captured by each pump and transported onto the collecting chain.

The AMPs employed in this investigation (Figure 1) operate as redox‐driven energy ratchets to promote the unidirectional transport of cyclobis(paraquat‐*p*‐phenylene)^[^ ^24^ ^]^ (**CBPQT^4+^**) rings onto collecting chains. The pumping cassette comprises a disubstituted bipyridinium (BIPY^2+^) recognition site linked 1) by a bismethylene bridge to a 2,6‐dimethylpyridinium (PY^+^) cationic terminal unit and 2) by a methylene group to an isopropylphenylene (IPP) steric barrier. This molecular pumping cassette was first reported^[^ ^9^ ^]^ in 2017 and its mode of operation has been described extensively in subsequent publications.^[^ ^15^, ^16^, ^17^, ^20^, ^25^ ^]^ When the bipyridinium units in both the pumps and rings are singly reduced, the formation of trisradical tricationic host–guest complexes between the pumps and rings are favoured (Figure 1b). Previous research has shown that a small excess of electrons^[^ ^26^ ^]^ is necessary to catalyse the formation of this host–guest complex. Upon oxidation, the possibility of radical–radical bonding interactions is eliminated and, driven by strong Coulombic repulsion, the rings depart the BIPY^2+^ recognition sites on the pumping cassettes, exiting preferentially by passage over the IPP steric barriers. The pumped rings are thus kinetically trapped in a mechanically interlocked metastable state on the collecting chains. Previous research works have employed the molecular pumps and **CBPQT^4+^** rings primarily with hexafluorophosphate counterions in acetonitrile solution. Here, we employ hydrophilic counterions (namely chloride ions and trifluoroacetate ions), which render the molecular pumps and macrocycles soluble in aqueous media and allow the aqueous‐phase pumping of macrocycles onto collecting chains.^[^ ^27^ ^]^

Recent investigations established^[^ ^20^, ^21^ ^]^ that these redox‐driven AMPs can pump rings efficiently onto poly(ethylene glycol) (PEG) and poly(propylene glycol) (PPG) chains and that the resulting non‐equilibrium polyrotaxanes are stable. We exploited the known ability to pump rings onto polyether chains in the construction of micelle‐forming block copolymers end‐capped with pumping cassettes. Pluronics,^[^ ^28^, ^29^ ^]^ or poloxamers, are synthetic triblock copolymers of the form PEG–PPG–PEG that undergo supramolecular assembly in aqueous solution. Micelle formation by Pluronics has been described extensively in the scientific literature^[^ ^28^, ^29^, ^30^, ^31^, ^32^ ^]^ for over half a century.

## Results and Discussion

### Synthesis

**Figure 2:**
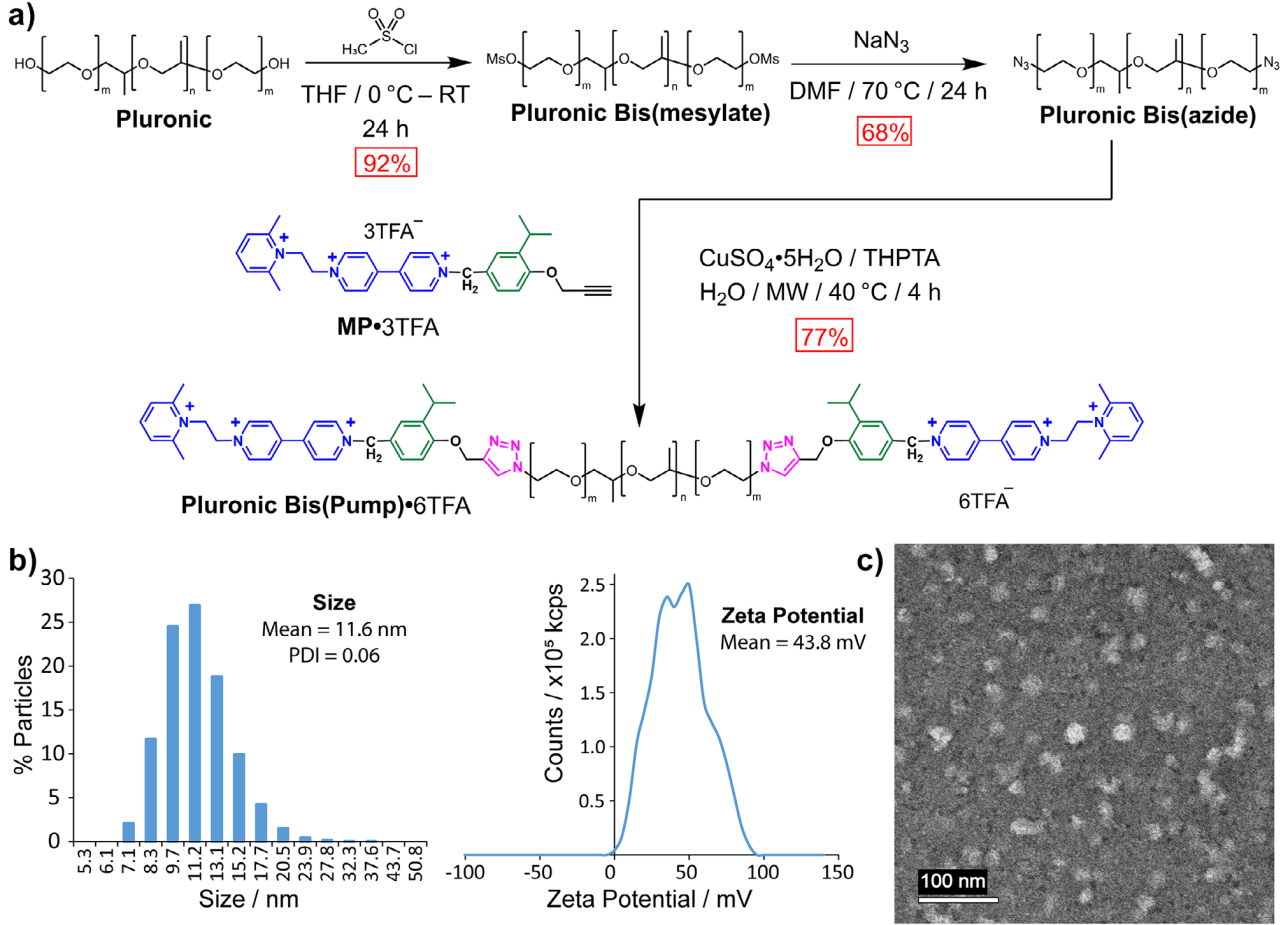
Synthesis and micellisation of Pluronic‐BP^6+^. a), Synthesis of Pluronic‐bis(pump)^6+^ (**Pluronic‐BP** ^6+^) as its TFA^−^ salt, beginning from a commercial sample of Pluronic P123 (**Pluronic**). Attachment of methanesulfonyl groups to the termini of **Pluronic** by use of an S_N_2 reaction produces **Pluronic bis(mesylate)**, which can in turn be reacted with sodium azide by use of another S_N_2 reaction to produce **Pluronic bis(azide)**. The molecular pump, **MP** ^3+^, as its TFA^−^ salt, is attached to the chain termini by use of copper‐catalysed azide–alkyne cycloadditions. Fractionation of **Pluronic‐BP** ^6+^ during reversed‐phase column chromatography allows chains with the desired average segment lengths of PEG and PPG components (denoted m and n, respectively) to be grouped for use in micellar experiments. b), DLS and zeta potential measurements of a **Pre‐Pump** solution of **Pluronic‐BP**•6TFA and **CBPQT**•4Cl in D_2_O. The results indicate the presence of micelles of average size 11.6 nm and average zeta potential + 43.8 mV. The positive zeta potential of the micelles can be attributed to the charged molecular pumping cassettes at the termini of **Pluronic‐BP** ^6+^. c), TEM image of the **Pre‐Pump** micellar solution showing the presence of spherical micelles with diameters in the range of 10–15 nm, consistent with size results obtained from DLS measurements.

The **Pluronic** starting material used in this investigation is composed primarily of a large triblock copolymer of average composition (PEG)_25_(PPG)_83_(PEG)_25_, *M*_n_ ≈ 7085. See Supporting Information. **Pluronic** was reacted (Figure 2) with methanesulfonyl chloride to produce (92% yield) **Pluronic bis(mesylate)**, which reacted subsequently with sodium azide to affording **Pluronic bis(azide)** in 68% yield. The molecular pump, **MP**•3TFA (where TFA^−^ is the trifluoroacetate anion), the synthesis of which is described elsewhere,^[^ ^9^ ^]^ was attached to the termini of **Pluronic bis(azide)** by a copper‐catalysed azide–alkyne cycloaddition. The crude reaction mixture was subjected to reversed‐phase column chromatography to obtain the end‐capped polymer, **Pluronic bis(Pump)**•6TFA (**Pluronic‐BP**•6TFA), in an estimated 77% yield. Fourier‐transform infrared (FT‐IR) spectroscopy (Figure S1), ^1^H and diffusion‐ordered (DOSY) NMR spectroscopies (Figures S5 and S8), and size‐exclusion chromatography (SEC) (Figure S29) confirmed the isolation of **Pluronic‐BP**•6TFA and its polymeric composition. Trifluoroacetate was employed as the counterion for synthetic convenience.

### Micelle Formation and Pumping Experiments

**Pluronic‐BP**•6TFA (average composition MP‐(PEG)_24_(PPG)_90_(PEG)_25_‐MP, *M*_n_ = 9089) was found to form spherical micelles in deuterium oxide (D_2_O) solution, including in the presence of **CBPQT**•4Cl. Dynamic light scattering (DLS) measurements (Figure 2b) show an average particle size of 11.6 nm with a polydispersity index (PDI) of 0.06. Transmission electron microscopy (TEM) (Figure 2c) showed that the particles are spherical micelles with diameters in the range of 10–15 nm. Cryogenic TEM (Cryo‐TEM) images (Figure S46) also confirmed the presence of spherical micelles. The concentration of **Pluronic‐BP**•6TFA (5.0 mg mL^−1^, 0.55 mM) used for the pumping experiments was chosen because micelles were present and there was sufficient resolution of all solution components by NMR spectroscopy. The concentration of **CBPQT**•4Cl (1.5 mg mL^−1^, 2.26 mM) used in the solutions was that found to be optimal for efficient pumping in aqueous solution (See Supporting Information Section 4). Solutions at these concentrations will henceforth be termed the **Pre‐Pump** solution.

The **Pre‐Pump** solution was prepared by dissolving **Pluronic‐BP**•6TFA and **CBPQT**•4Cl in D_2_O that had been sparged with nitrogen for 40 min to minimise^[^ ^33^ ^]^ the concentration of dissolved oxygen, which might otherwise limit efficient reduction of the species in solution. D_2_O was used as solvent throughout this investigation to allow ^1^H NMR spectroscopic analysis of the micellar solutions. The resulting micellar solution was filtered through a hydrophilic 100 µM syringe filter to remove any dust or other extraneous particles before transferring to a nitrogen‐filled purge box. When an excess of zinc dust, which is known^[^ ^9^ ^]^ to be a suitable reducing agent for the operation of the pump, was added with gentle shaking the solution rapidly turned an opaque purple colour. The reduced purple solution was left to stand for 30 min after which it was centrifuged (1000 RPM, 30 s) and decanted into a round‐bottomed flask outside the purge box to separate out the zinc dust. The solution was sparged with air that acted as the oxidant and after 5–10 min, the deep purple colour of the solution had faded to a pale blue–purple colour. The micellar solution formed after the operation of the pumps will henceforth be called the **Post‐Pump** solution. Note that, after bubbling with air, the **Post‐Pump** solution is in an aerated state, and efficient reduction of the solution using zinc powder to initiate another pumping cycle cannot be achieved. In order to perform multiple pumping cycles on this system, it would be necessary to first evaporate the aerated solvent, then redissolve the precipitate in N_2_‐sparged D_2_O.

**Figure 3:**
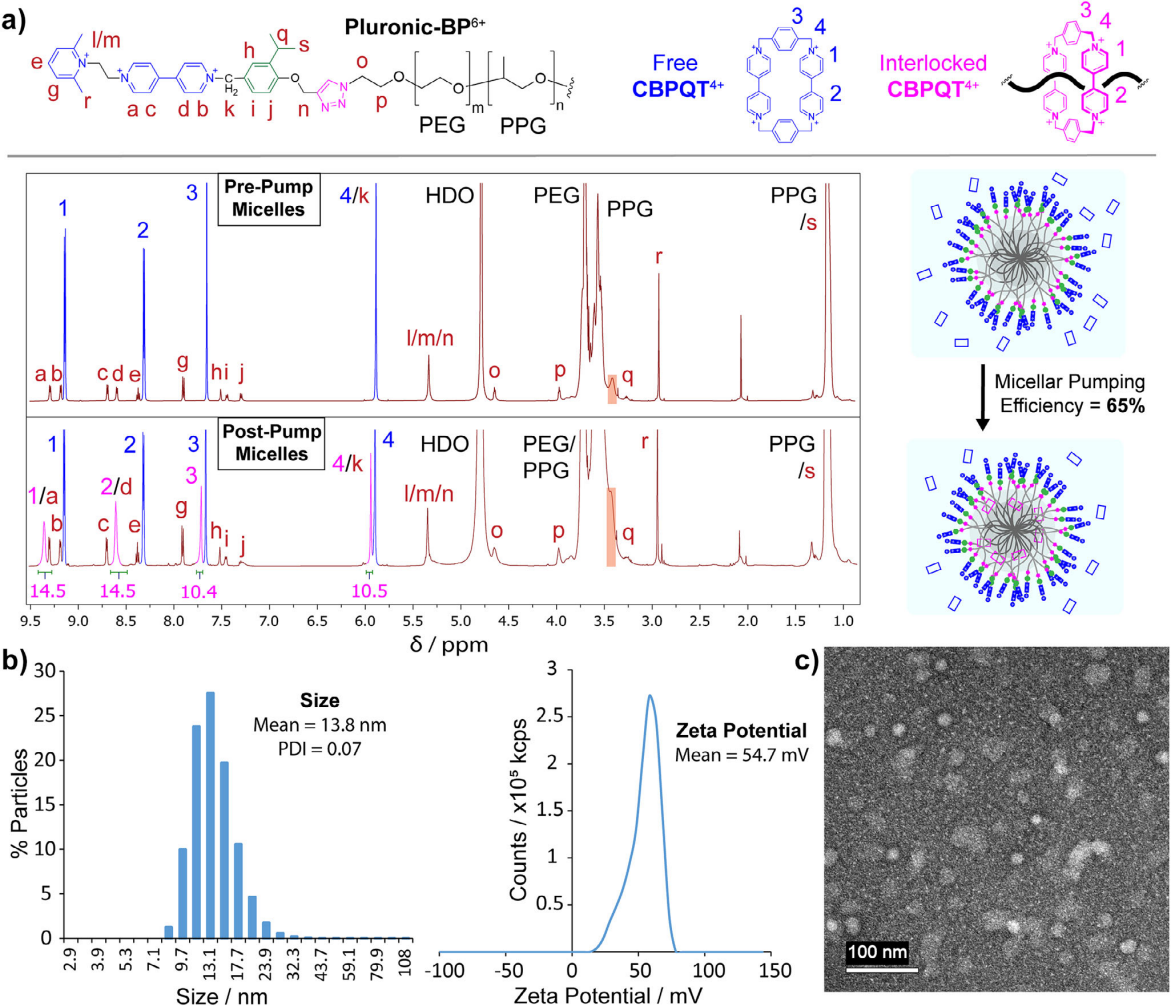
Transport of CBPQT^4+^ rings into micelles of Pluronic‐BP^6+^. a), Annotated ^1^H NMR spectra (600 MHz, D_2_O, 298 K) of the **Pre‐Pump** micellar solution (top) and the **Post‐Pump** micellar solution (bottom) produced by reduction with zinc powder followed by oxidation by sparging air. The resonances corresponding to **CBPQT** ^4+^ free in solution and CBPQT^4+^ stored in polymeric micelles are coloured blue and magenta, respectively. The resonance peak at 3.4 ppm, which indicates the presence of pluronic micelles, has been highlighted in orange. A graphical representation of the polymeric micelles is shown adjacent to the respective ^1^H NMR spectrum. b), DLS and zeta potential measurements for the **Post‐Pump** solution represented by the bottom ^1^H NMR spectrum in (a). The results indicate the presence of micelles of average size 13.8 nm and average zeta potential + 54.7 mV. The higher zeta potential of the **Post‐Pump** solution, compared with the **Pre‐Pump** solution (Figure 2b), can be attributed to the transport of CBPQT^4+^ rings into the micelles. c), TEM image of the **Post‐Pump** micellar solution. The image shows the presence of spherical micelles with diameters in the range of 10–20 nm, consistent with sizes measured by DLS, indicating that the micellar morphology of the **Pre‐Pump** solution (Figure 2c) is largely retained in the **Post‐Pump** solution.

^1^H NMR spectroscopy indicates that mechanically interlocked CBPQT^4+^ rings are present in the **Post‐Pump** solution. Previous investigations^[^ ^20^, ^21^ ^]^ have demonstrated the accuracy of ^1^H NMR spectroscopy for the determination of the number of rings mechanically interlocked on polyether chains following pumping. This quantification is performed by integration of the distinct resonances corresponding to the mechanically interlocked CBPQT^4+^ rings that are observed (Figure 3a) in the ^1^H NMR spectra. The synthetic pumping efficiency—defined as the number of mechanically interlocked CBPQT^4+^ rings divided by the number of pumping cassettes—was measured at 65% for the air‐oxidised **Post‐Pump** solution. The resonance peaks corresponding to protons on mechanically interlocked rings are substantially broader than those corresponding to free rings. For example, the full‐width half‐max of peak 3, corresponding to the phenylene protons, is 1.3 Hz for free macrocycle and 7.2 Hz for pumped macrocycles. This broadening is in line with previous observations^[^ ^20^, ^21^ ^]^ for ^1^H NMR spectroscopy of pumped **CBPQT^4+^** rings and may also be indicative of a shortened relaxation time on account of the transfer of those rings to the micellar phase.

The TEM, DLS, and zeta potential results indicate that micelles are still present in the **Post‐Pump** solution but there was a modest increase in both the size (from 11.6 to 13.8 nm) and zeta potential (from + 43.8 to + 54.7 mV) of the micelles. The increases in micelle size and zeta potential can be attributed to the transport of positively charged **CBPQT^4+^** rings into the micelles. Various ^1^H NMR spectroscopic techniques provide further evidence of the presence of micelles in the **Pre‐** and **Post‐Pump** solutions. It is well documented^[^ ^34^, ^35^ ^]^ that a distinct resonance peak (3.4 ppm) appears in the ^1^H NMR spectrum of micellar Pluronic and is not observed in solutions of unimeric Pluronic. This anhydrous core resonance, as it will here be termed, can be attributed^[^ ^36^ ^]^ to ─CH_2_— protons of anhydrous segments of PPG chains buried in the micellar core. This resonance is observed in both the **Pre‐Pump** and **Post‐Pump** solutions (Figure 3a). We found that the anhydrous core resonance registers an average diffusion constant of 3.21 x 10^−7^ cm^2^ s^−1^ in the **Pre‐Pump** solution (Table S2) as measured by DOSY NMR spectroscopy. When this diffusion constant was inserted into the Stokes–Einstein equation (see Supporting Information Section 5), a hydrodynamic diameter of 12.4 nm was calculated, which is close to the diameter of 11.6 nm measured by DLS. The spin–spin relaxation times (*T*_2_ values) of resonances in the ^1^H NMR spectra of the **Pre‐Pump** micellar solution were measured (see Table S3). In accordance with previous reports,^[^ ^37^, ^38^ ^]^ the *T*_2_ value measured for the core PPG resonances was lower than that for the PEG resonances (56 ms compared with 367 ms, respectively). These results confirm that core PPG units exist in a densely packed microenvironment (the micelle core), while PEG units exist in a less‐dense microenvironment (the micelle corona) in which higher chain mobility is implicit.^[^ ^38^ ^]^ We further note that DLS measurements of the **Pre‐Pump** micellar solution after reduction (Figure S35) indicate that the system remains in an aggregated state during the reduction stage of molecular pump operation.

**Figure 4:**
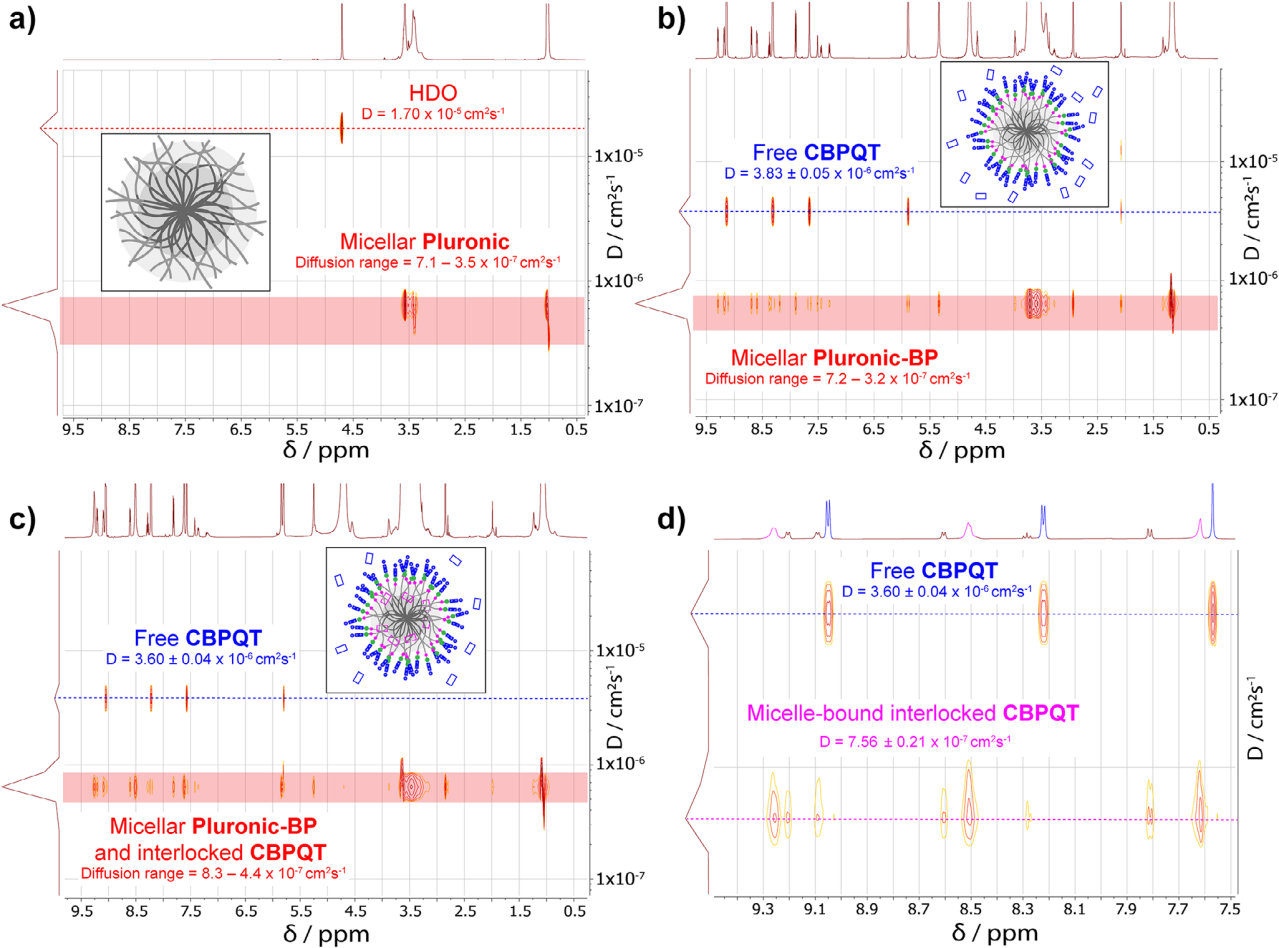
^1^H DOSY NMR spectra showing the transport of CBPQT^4+^ **rings into micelles**. A graphical representation of each micellar solution is displayed on each graph. a), ^1^H DOSY NMR Spectrum (600 MHz, D_2_O, 298 K) of a solution of micellar **Pluronic**. b), ^1^H DOSY NMR Spectrum (600 MHz, D_2_O, 298 K) of a **Pre‐Pump** micellar solution of **Pluronic‐BP**•6TFA and **CBPQT**•4Cl. c), ^1^H DOSY NMR Spectrum (600 MHz, D_2_O, 298 K) of a **Post‐Pump** micellar solution. d), ^1^H DOSY NMR Spectrum (600 MHz, D_2_O, 298 K) of the **Post‐Pump** micellar solution shown in **c** magnified in the aromatic region. Cross‐peaks corresponding to resonances of free **CBPQT** ^4+^ (blue) and interlocked CBPQT^4+^ diffusing at the micellar diffusion rate (magenta) can be distinguished. All DOSY NMR spectra except for that shown in (a) were collected with suppression of the HDO peak in order to improve resolution.

The ^1^H DOSY NMR spectroscopy results (Figure 4) confirm that **CBPQT** ^4+^ rings are transported into micelles by the action of the molecular pumps. The diffusion constant of pumped CBPQT^4+^ rings in the **Post‐Pump** solution is, at 7.56 ± 0.21x 10^−7^ cm^2^ s^−1^, five times lower than that of free **CBPQT** ^4+^ in the **Pre‐Pump** solution (38.3 ± 0.50 10^−7^ cm^2^ s^−1^). The diffusion constant of pumped CBPQT^4+^ is between the average diffusion constants measured for the PEG and PPG segments of the polymer chains that compose the micelles, strongly suggesting that the mechanically interlocked CBPQT^4+^ rings are components of the micelles.

### Thermodynamic Analysis of Pumping

Calculation of the chemical potential difference (Δ*μ*) between rings pumped into the micelles and rings in free solution allows quantification of the energy stored by the molecular pumps. The chemical potential difference is given by,

(1) $$\Delta \mu =RTln\left(\frac{{\left[r_{sol}\right]}_{eq}}{{\left[r_{mic}\right]}_{eq}}\frac{{\left[r_{mic}\right]}_{ps}}{{\left[r_{sol}\right]}_{ps}}\right)$$

where *R* is the gas constant, *T* is the temperature (approximately 298.15 K for all pumping experiments), and [*r*] is the concentration of rings under given conditions. The subscripts *sol* and *mic* refer to rings in free solution and rings in micelles respectively, and the subscripts *eq* and *ps* refer to the equilibrium (**Pre‐Pump**) state and the pumped state (**Post‐Pump**), respectively. The concentration [*r_sol_*]_*eq*_ is known from experiment, the concentrations [*r_mic_*]_*ps*_ and [*r_sol_*]_*ps*_ were calculated from NMR spectroscopic data (specifically, from the known concentrations of rings and chains prepared in the **Pre‐Pump** solution and from the measured pumping efficiency) and the upper‐limit of the concentration [*r_mic_*]_*eq*_ was calculated based on the detection limit of our NMR spectroscopy method (for a full discussion of how these values were calculated, see Supporting Information Section 11). Inserting these values we find the minimum change in chemical potential of rings pumped into micelles to be Δμ > 17.3 kJ mol^−1^.

### Small‐Molecule Pumping

**Figure 5:**
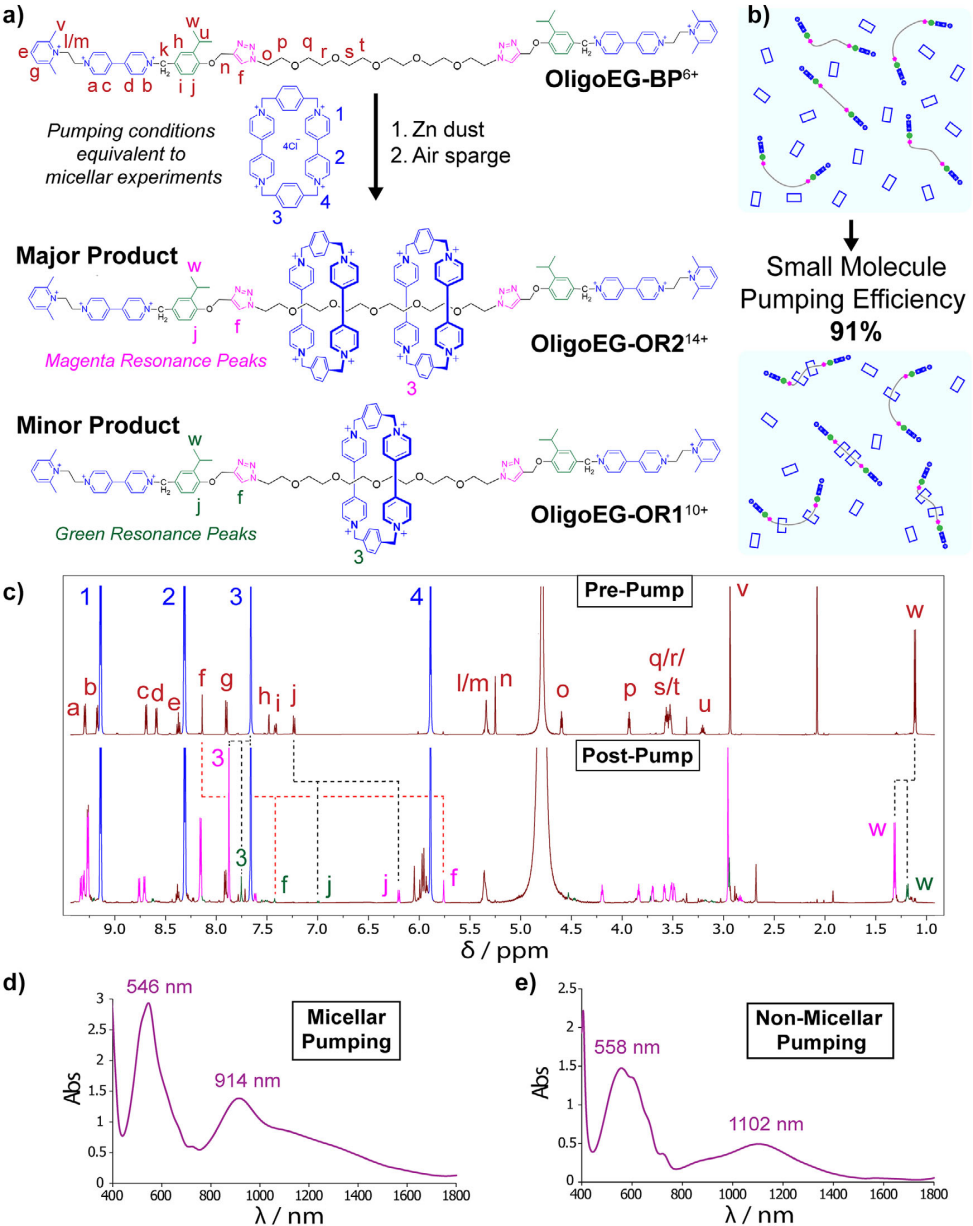
A small‐molecule pumping control experiment a), A synthetic scheme representing the small‐molecule pumping experiment. The reactions were performed under conditions equivalent to those of the micellar pumping experiment, featuring the same solvent (D_2_O), the same concentrations of bis‐pump molecules (0.55 mM) and of rings (1.5 mg mL^−1^, 2.26 mM), and following the same pumping procedure using zinc dust as the reductant and air sparging for oxidation. b), Graphical representation of the small‐molecule pumping experiment. c), Annotated ^1^H NMR spectra of the small‐molecule control pumping experiment before (top) and after (bottom) pump operation. The annotations refer to proton resonances labelled in (a). The shifts of peaks 3/f/j/w are highlighted because the equivalent peaks in both **OligoEG‐OR1** (resonances coloured green) and **OligoEG‐OR2** (resonances coloured magenta) occur with minimal overlap, and hence were the peaks whose integrals were used to calculate the relative concentration of each species. The control experiment results in an almost 5:1 ratio of **OligoEG‐OR2**:**OligoEG‐OR1**, from which a pumping efficiency of 91% is calculated. d) & e), UV–vis–NIR Spectra recorded for the reduced states of the micellar (d) and the nonmicellar (e) pumping experiments. The nonmicellar experiment shows a higher relative absorbance at 1102 nm, a wavelength associated with the trisradical tricationic complex. There is little/no absorbance at ca. 900 nm, a wavelength region associated with bipyridinium pimerisation. The lower pumping efficiency of micelle‐bound pumps therefore arises in tandem with the appearance of bipyridinium–bipyridinium interactions that may compete with or limit efficient pumping.

In order to examine the effect of micellar assembly on molecular pump efficiency, a small‐molecule pumping control experiment was performed (Figure 5) using a hexa(ethylene glycol) oligomer with pumping cassettes covalently attached to each end (**OligoEG‐BP•**6TFA). This small molecule was found to not form aggregates in D_2_O solution. A pumping experiment equivalent to that performed on micellar **Pluronic‐BP•**6TFA was carried out (Figure 5a) on a solution of unimeric **OligoEG‐BP•**6TFA. A synthetic pumping efficiency of 91% (Figure 5b), based on the quantities of the [2] and [3]rotaxanes formed (**OligoEG‐OR1** ^10+^ and **OligoEG‐OR2** ^14+^, respectively), was found as measured by ^1^H NMR spectroscopy. A portion of the reaction constituents was found to precipitate out of solution during the reduction stage of the pumping process, but once redissolved after oxidation, this precipitate showed the same relative proportion of rotaxanes as the solubilised portion.

Given the energetic demands of transporting charged, hydrophilic macrocycles into the confined spaces of hydrophobic micelles, one might imagine that micellar pumping would be unlikely to occur with good synthetic pumping efficiency. Our results show, however, that the decrease of pumping efficiency in micelles relative to that of molecularly dispersed pumps is modest, from 91% to 65%. The UV–vis–NIR spectrophotometry spectra (Figure 5d,e) of the reduced micellar and nonmicellar solutions allow insight into the interactions occurring during the reduction phase of pumping. The spectrum for the micellar solution shows the appearance of interactions other than formation of the trisradical tricationic threaded complex necessary for pumping. The trisradical tricationic complexes formed between CBPQT^2(+•)^ rings and BIPY**^+•^** units are known to show^[^ ^26^, ^39^, ^40^ ^]^ a characteristic broad absorption at ca. 1080 nm in UV–vis spectra. This absorption is observed at ca. 1100 nm for both the reduced micellar and reduced nonmicellar solutions. In the micellar experiment, however, the absorption at ca. 1100 nm is dwarfed by an absorption peak at 914 nm that is associated with generic viologen dimerisation. Since this prominent peak at 914 nm is only observed in the reduced micellar solution, the interactions that are the source of the peak must arise as a result of the presence of the micelles. Indeed, the micellar structure, which arranges molecular pumping cassettes in close proximity to each other, is preorganised for intermolecular BIPY**^+•^**–BIPY**^+•^** interactions between adjacent pumping cassettes. We posit that these unproductive BIPY**^+•^**–BIPY**^+•^** interactions are associated with the modest decrease in pumping efficiency in the micellar solutions as compared to the small‐molecule scenario, likely by competing with formation of the trisradical tricationic complex.

We note that the trisradical complex absorbance peak (ca. 1100 nm) in the micellar solution is extremely broad, with absorbance detected up to ca. 1700 nm, while the equivalent peak in the spectrum of the reduced nonmicellar experiment extends just beyond 1400 nm. In a previous publication,^[^ ^41^ ^]^ it has been noted that multimeric interactions between paired BIPY**^+•^** units result in a red‐shifting of the NIR *λ*_max_ absorbance peak. We suggest that the dense arrangement of molecular pumping cassettes on the micelles leads to a degree of multimerisation of the trisradical complexes in the reduced state, manifesting itself as a broadening of the ca. 1100 nm peak to higher wavelengths. We expect that the different lengths of the collecting chains in **OligoEG‐BP•**6TFA and **Pluronic‐BP•**6TFA have little impact on the pumping efficiency, and that the nonproductive interactions in the reduced states, evinced in UV–vis–NIR spectrophotometry, are the main drivers of the disparity in pumping efficiency.

## Conclusion

The investigation reported here demonstrates that ring‐threading molecular pumps are capable of the active transport of charged molecules into aqueous polymeric micelles. A synthetic pumping efficiency of 65% was observed in micellar experiments, which is modestly lower than the 91% pumping efficiency observed in nonmicellar control experiments. Previous research has found^[^ ^42^ ^]^ that molecular restriction or competitive interactions are a common limitation on the efficient operation of molecular machines in dense arrays. The reduced pumping efficiency in the micellar experiments may arise as a result of competitive intermolecular interactions which occur during the first stage of pump operation. Although polymeric micelles have long found use for their ability to encapsulate and solubilise *hydrophobic* small molecules, the present report proves that the active transport of *hydrophilic* small molecules into micelles can be achieved using ring‐threading artificial molecular pumps. These micelle‐bound artificial molecular pumps demonstrate the ability to drive aqueous supramolecular assemblies away from equilibrium in imitation of natural membrane‐bound molecular pumps.^[^ ^14^ ^]^

## Supporting Information

The authors have cited additional references within the Supporting Information.^[^ ^33^ ^]^

## Conflict of Interests

The authors declare no conflict of interest.

## Supporting information



Supporting Information

## Data Availability

The data that support the findings of this study are available in the Supporting Information of this article.
