# Supplementary material for: Active Transport of Macrocycles into Micelles Using Molecular Pumps
Source: Angew Chem Int Ed Engl. 2025 Sep 23;64(46):e202512899. doi: 10.1002/anie.202512899 (PMC12603975; doi:10.1002/anie.202512899)
Supplement: Supplementary file 1 — Supporting Information [file ANIE-64-e202512899-s001.pdf]

Supporting Information

Active transport of macrocycles into micelles using molecular pumps

James S. W. Seale, Swagat Sharma, Christopher K. Lee, Han Han, Tyler Jaynes, Eric W. Roth, Saman Shafie, Yunyan Qiu, Luke Malaisrie, Madison I. Bardot, Long Zhang, Yi-Kang Xing, Dong Jun Kim, Samuel I. Stupp, R. Dean Astumian, Evan A. Scott, William R. Dichtel*, J. Fraser Stoddart*.

*Correspondence to: wdichtel@northwestern.edu

Table of Contents

1. Abbreviations S2

2. Materials, General Methods, and Equipment S3

3. Synthetic Protocols S6

4. Optimisation of Pump Operation S16

5. NMR Spectroscopy S19

6. Size Exclusion Chromatography S46

7. Dynamic Light Scattering and Zeta Potential S48

8. UV-Vis-NIR Spectrophotometry S54

9. Mass Spectrometry S57

10. Transmission Electron Microscopy S59

11. Thermodynamic Analysis S64

12. References S67

1. Abbreviations

No.	Abbreviation	Full name/description
1	**CBPQT**	cyclobis(paraquat-*p*-phenylene)
2	COSY	homonuclear correlation spectroscopy
3	CH_2Cl_2	dichloromethane
4	DLS	dynamic light scattering
5	DMF	*N,N*-dimethylformamide
6	DOSY	diffusion-ordered spectroscopy
7	h	hour / hours
8	m/v	mass / volume ratio
9	**MP**	molecular pump
10	NOESY	nuclear Overhauser effect spectroscopy
11	**OligoEG-BP**	a hexaethylene glycol chain of six repeat units end-capped with **MP** (BP = bis-pump)
12	**OligoEG-OR1**	**OligoEG-BP** bearing one mechanically interlocked **CBPQT** ring
13	**OligoEG-OR2**	**OligoEG-BP** bearing two mechanically interlocked **CBPQT** rings
14	PEG	polyethylene glycol
15	**Pluronic**	a Pluronic P123 sample purchased from Sigma Aldrich
16	**Pluronic-BP**	pluronic polymer chains end-capped with **MP** (BP = bis-pump)
17	**Post-Pump**	a solution of **CBPQT** and **Pluronic-BP** after the operation of molecular pumps
18	PPG	polypropylene glycol
19	**Pre-Pump**	a solution of **CBPQT** and **Pluronic-BP** prior to the operation of molecular pumps
20	RT	room temperature
21	THF	tetrahydrofuran
22	THPTA	tris(3-hydroxypropyltriazolylmethyl)amine
23	UV-Vis-NIR	ultraviolet – visible - near infrared

2. Materials, General Methods, and Equipment

All compounds were purchased from commercial sources and used as received. Pluronic P123 (manufacturer listed average M_n ~5800) was purchased from Aldrich and used as received. All reactions and manipulations involving air- and water-sensitive compounds were carried out under a dry N_2 atmosphere in a glovebox or by standard Schlenk techniques. Solvents and chemicals used in extractions and column chromatography were used as received. The yields of the derivatizations of Pluronic were determined based on the manufacturer's listed approximate number-average molecular weight of 5800 g/mol. All pumping experiments were performed at 25 ± 2 °C in a N_2-purged glove box. All deuterium oxide (D_2O) solvent for pumping experiments was degassed with N_2 for 40 min prior to use based on a publication that shows this method to be effective for the removal of dissolved oxygen from water[1]. For spectrophotometry in the reduced state, a cuvette (path length = 2 mm) was septum-sealed and the septum wrapped tightly with parafilm in a glove box before transport to the instrument and immediate measurement. For dynamic light scattering and zeta potential measurements in the reduced state, a stoppered cell was filled with solution in the glove box and its stoppers were wrapped tightly with parafilm prior to transport to the instrument.

Column Chromatography. Both normal phase (BUCHI FlashPure EcoFlex Silica) and reversed-phase (Biotage® Sfär C18 D - Duo 100 Å 30 µm and RediSep Rf Gold® Reversed-Phase C18) column chromatography were carried out using CombiFlash® Automation Systems (Teledyne ISCO).

Nuclear Magnetic Resonance (NMR) Spectroscopy. Spectra were recorded on a Bruker NOE 600 MHz spectrometer with QCI-F cryoprobe (^{1}H sensitivity = 5000), with working frequencies of 600 (^{1}H NMR) MHz or on a Bruker AVANCE III 500 MHz spectrometer with a DCH cryoprobe with working frequencies of 500 (^{1}H NMR) MHz. Chemical shifts are reported in ppm relative to the signals corresponding to the residual non-deuterated solvents (CD_3SOCD_3: δ_H = 2.50 ppm; CD_3OD: δ_H = 3.31 ppm; D_2O: δ_H = 4.79 ppm). All NMR spectra were recorded with a sample temperature of 25.0 ºC. For the measurement of quantitative ^{1}H NMR spectra, the NMR spectrometer was set with the following parameters: the relaxation delay time (D1): 11 s (polymers) or 13s (oligomers); the number of scans (ns): 128. This D1 relaxation time of 11 s for micellar systems was chosen based on inversion recovery experiments performed on three systems in D_2O solvent: (1) micellar mixtures of **CBPQT**•4Cl and **Pluronic-BP**•6TFA, (2) micellar mixtures of

CBPQT•4Cl and **Pluronic-BP**•6TFA after pumping, in which the polymer chains are interlocked with **CBPQT**•4Cl rings, and (3) micelles of **Pluronic-BP**•6TFA in which the polymer chains are interlocked with **CBPQT**•4Cl rings and where no free, non-threaded **CBPQT**•4Cl rings are present. In all scenarios the longest recorded T_1 value was 2.18 s, corresponding to the terminal aromatic protons of the molecular pumps. The D1 relaxation time of 13 s, used for **OligoEG-BP** and its rotaxanes, was based on T_1 relaxation results indicating the longest measured T_1 value to be 2.48 s corresponding to the terminal aromatic protons of the molecular pumps in **OligoEG-OR2**. Special care was taken to determine the spectrometer parameters used for DOSY NMR such that the substantially different diffusion constants of free **CBPQT**•4Cl and the polymeric micelles of **Pluronic-BP**•6TFA could be extracted accurately from the same experiment. The parameters used were based on prior literature[2] and experimental determination. Water signal suppression was used for DOSY spectra as it was found to improve resolution of the analyte peaks. For the measurement of all DOSY NMR spectra, the spectrometer was set with the following parameters: the pulse sequence: Bruker pulse program ledbpgppr2s; the relaxation delay (D1): 11 s (polymeric micelles) or 13s (oligomers); the diffusion time (D20): 400 ms; the diffusion gradient pulse length (P30): 2.0 ms; the number of scans (ns): 8 – 16; the number of gradient steps: 16 steps with linear spacing (oligomers) or 32 steps with exponential spacing (polymeric micelles); the gradient range: 2–50% (oligomers) or 2–95% (polymeric micelles). All NMR spectra were processed in MestReNova version 14.2.3-29241. In order to enhance resolution, the FIDs in DOSY datasets were apodized using an exponential function of 5.0 Hz and zero-filled to a spectral size of 128 K points. Diffusion constants were manually calculated from the raw 1D DOSY spectra. Mestrenova's Bayesian DOSY transform function was used to plot 2D DOSY graphs. For the measurement of sample T_2 values, the spectrometer was set with the following parameters: the pulse sequence; cpmgpr (Carr-Purcell-Meiboom-Gill which includes presaturation solvent suppression); the relaxation delay (D1): 6 s; the number of scans (ns): 8; the number of dummy scans (ds): 16; the values for the variable counter list of T_2 delay: 4/8/16/32/64/128/256/512/1024/2048 ms.

Dynamic Light Scattering. The size distribution and zeta potential of micelles were analyzed using a Zetasizer Nano (Malvern Instruments) with a 4 mW He-Ne 633 nm laser. All measurements were performed with a sample temperature of 25 °C.

Size-Exclusion Chromatography (SEC). Data were collected with an organic gel permeation chromatography set-up using Astra Version 7 data acquisition software and processed using Astra

Version 7 for data analysis. For SEC performed on THF solutions: the instrument was configured with an Agilent 1260 Series HPLC module equipped with a UV detector and coupled with Wyatt Heleos II MALS and RI detectors. The SEC analysis was run on a double PolyPore GPC column from Varian (7.6 x 250 mm) at a flow rate of 1 mL/ min with THF as a mobile phase. For SEC performed with dimethylformamide (DMF) solutions: measurements were carried out on a set of Phenomenex Phenogel 5m, 1K-75K, 300 x 7.80 mm in series with a Phenomex Phenogel 5m, 10K-1000K, 300 x 7.80 mm columns with HPLC grade solvents as eluents: DMF with 0.05 M of LiBr at 60 °C. Detection consisted of a Hitachi UV-Vis Detector L-2420, a Wyatt Optilab T-rEX refractive index detector operating at 658 nm and a Wyatt DAWN® HELEOS® II light scattering detector operating at 659 nm.

Matrix-Assisted Laser Desorption/Ionization (MALDI) Mass Spectrometry. Dithranol (16 mg), **Pluronic** (2 mg) and sodium trifluoroacetate (0.1 mg) were dissolved in DMF, spotted onto a MALDI plate and dried thoroughly – a method[3] reported previously in the literature**.** Mass spectrum data were collected on Bruker Rapiflex MALDI-TOF, using FlexControl data acquisition software and processed using SeeMS software version 3.0.23264.0 (9/21/2023) © 2023 Vanderbilt University. Reflectron positive mode was used for low-molecular-weight components of **Pluronic** and Linear positive mode for higher-molecular-weight components.

Electrospray Ionization Mass Spectrometry (ESI-MS). High-resolution mass spectra were recorded on an Agilent 6210 Time-of-Flight (TOF) LC-MS coupled with an electrospray ionization (ESI) source using 0.1 mg/mL solutions of sample in acetonitrile (for samples with PF_6 salts) or methanol (for samples with TFA salts).

Fourier-Transform Infrared Spectroscopy (FT-IR). Data was collected with an attenuated total reflection module on a Nexus 870 spectrometer from Thermo Nicolet.

Ultraviolet-visible-near infrared (UV-Vis-NIR) Spectroscopy. Spectra were measured using an Agilent Cary 5000 UV-Vis-NIR Spectrophotometer. The instrument operates by a double beam photometric system and samples were measured against a blank D_2O reference. Spectra were collected at a scan rate of 600 nm/min.

Transmission Electron Microscopy (TEM). Images were acquired of micelles in the dry state using a JEOL 1400 TEM operating at an acceleration voltage of 120 kV. Negative-stained TEM samples were prepared by drop-casting 8 μL of a solution on the carbon side of a plasma cleaned TEM grid (CF300-Cu, Electron Microscopy Science) followed by wicking with filter paper after one minute. Subsequently, a 8 μL droplet of a 1 wt% uranyl acetate solution was applied, followed by another round of wicking with filter paper after one minute, then dried for one hour before imaging.

Cryogenic Transmission Electron Microscopy (Cryo-TEM). 200-mesh lacey carbon grids were glow-discharged for 30 seconds in a Pelco easiGlow glow-discharger at 15mA with a chamber pressure of 0.24 mBar. Exposing the grids' carbon membrane to the atmosphere plasma helps aqueous solutions to spread out evenly on the otherwise hydrophobic surface of a TEM grid's carbon membrane. 4uL of sample was the pipetted onto a grid, blotted for 5 seconds, and plunge-frozen into liquid ethane using an FEI Vitrobot Mark IV cryo plunge freezing robot. Grids were then loaded into a Gatan 626.5 cryo transfer holder and imaged at -180C in a JEOL 1400 Flash TEM LaB6 emission TEM at 120kV. Data was collected using Gatan Digital Micrograph software connected to a Gatan OneView 4k camera and Gatan Digital Micrograph software.

3. Synthetic Protocols

Synthesis of MP•3TFA and CBPQT•4Cl

The molecular pump[4], **MP**, and the cyclophane[5], **CBPQT**, were prepared according to published procedures. In this study, **MP**•3TFA (where TFA refers to the trifluoroacetate counterion) was isolated directly from column chromatography fractions by drying the fractions without any counterion exchange, yielding **MP**•3TFA as a soft yellow solid. **CBPQT**•4Cl was obtained by adding a saturated solution of tetrabutylammonium chloride in acetonitrile to a concentrated solution of **CBPQT**•4PF_6 in acetonitrile. The white precipitate formed upon combination of these solutions was washed three times with acetonitrile by ultrasonication, dried by filtration, then dried under sealed vacuum yielding **CBPQT**•4Cl as a white powder.

Scheme S1 | Synthesis of Pluronic bis(mesylate)

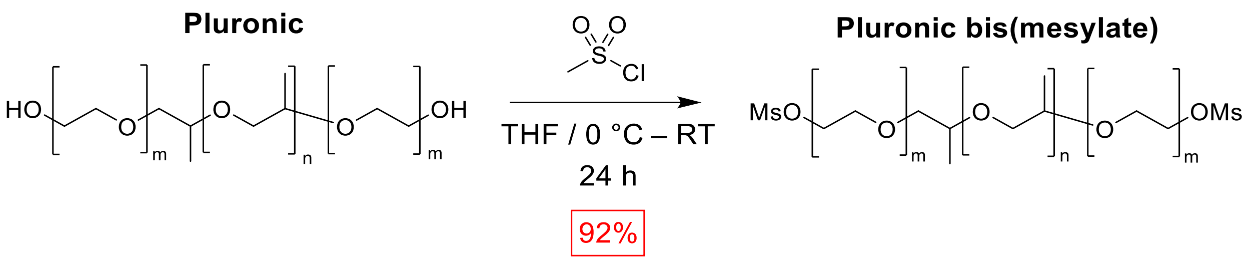

Pluronic bis(mesylate): **Pluronic** (5.226 g, 901 μmol), anhydrous Et_3N (505 mg, 4.99 mmol), and inhibitor-free anhydrous THF (25 ml) were combined in a round-bottom flask which was submerged in an ice-bath. Methanesulfonyl chloride (458 mg, 4.00 mmol) was added to a small volume of THF (5mL) which was then and added to the cooled THF solution dropwise with stirring. The solution, which turned quickly from transparent to cloudy, was left to stir and warm up gradually to room temperature overnight. The next day, the cloudy white reaction mixture was

concentrated by rotary evaporation (**warning:** extra care must be taken to ensure no detectable peroxides are present in inhibitor-free THF prior to rotary evaporation) and the dry residue was dissolved in CH_2Cl_2. This solution was washed with HCl (2 M, 20 mL x 3) using a centrifuge to separate the aqueous and organic layers after shaking together. The combined HCl layers were then washed three times with CH_2Cl_2. All the CH_2Cl_2 layers were combined, dried ($MgSO_4$), filtered, concentrated by rotary evaporation and dried under vacuum overnight yielding the final product – namely **Pluronic bis(mesylate)** (4.93 g, 828 µmol, 92% yield) as a colourless gel-like solid. A molar mass of 5956 g/mol was used for Pluronic bis(mesylate) to calculate the yield.
^{1}H NMR (500 MHz, C_2D_6SO) δ 4.30 (t, J = 4.4 Hz, 4H), 3.67 (t, J = 4.4 Hz, 4H), 3.57 – 3.22 (m, ~403H), 3.18 – 3.15 (m, 6H), 1.06 – 0.98 (m, 208H).

Scheme S2 | Synthesis of Pluronic bis(azide)

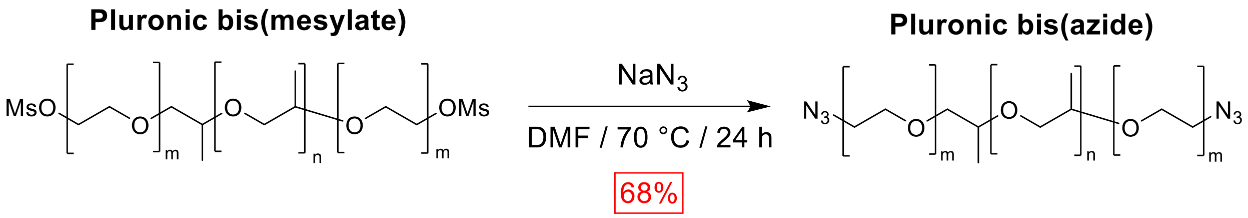

Pluronic Bis(azide): Pluronic bis(mesylate) (2.50 g, 420 µmol), NaN_3 (150 mg, 2.31 mmol) and anhydrous DMF (10 mL) were combined in a round-bottom flask and the reaction mixture heated overnight at 70 °C under an N_2 blanket (**warning:** care must be taken to ensure complete removal of CH_2Cl_2 from Pluronic bis(mesylate) prior to this reaction because NaN_3 can react with CH_2Cl_2 to form explosive derivatives[6]). The next day, the cooled cloudy white reaction solution was added to a saturated aqueous solution of $NaHCO_3$ (20 mL) and CH_2Cl_2 (20 mL). The organic and aqueous layers were separated by centrifugation and the aqueous layer was washed with CH_2Cl_2 (3 × 20 mL) (**warning:** it has been reported that the combination of inorganic azides and CH_2Cl_2, even for workups[6], risks the formation of explosive derivatives. We suggest THF as a possible alternative organic phase. Note that good separation of THF will require the aqueous phase to contain a high concentration of salts). The combined organic layers were then washed with brine, dried ($MgSO_4$), filtered, concentrated by rotary evaporation and left to dry under vacuum for 48 h to yield the final product – namely **Pluronic bis(azide)** (1.67 g, 285 µmol, 68%) as a colourless gel-like solid. A molar mass of 5850 g/mol was used for Pluronic bis(azide) to calculate the yield.
^{1}H NMR (500 MHz, CD_3SOCD_3) δ 3.61 – 3.22 (m, 391H), 1.06 – 0.96 (m, 208H).

Scheme S3 | Synthesis of Pluronic-BP•6TFA

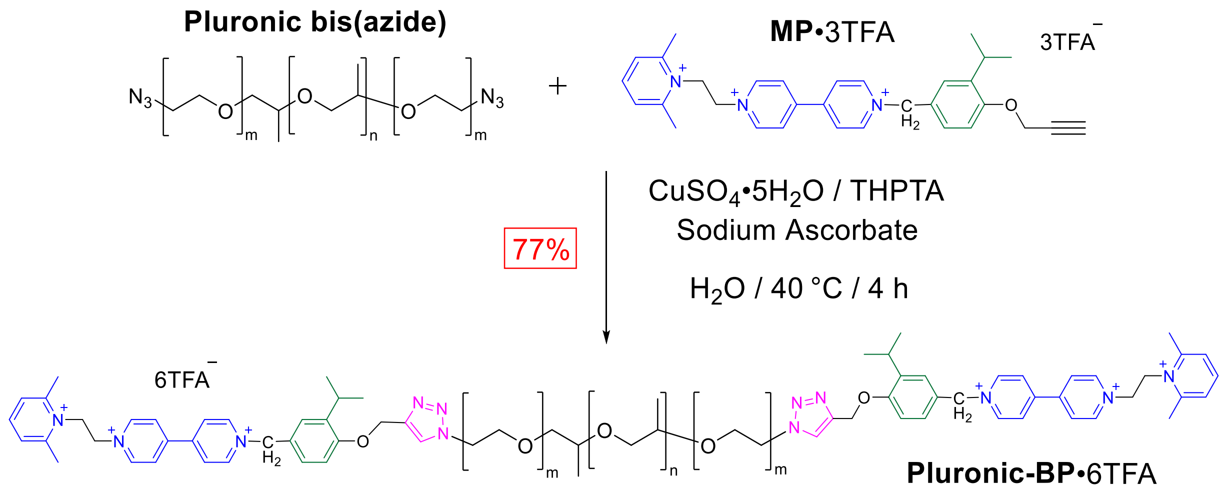

Pluronic-BP•6TFA: Pluronic bis(azide) (234 mg, 40 µmol), **MP**•3TFA (105 mg, 128 µmol), $CuSO_4$•$5H_2O$ (2 mg, 8 µmol), sodium ascorbate (5 mg, 25 µmol) and tris(3-hydroxypropyltriazolylmethyl)amine (THPTA) (5 mg, 12 µmol) were added to a 20-mL microwave reactor vial. THPTA was chosen as the ligand based on publications reporting its use[7] in water-based copper-catalyzed azide-alkyne cycloaddition "click" reactions. To the microwave vial containing the solid reagents was added de-ionized H_2O (10 mL) that had been sparged with N_2 for 40 min. A microwave cap containing a septum was crimp-sealed onto the microwave vial. A syringe was passed through the septum and the vial was exposed to vacuum before being flushed with N_2 for a total of three cycles. The syringe was removed and the vial vortexed and sonicated to ensure full dissolution of polymer prior to reaction. The vial was heated at 40 °C for 4 h in a microwave reactor. An aliquot of the crude reaction mixture was withdrawn, dried and analyzed by ^{1}H NMR spectroscopy to confirm that the reaction had gone to completion. The crude product was dried under air flow then purified using reversed-phase column chromatography. The dissolved crude product was wet-loaded into an equilibrated 30g C18 column and the eluent composition began with a 1:1 ratio of H_2O (containing 0.1% v/v TFA) and acetonitrile (containing

0.1% v/v TFA). The proportion of acetonitrile (0.1% TFA) in the eluent was increased gradually until it reached 100%, at which point pure **Pluronic-BP**•6TFA began to elute. The fractions containing the product were dried thoroughly, yielding the final product **Pluronic-BP**•6TFA (230 mg, 30.7 µmol, 77% yield) as an oily red/yellow solid. A molar mass of 7485 g/mol was used for **Pluronic-BP**•6TFA for calculation of the yield.

^{1}H NMR (500 MHz, D_2O) δ 9.30 (d, J = 6.5 Hz, 4H), 9.19 (d, J = 6.5 Hz, 4H), 8.70 (d, J = 6.6 Hz, 4H), 8.60 (d, J = 6.6 Hz, 4H), 8.38 (t, J = 8.0 Hz, 2H), 8.20 (s, 2H), 7.91 (d, J = 8.0 Hz, 4H), 7.52 (d, J = 2.2 Hz, 2H), 7.45 (dd, J = 8.5, 2.2 Hz, 2H), 7.31 (d, J = 8.4 Hz, 2H), 5.90 (s, 4H), 5.37 – 5.31 (m, 12H), 4.66 (t, J = 4.9 Hz, 4H), 3.99 (t, J = 5.0 Hz, 4H), 3.93 – 3.38 (m, 512H, **PEG/PPG**), 3.28 (p, J = 6.9 Hz, 2H), 2.94 (s, 12H), 1.27 – 1.08 (m, 317H, **PPG**).

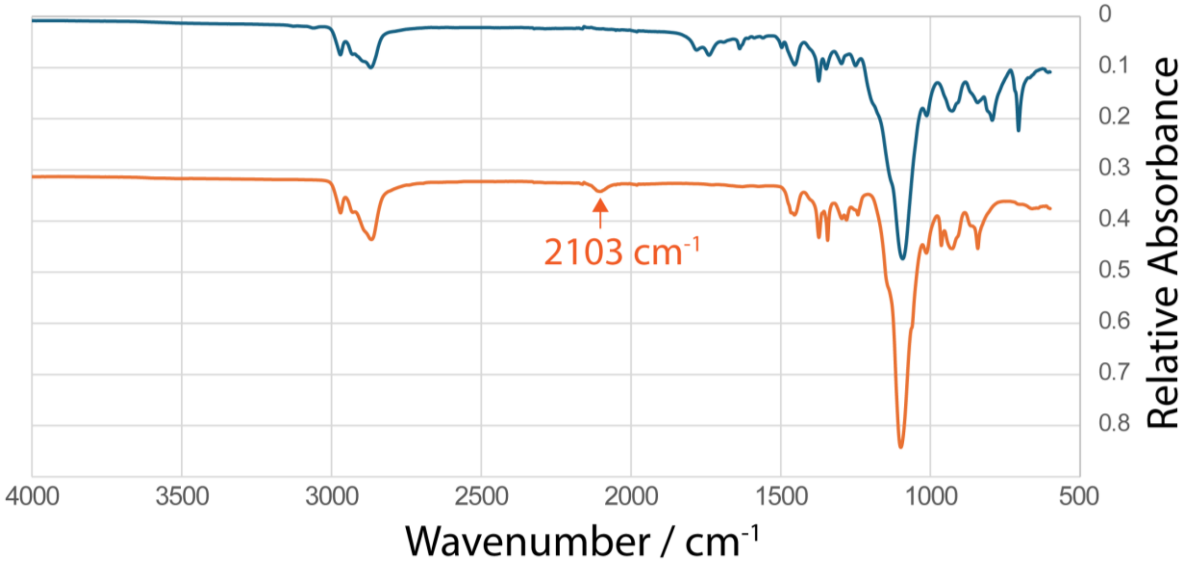

Figure S1 | FTIR traces of **Pluronic-BP**•6TFA (top, blue trace) and **Pluronic bis(azide)** (bottom, orange trace), providing evidence for the synthesis and isolation of the former. The traces have been vertically offset for clarity. The peak at 2103 cm^{-1} for Pluronic bis(azide) is attributed to the azide stretch and is absent from the trace of **Pluronic-BP**•6TFA.

Scheme S4 | Synthesis of OligoEG-BP•6TFA

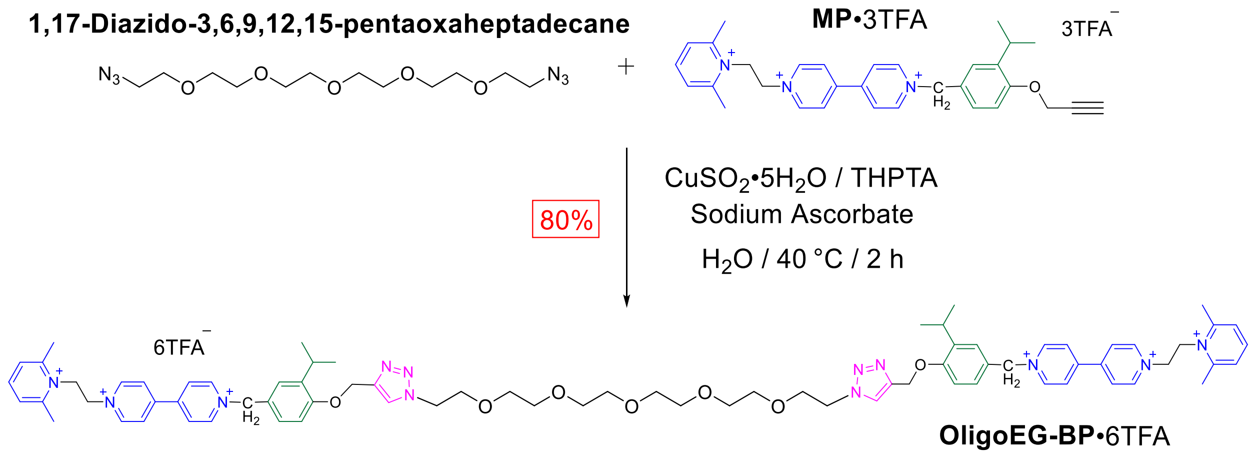

OligoEG-BP•6TFA: 1,17-Diazido-3,6,9,12,15-pentaoxaheptadecane (5.2 mg, 15.6 μmol), **MP**•3TFA (32 mg, 36 μmol), $CuSO_4$•$5H_2O$ (0.8 mg, 3 μmol), sodium ascorbate (1.2 mg, 6 μmol) and tris(3-hydroxypropyltriazolylmethyl)amine (THPTA) (2 mg, 5 μmol) were added to a 2-mL microwave reactor vial. THPTA was chosen following a publication reporting its use[7] in water-based copper-catalyzed azide-alkyne cycloaddition "click" reactions. To the microwave vial containing the solid reagents was added de-ionized H_2O (2 mL) that had been sparged with N_2 for 40 min. A microwave cap bearing a septum was crimp-sealed onto the microwave vial. A syringe was passed through the septum and the vial was exposed to vacuum then flushed with nitrogen, and this vacuum-nitrogen cycle was repeated two more times. In a microwave reactor, the vial was heated at 40 °C for 2 h. An aliquot of the crude reaction mixture was withdrawn, dried and analyzed by ^{1}H NMR spectroscopy to confirm that the reaction had gone to completion. The crude product was dried under air flow then purified using reversed-phase column chromatography. The dissolved crude product was wet-loaded into an equilibrated 80g C18 column. The eluent used was H_2O (containing 0.1% v/v TFA) with a gradually increasing gradient of acetonitrile (containing 0.1% v/v TFA). The fractions containing the product were dried thoroughly, yielding the final product **OligoEG-BP**•6TFA (24.7 mg, 12.6 μmol, 80% yield) as a sticky, translucent yellow solid.

1H NMR (600 MHz, D_2O) δ 9.28 (d, J = 7.0 Hz, 4H), 9.16 (d, J = 7.0 Hz, 4H), 8.68 (d, J = 6.9 Hz, 4H), 8.58 (d, J = 6.9 Hz, 4H), 8.36 (t, J = 7.9 Hz, 2H), 8.12 (s, 2H), 7.89 (d, J = 7.9 Hz, 4H), 7.46 (d, J = 2.4 Hz, 2H), 7.39 (dd, J = 8.5, 2.4 Hz, 2H), 7.20 (d, J = 8.5 Hz, 2H), 5.86 (s, 4H), 5.36 – 5.29 (m, 8H), 5.22 (s, 4H), 4.58 (t, J = 4.9 Hz, 4H), 3.91 (t, J = 4.9 Hz, 4H), 3.58 – 3.48 (m, 16H), 3.18 (p, J = 7.0 Hz, 2H), 2.92 (s, 12H), 1.09 (d, J = 7.0 Hz, 12H).

HRMS-ESI (m/z). Calculated for $[C_{88}H_{96}F_{18}N_{12}O_{19} – 2TFA]^{2+}$: 870.3458. Found: 870.3472.

Scheme S5 | Synthesis of OligoEG-OR2•14Cl

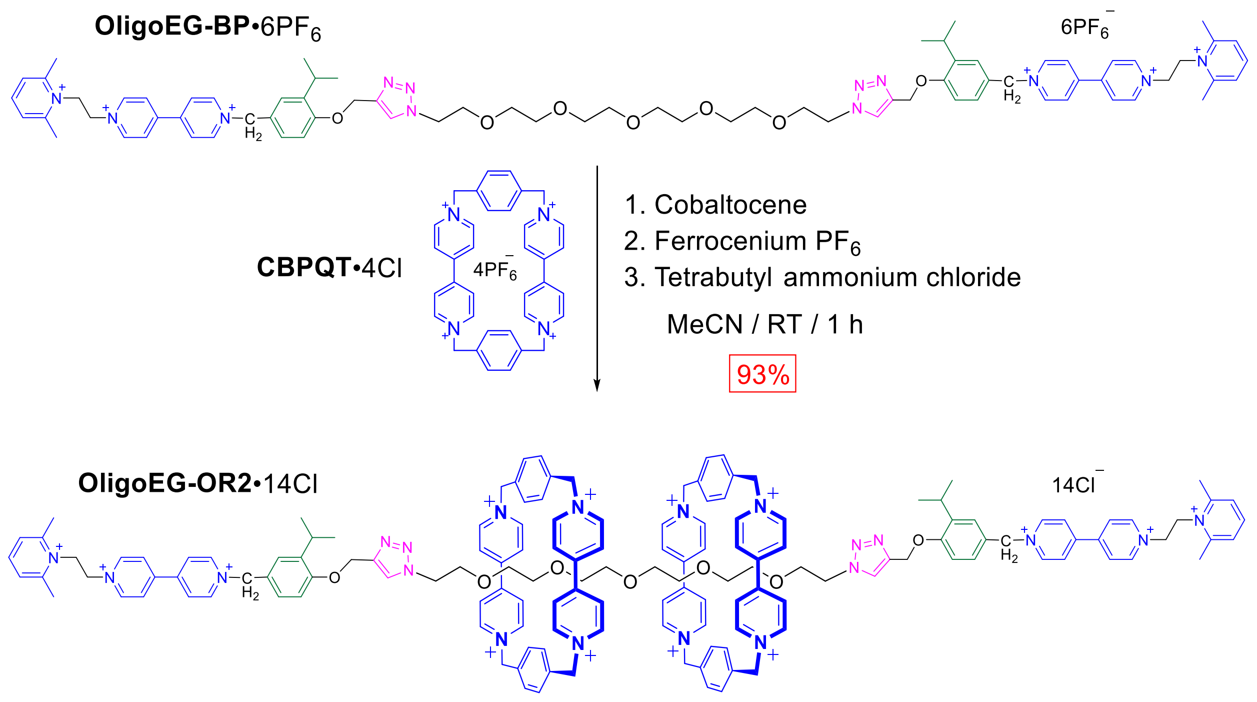

OligoEG-OR2•14Cl: Synthesis and isolation of **OligoEG-OR2** was performed in acetonitrile using **MP** and **CBPQT** as their PF_6 salts. This solvent and counterion combination allowed the use of higher concentrations of **CBPQT** (compared with the chloride salt in water), which in the reduced state as its PF_6 salt in acetonitrile does not precipitate out of solution below a concentration of ~10 mg/mL. **OligoEG-BP**•6PF_6 (19 mg, 8.8 µmol) and **CBPQT**•4PF_6 (40 mg, 36.3 µmol) were added to a 4-mL vial and placed in a N_2-flushed glove box. The solids were dissolved in anhydrous acetonitrile (3 mL). A stock solution of cobaltocene (18.8 mg, 99.4 µmol) in acetonitrile was added to the reaction mixture, bringing the total reaction volume to ~4.5 mL. The amount of cobaltocene used was a 10% excess over the amount sufficient to reduce each viologen unit in the reaction mixture once: this quantity of reducing agent was shown[8,9] in previous work to result in efficient pumping. After addition of cobaltocene the reaction solution turned an opaque, metallic purple colour and was left to stir for 30 min. Ferrocenium hexafluorophosphate (33 mg, 99.7 umol) was

added to the solution, which turned an opaque brown colour. The solution was left to stir for 30 min. A saturated solution of tetrabutyl ammonium chloride in acetontrile was added to the solution and a yellow precipitate formed. The precipitate was washed with acetonitrile twice, dissolved in methanol, then trifluoroacetic acid (~20 drops) was added to the solution, which was concentrated by rotary evaporation. The dissolved crude product was wet-loaded onto an equilibrated 30g C18 column and purified by reversed-phase column chromatography. The eluent used was H_2O (containing 0.1% v/v TFA) with a gradually increasing gradient of acetonitrile (containing 0.1% v/v TFA). The product typically elutes with an eluent composed of 20% of the acetonitrile solution. A saturated solution of KPF_6 in water was added to the fractions containing the product, yielding a precipitate which was washed twice with water. This precipitate was dissolved in acetonitrile and to that a saturated solution of tetrabutyl ammonium chloride in acetonitrile was added. The precipitate formed was washed with acetonitrile twice and dried, yielding the final product **OligoEG-OR2**•14Cl (24 mg, 2898.24 g/mol, 8.2 μmol, 93% yield) as a powdery yellow solid.

^{1}H NMR (600 MHz, D_2O) δ 9.33 (d, J = 7.1 Hz, 4H), 9.31 (d, J = 7.0 Hz, 4H), 9.27 (d, J = 6.9 Hz, 16H), 8.77 (d, J = 6.9 Hz, 4H), 8.71 (d, J = 6.9 Hz, 4H), 8.38 (t, J = 8.0 Hz, 2H), 8.17 (d, J = 7.0 Hz, 16H), 7.91 (d, J = 8.1 Hz, 5H), 7.88 (s, 17H), 7.67 (d, J = 2.2 Hz, 2H), 7.61 (dd, J = 8.4, 2.2 Hz, 2H), 6.17 (d, J = 8.4 Hz, 2H), 6.05 (s, 4H), 6.00 – 5.93 (m, 16H), 5.88 (s, 2H), 5.39 – 5.34 (m, 8H), 4.25 (t, J = 5.1 Hz, 4H), 3.86 (t, J = 5.1 Hz, 4H), 3.72 – 3.68 (m, 4H), 3.57 – 3.53 (m, 4H), 3.48 – 3.38 (m, 8H), 2.96 (s, 12H), 2.83 (p, J = 6.8 Hz, 2H), 2.67 (s, 4H), 1.32 (d, J = 6.9 Hz, 12H).

HRMS-ESI (m/z). Calculated for $[C_{148}H_{160}F_{84}N_{20}O_7P_{14} - 2PF_6]^{2+}$: 2034.9252. Found: 2034.9136. Calculated for $[C_{148}H_{160}F_{84}N_{20}O_7P_{14} - 3PF_6]^{3+}$: 1308.2952. Found: 1308.2937.

Scheme S6 | Synthesis of OligoEG-OR1•10Cl

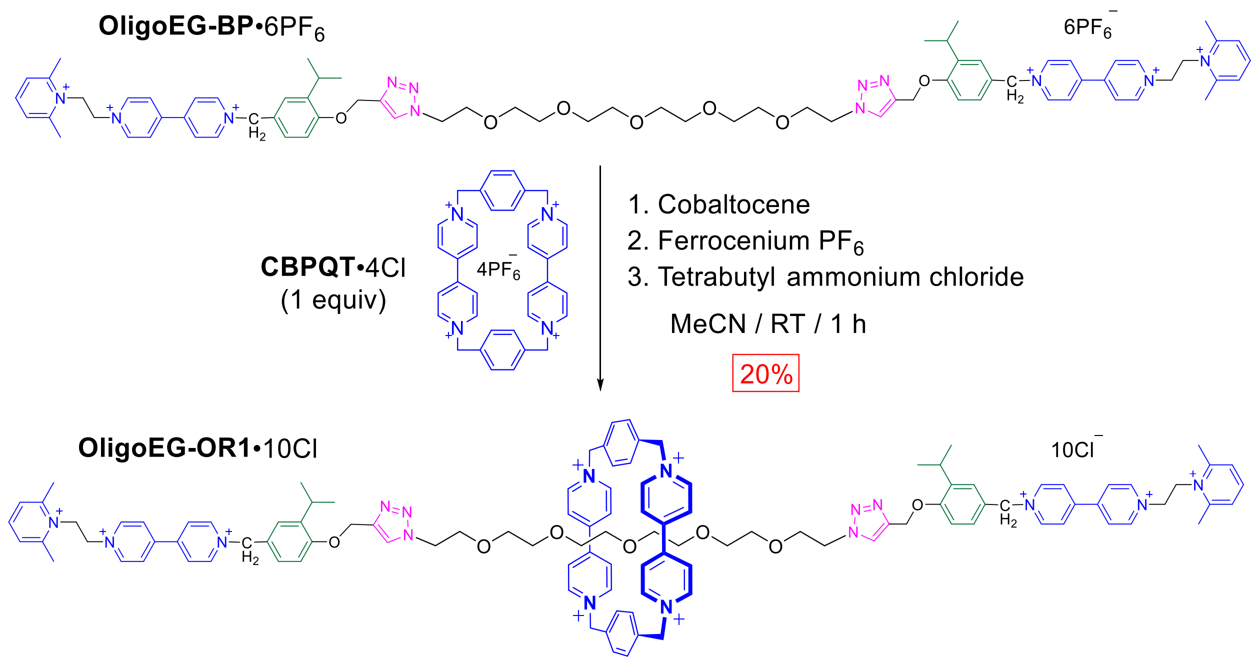

OligoEG-OR1•10Cl: Synthesis and isolation of **OligoEG-OR1** was performed in acetonitrile using **MP** and **CBPQT** as their PF_6 salts. **OligoEG-BP**•6PF_6 (14.8 mg, 6.9 µmol) and **CBPQT**•4PF_6 (7.7 mg, 7.0 µmol) were added to a 4-mL vial and placed in a N_2-flushed glove box. The solids were dissolved in anhydrous acetonitrile (1 mL). A stock solution of cobaltocene (5.8 mg, 30.5 µmol) in acetonitrile was added to the reaction mixture, bringing the total reaction volume to ~1.5 mL. The amount of cobaltocene used was a 10% excess over the amount sufficient to reduce each viologen unit in the reaction mixture once: this quantity of reducing agent was shown[8,9] in previous work to result in efficient pumping. After addition of cobaltocene the reaction solution turned an opaque, metallic purple colour and was left to stir for 30 min. Ferrocenium hexafluorophosphate (10.1 mg, 30.5 umol) was added to the solution, which turned an opaque brown colour. The solution was left to stir for 30 min. A saturated solution of tetrabutyl

ammonium chloride in acetontrile was added to the solution and a yellow precipitate formed. The precipitate was washed with acetonitrile twice, dissolved in methanol, following which trifluoroacetic acid (~10 drops) was added and the solution concentrated by rotary evaporation. The dissolved crude product was wet-loaded onto an equilibrated 30g C18 column and purified by reversed-phase column chromatography. The eluent used was H_2O (containing 0.1% v/v TFA) with a gradually increasing gradient of acetonitrile (containing 0.1% v/v TFA). The product typically elutes with an eluent composed of 15 – 20% of the acetonitrile solution. A saturated solution of KPF_6 in water was added to the fractions containing the product, yielding a precipitate which was washed twice with water. This precipitate was dissolved in acetonitrile and to that a saturated solution of tetrabutyl ammonium chloride in acetonitrile was added. The precipitate formed was washed with acetonitrile twice and dried, yielding the final product **OligoEG-OR1**•10Cl (3 mg, 2164.86 g/mol, 1.4 μmol, 20% yield) as a yellow solid.

^{1}H NMR (600 MHz, D_2O) δ 9.31 (d, *J* = 6.7 Hz, 4H), 9.27 (d, *J* = 6.6 Hz, 8H), 9.21 (d, *J* = 6.6 Hz, 4H), 8.71 (d, *J* = 6.7 Hz, 4H), 8.62 (d, *J* = 6.6 Hz, 4H), 8.38 (t, *J* = 7.9 Hz, 2H), 8.30 (d, *J* = 6.6 Hz, 8H), 7.91 (d, *J* = 8.0 Hz, 4H), 7.75 (s, 8H), 7.55 (d, *J* = 2.2 Hz, 2H), 7.50 (dd, *J* = 8.5, 2.2 Hz, 2H), 7.45 (s, 2H), 7.02 (d, *J* = 8.4 Hz, 2H), 5.96 – 5.90 (m, 12H), 5.37 – 5.32 (m, 8H), 4.58 (s, 4H), 4.47 (t, *J* = 5.1 Hz, 4H), 3.85 (t, *J* = 5.1 Hz, 4H), 3.41 – 3.35 (m, 4H), 3.19 – 3.08 (m, 6H), 2.96 – 2.92 (m, 16H), 2.88 – 2.83 (m, 4H), 1.19 (d, *J* = 7.0 Hz, 12H).

HRMS-ESI (m/z). Calculated for $[C_{112}H_{128}F_{60}N_{16}O_7P_{10} - 2PF_6]^{2+}$: 1484.8655. Found: 1484.8664. Calculated for $[C_{112}H_{128}F_{60}N_{16}O_7P_{10} - 3PF_6]^{3+}$: 941.5887. Found: 941.5865.

The Identification and Removal of Ether-Terminated Impurities from Pluronic

Allyl ether- and propenyl ether-terminated polymeric impurities are produced through side-reactions during base-catalyzed polymerizations of polypropylene oxide[10]. ^{1}H NMR spectroscopy, MALDI mass spectrometry and size-exclusion chromatography evinced the presence of such impurities in the commercial **Pluronic** sample used in this study. MALDI mass spectrometry (Section 8) confirmed the presence of polymeric ether-terminated impurities and indicates that those impurities span the approximate molecular weight range 1000 – 3000 g/mol with a maximum at 1604 g/mol. The simplest means for removing these impurities was found to be reversed-phase chromatography following end-capping of polymers with **MP**•3TFA. The commercial **Pluronic** starting material used in this study should therefore not be considered a sample of Pluronic P123 of M_n ~5800 but should instead be considered a sample containing a major species and a minor species: Pluronic triblock copolymer chains of M_n ~7100 (major) and ether-terminated polymeric impurities of M_n ~2000 (minor). The "123" in Pluronic P123 is a cipher that denotes the molecular weight of the PPG block and the weight percent of the PEG block[11,12]. The large Pluronic chains of M_n ~7100, which were purified from the starting material and used for the pumping studies in this work, have two PEG blocks of average length 25.5 and a PPG block of average length 83 and should therefore be termed Pluronic P152 rather than Pluronic P123.

The Fractionation of Pluronic-BP•6TFA

Fractionation of **Pluronic-BP**•6TFA occurred during purification by reversed-phase column chromatography. Early fractions contained **Pluronic-BP**•6TFA with relatively short PPG segments and late fractions contained chains with relatively long PPG segments. The composition of the fractionated polymers was determined by ^{1}H NMR spectroscopy.

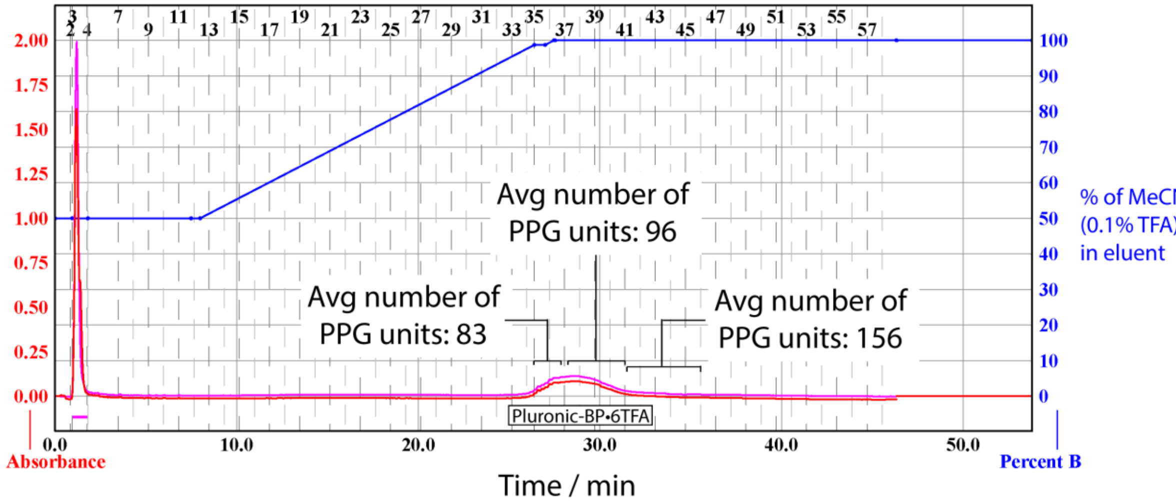

Figure S2 | Graph produced during reversed-phase column chromatography of **Pluronic-BP**•6TFA annotated with sample fractionation data. The purple (254 nm) and red (214 nm) traces represent UV absorbance of eluent and the black numbers along the top denote fraction number. The majority of obtained **Pluronic-BP**•6TFA had 80 – 100 repeat units comprising the PPG block of the chain.

4. Optimisation of Micellar Pump Operation

A variety of parameters were tested in order to find the optimal pump operation conditions. The concentration of 5.0 mg/mL **Pluronic-BP**•6TFA was chosen as it is sufficiently high to allow formation of micelles and to afford good resolution of all resonances by ^{1}H NMR spectroscopy. The concentration of **CBPQT**•4Cl in bulk solution was found to be a key parameter for achieving efficient pumping. Counterintuitively, too high a concentration of **CBPQT**•4Cl (>1.7 mg/mL, >2.57 mM) results in reduced pumping efficiency. It was found that **CBPQT**•4Cl, when dissolved by itself in D_2O, forms large aggregates in the reduced state above a certain concentration. This critical aggregation concentration lies between 1.74 mg/mL (2.63 mM)—at which no aggregates were observed and the reduced solution displays a deep blue colour—and 1.97 mg/mL (2.97 mM), when aggregates are observed by DLS and the solution displays a deep purple colour. Ultraviolet-visible-near-infrared (UV-Vis-NIR) spectrophotometry performed on solutions of purple aggregated **CBPQT**$^{\mathbf{2(+\bullet)}}$ revealed the existence of a NIR absorption peak in the range of λ = 914 to 929 nm, where the exact λ_{max} depends on the concentration of the rings. See Supporting Figures S36 & S37. This absorption peak is reminiscent[13–15] of the characteristic peak at ca. 900 nm occurring in aqueous solutions of reduced dimerised bipyridiniums. We hypothesised that the aggregation of **CBPQT**•4Cl in its reduced state is driven by intermolecular pimerisation[16] of the bipyridinium units. Since there are two bipyridinium units per **CBPQT**•4Cl ring, it is reasonable that pimerisation of **CBPQT**•4Cl would lead to the formation of extended multimeric aggregates,

rather than dimers as in the case of molecules containing individual bipyridinium units. The aggregation of **CBPQT**•4Cl was observed to have an onset critical concentration lower than 1.75 mg/mL in solutions in which other bipyridinium-containing species, such as **Pluronic-BP**•6TFA, are present. Pumping experiments were therefore performed with **CBPQT**•4Cl at a concentration of 1.5 mg/mL (2.26 mM).

Taking inspiration from the viologen literature[13], iodine (I_2) was used as a heterogenous oxidising agent on the reduced **Pre-Pump** solution. It led to a rapid colour change of the solution from purple to yellow and returned a pumping efficiency of 66%. In order to investigate the effect of speed of oxidation by air, a reduced **Pre-Pump** solution was left open to the atmosphere where it would be slowly oxidised. The solution lost its purple colour gradually over the course of a number of hours. The pumping efficiency measured for this slowly-oxidized **Post-Pump** solution was only 48%, indicating that the rate of oxidation by air plays an important role in the efficiency of pumping.

Condition	CBPQT concentration (mg/mL)	Oxidant	Pumping Efficiency (%)
Optimal (**Pre-Pump**)	1.5	I_2	66
Optimal (**Pre-Pump**)	1.5	Air sparge	65
Low-conc. **CBPQT**•4Cl	1.0	I_2	60
Slow oxidation	1.5	Air	48
High-conc. **CBPQT**•4Cl	2.0	Air sparge	30

Table S1 | Pumping efficiencies of various micellar solutions of Pluronic-BP•6TFA

Stability of the Post-Pump Solution

The **Post-Pump** solution, in which macrocycles are stored in micelles, was observed to be stable over the course of days. Since the molecular pump cassettes act as both the pumps and the stoppers for the pumped rings, we posit that only degradation of the pumping cassettes or the collecting chains would allow release of the rings back to free solution. Such a result has been observed in unimeric PPG-based polyrotaxanes degraded through prolonged heating[17].

5. NMR Spectroscopy

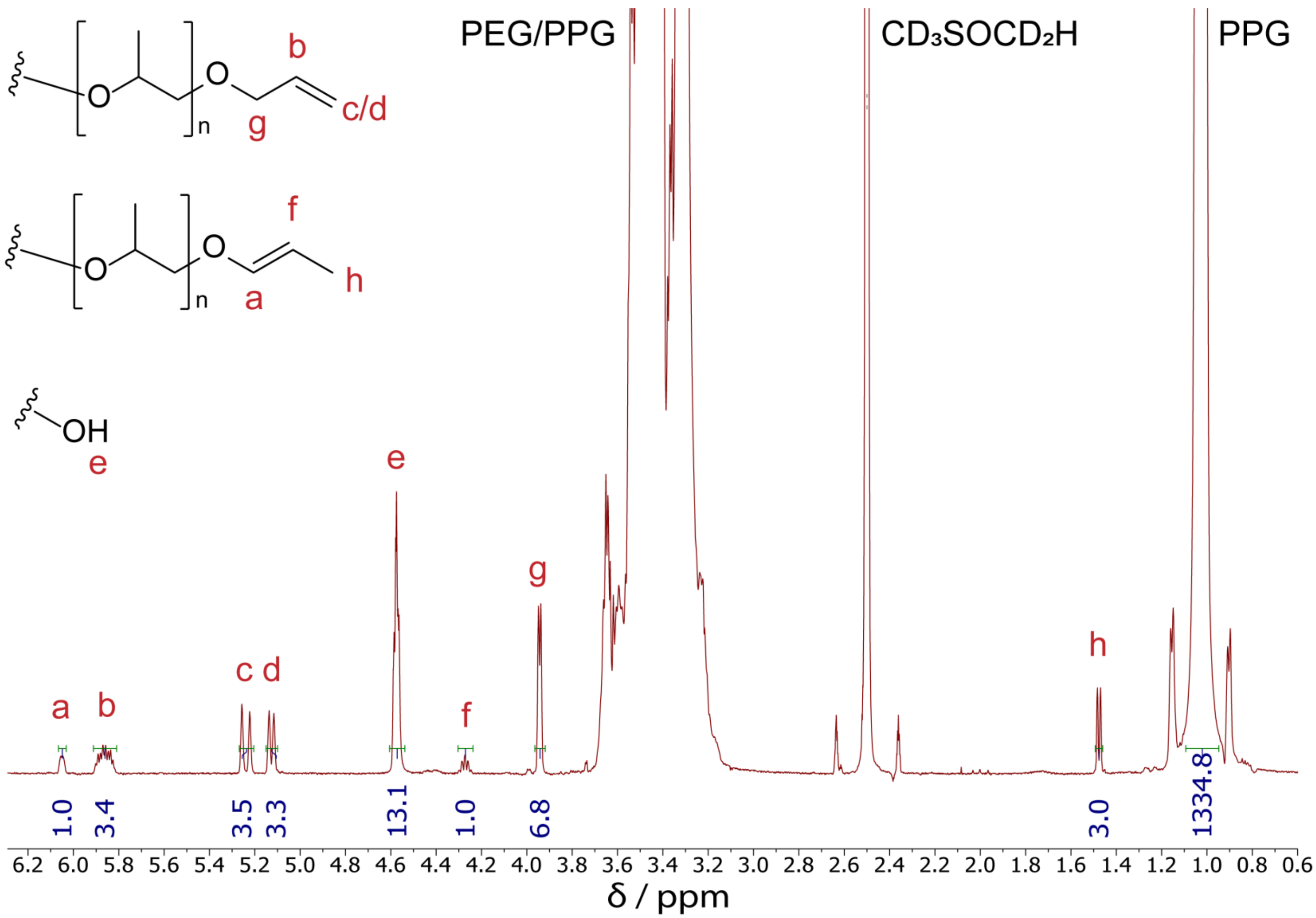

Figure S3 | Annotated and heavily magnified ^{1}H NMR spectrum of **Pluronic** (600 MHz, CD_3SOCD_3, 298 K), identifying minor impurities present in the sample.

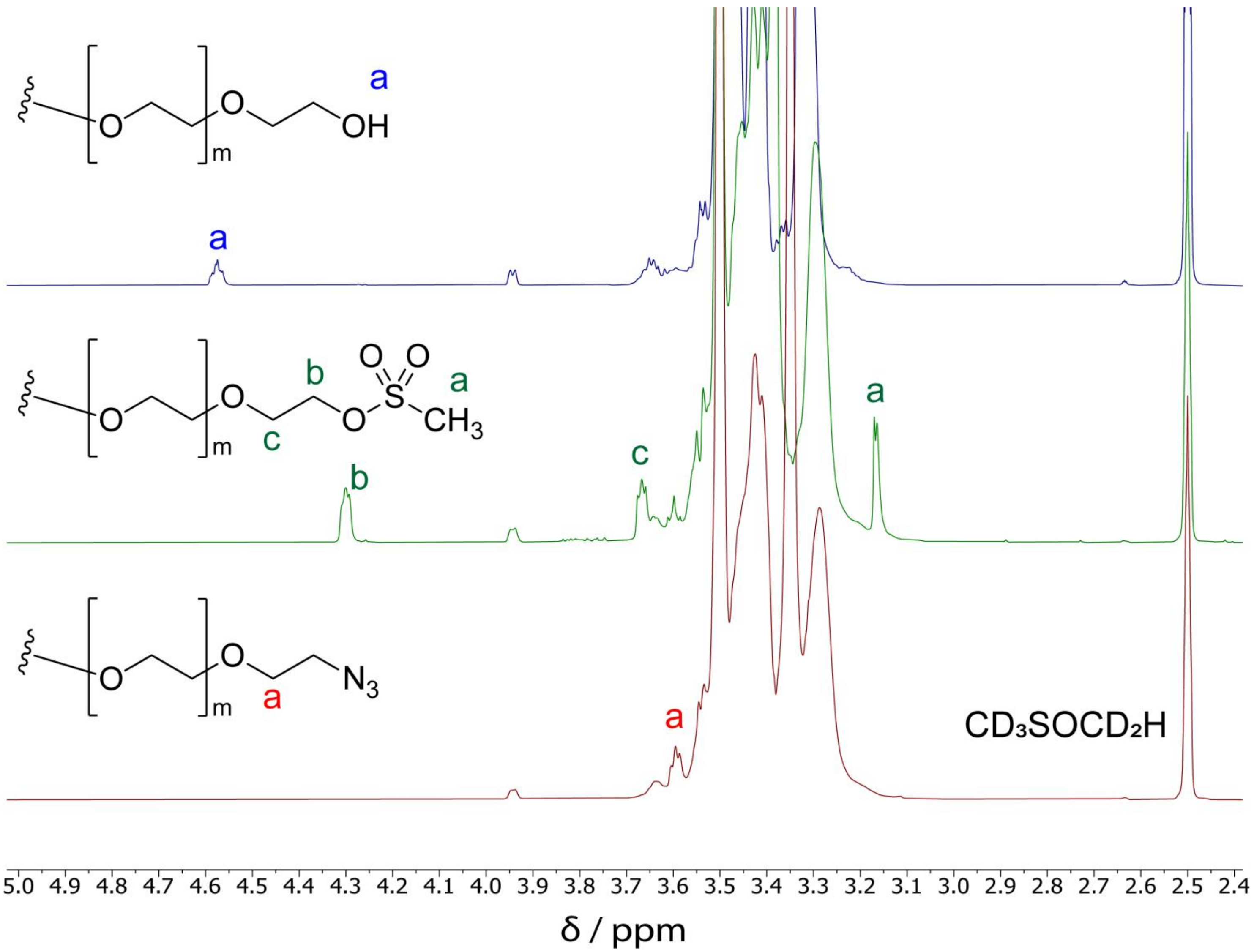

Figure S4 | Stacked annotated ^{1}H NMR spectra of **Pluronic** (blue), **Pluronic bis(mesylate)** (green) and **Pluronic bis(azide)** (red) (500 MHz, CD_3SOCD_3, 298 K). Assignments are made only distinct resonance peaks that uniquely identify each species. The large multiplet in the range 3.6 – 3.2 ppm corresponds to overlapping resonances for protons on the Pluronic backbone and in H_2O.

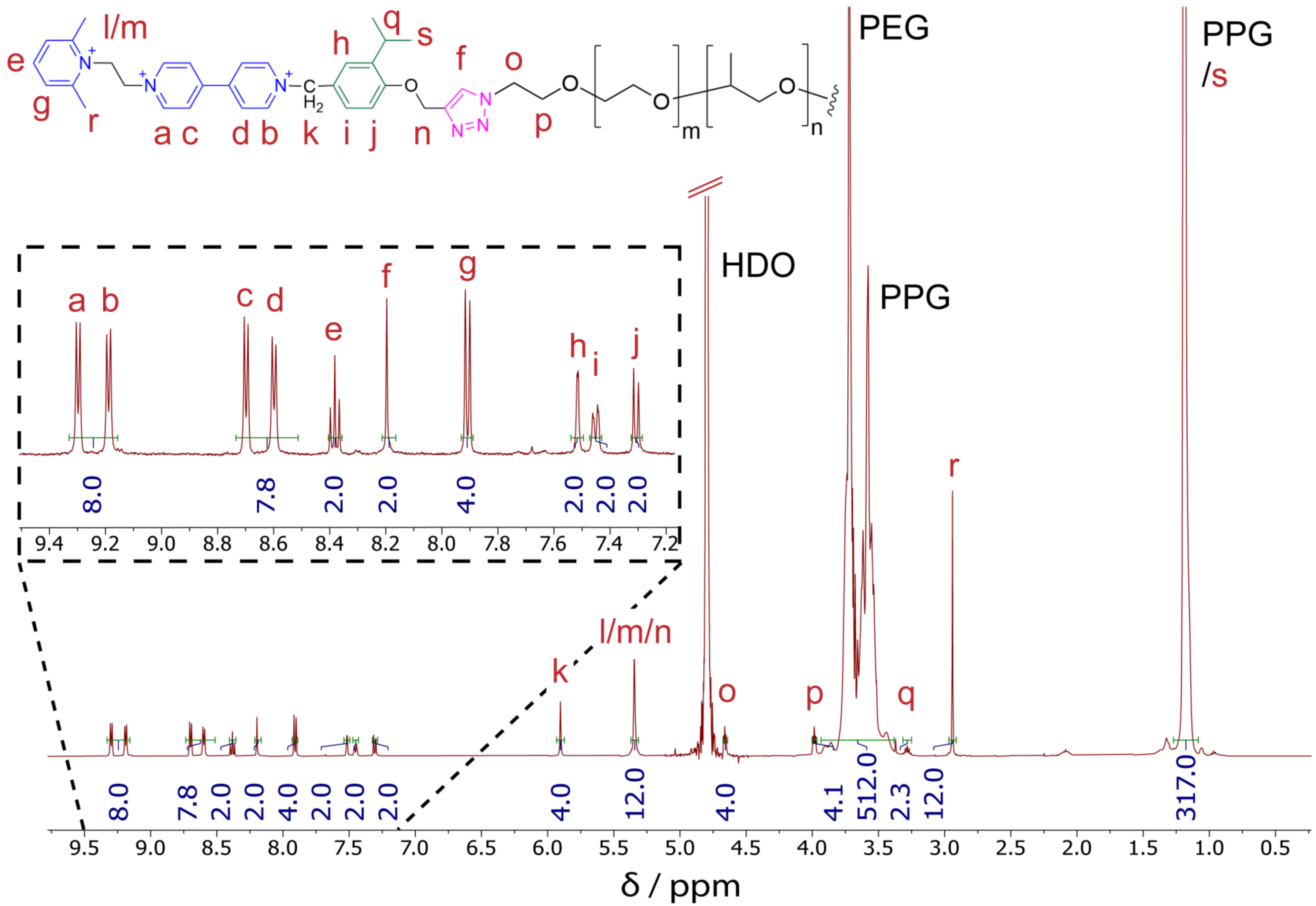

Figure S5 | Annotated ^{1}H NMR spectrum of **Pluronic-BP**•6TFA (600 MHz, D_2O, 298 K)

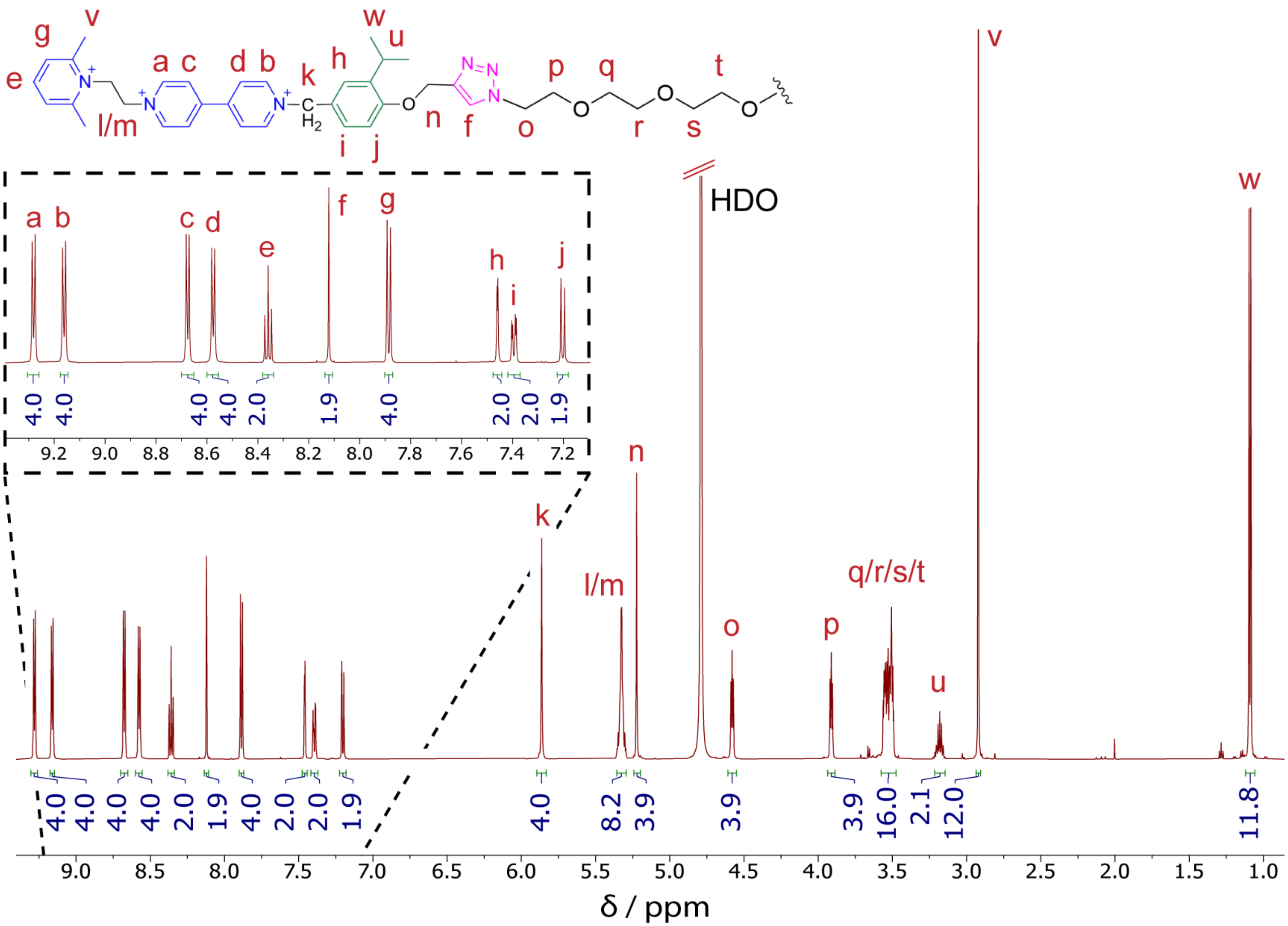

Figure S6 | Annotated ^{1}H NMR spectrum of **OligoEG-BP**•6TFA (600 MHz, D_2O, 298 K)

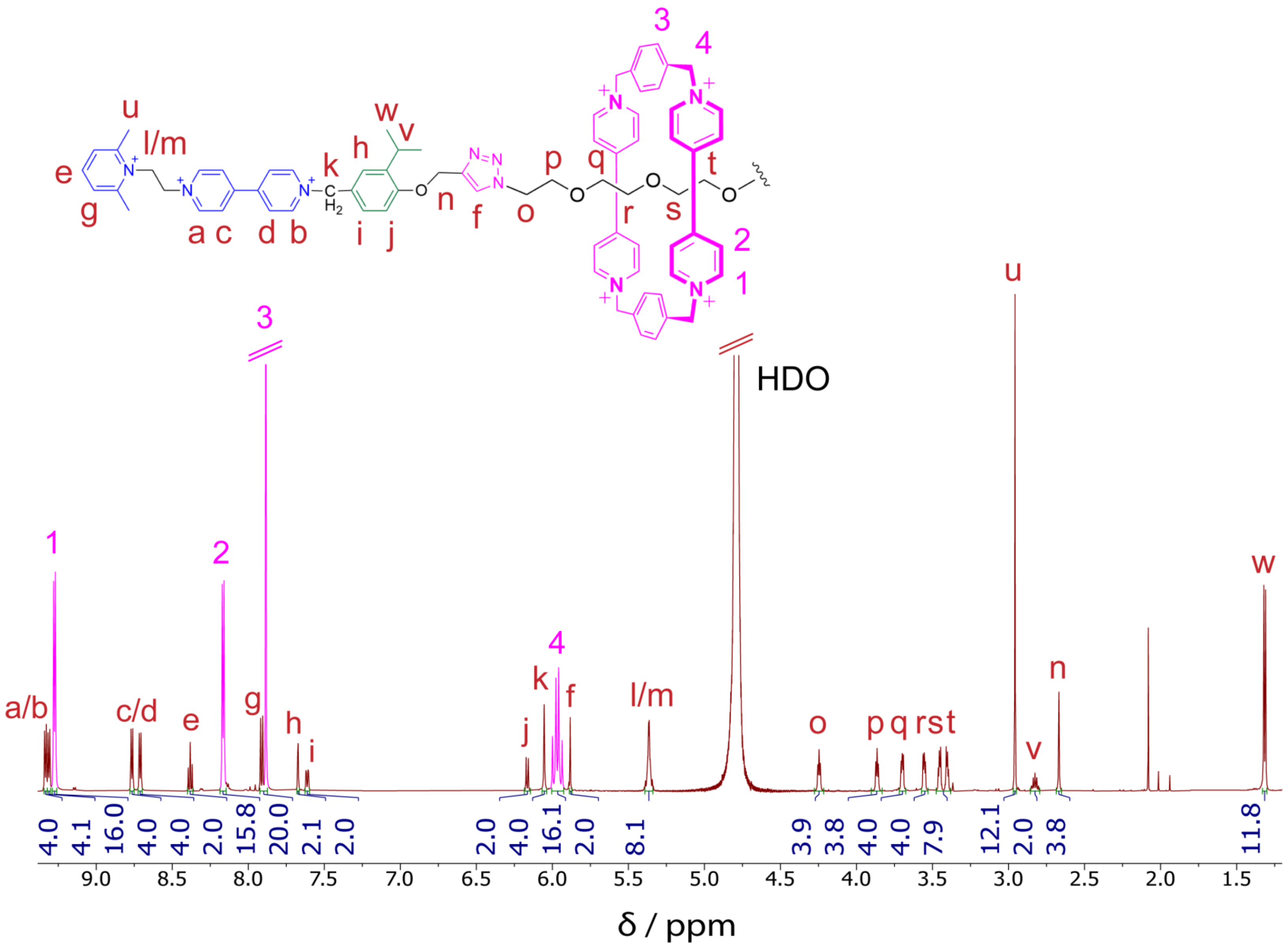

Figure S7 | Annotated ^{1}H NMR spectrum of **OligoEG-OR2**•14Cl (600 MHz, D_2O, 298 K)

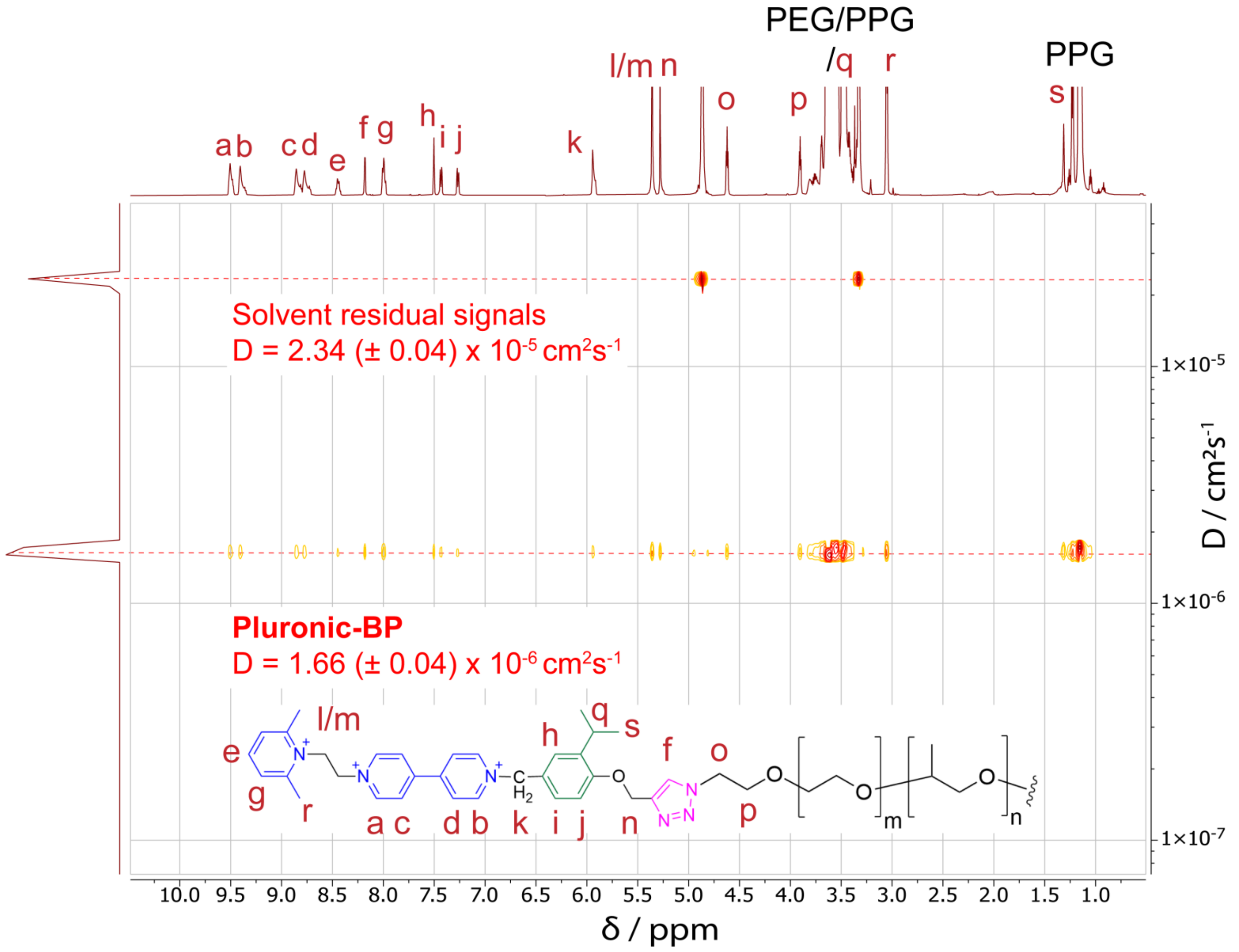

Figure S8 | ^{1}H DOSY NMR spectrum of nonmicellar **Pluronic-BP**•6TFA (600 MHz, CD_3OD, 298 K)

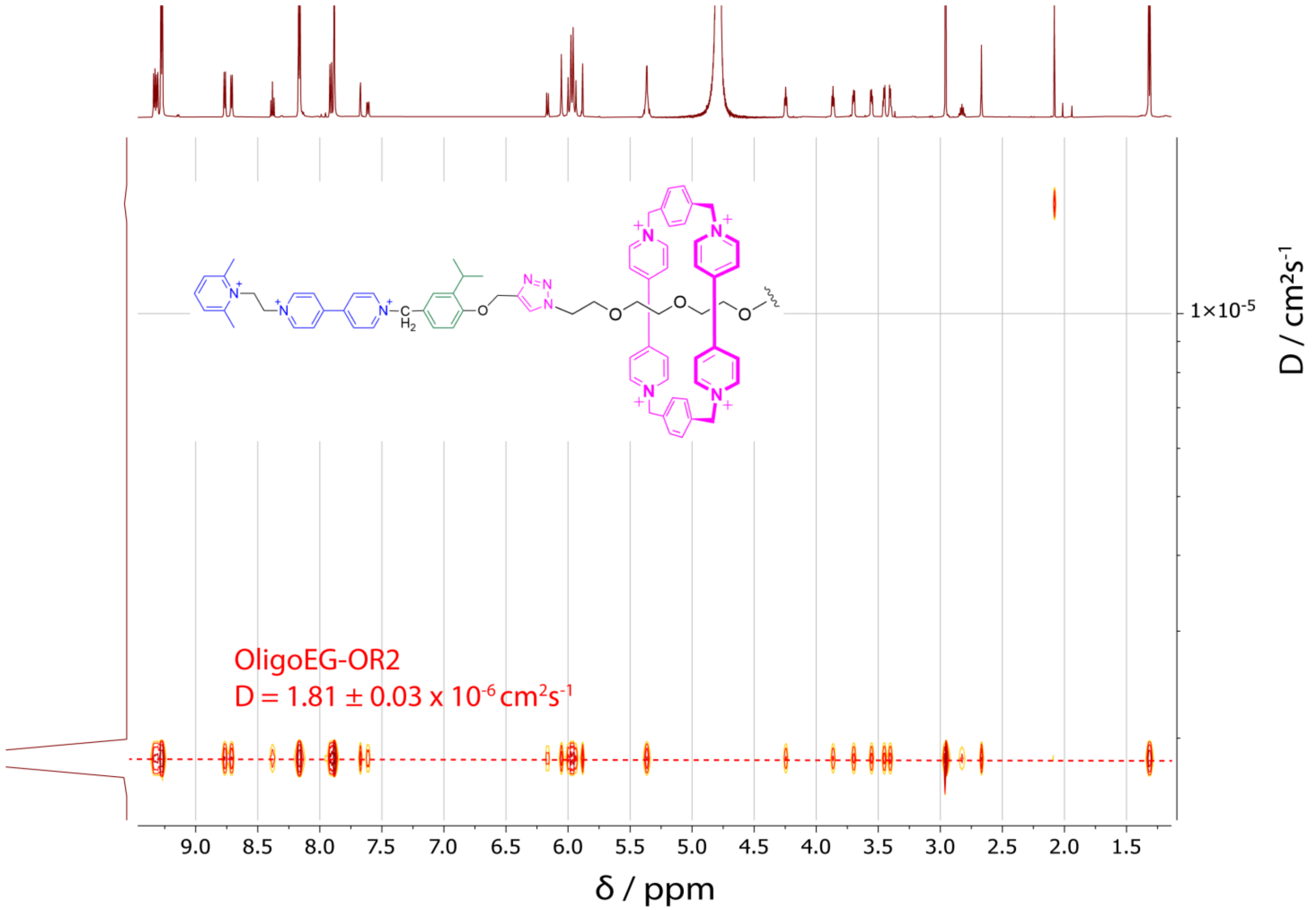

Figure S9 | ^{1}H DOSY NMR spectrum of **OligoEG-OR2**•14Cl (600 MHz, D_2O, 298 K). The crosspeaks marked by the dotted red line are assigned to **OligoEG-OR2**. The peaks corresponding to the molecular pumps and macrocycles diffuse at the same rate, indicating that they belong to the same molecule.

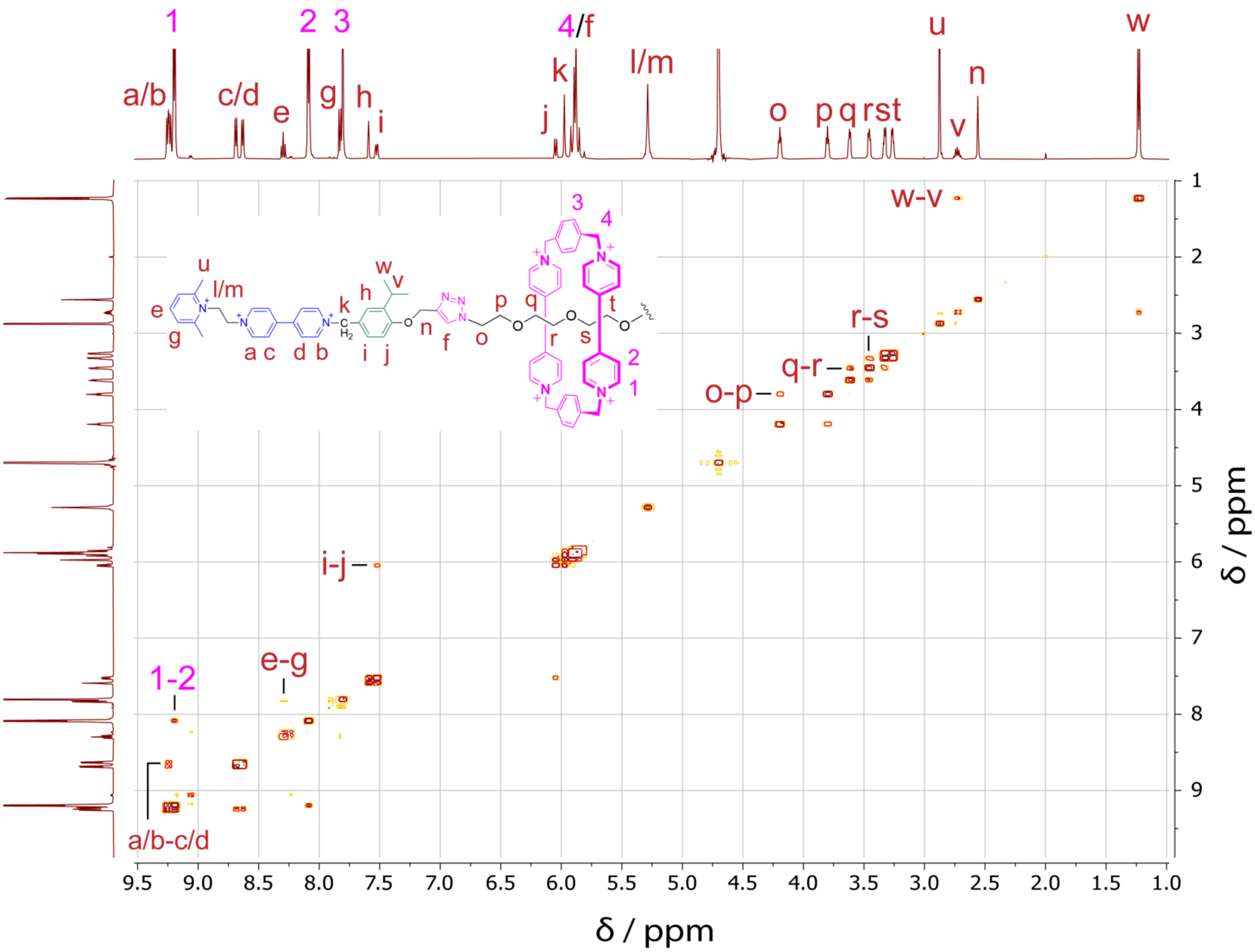

Figure S10 | Annotated ^{1}H-^{1}H COSY NMR spectrum of **OligoEG-OR2**•14Cl (500 MHz, D_2O, 298 K)

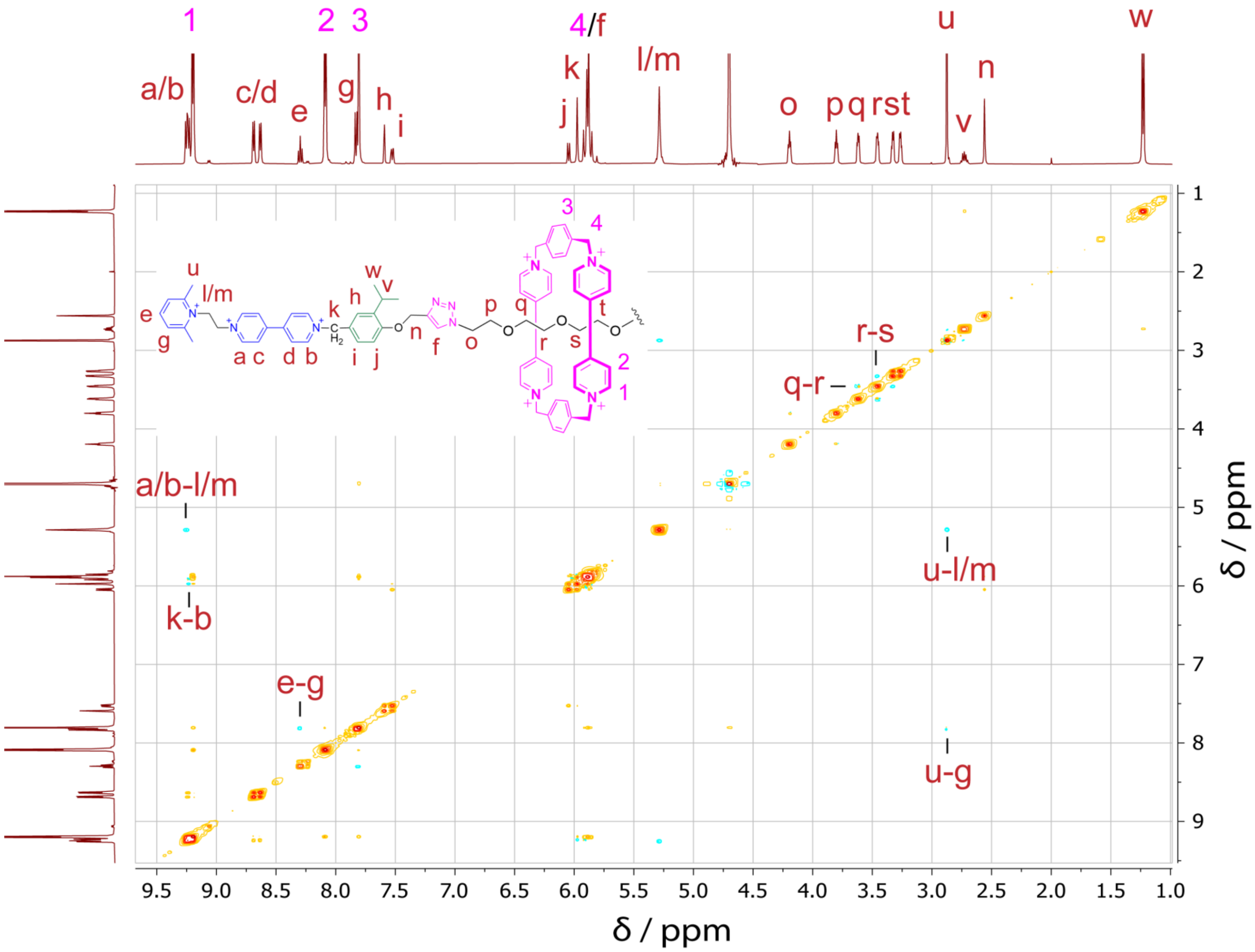

Figure S11 | Annotated ^{1}H-^{1}H NOESY NMR spectrum of **OligoEG-OR2**•14Cl (500 MHz, D_2O, 298 K)

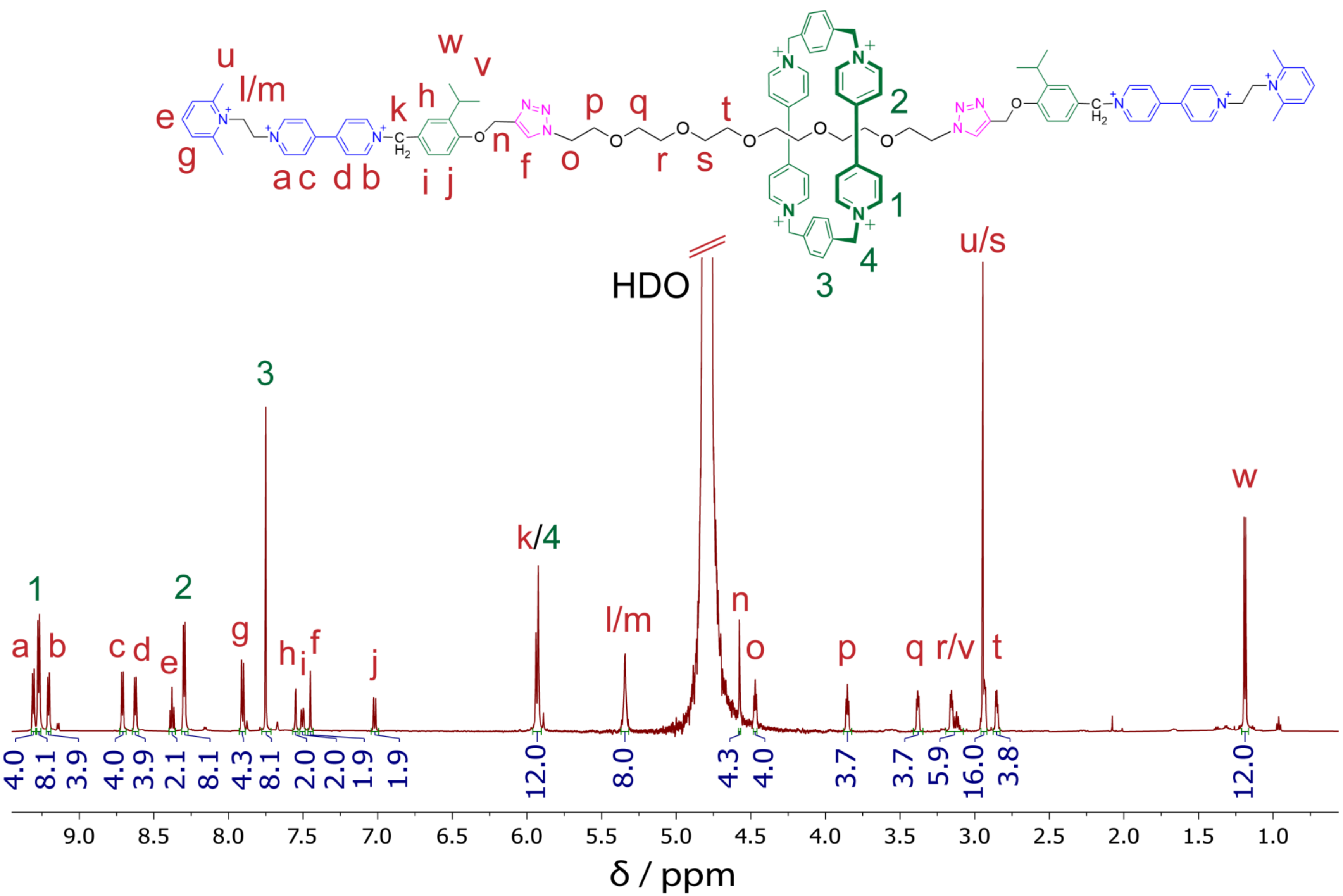

Figure S12 | Annotated ^{1}H NMR spectrum of **OligoEG-OR1**•10Cl (600 MHz, D_2O, 298 K)

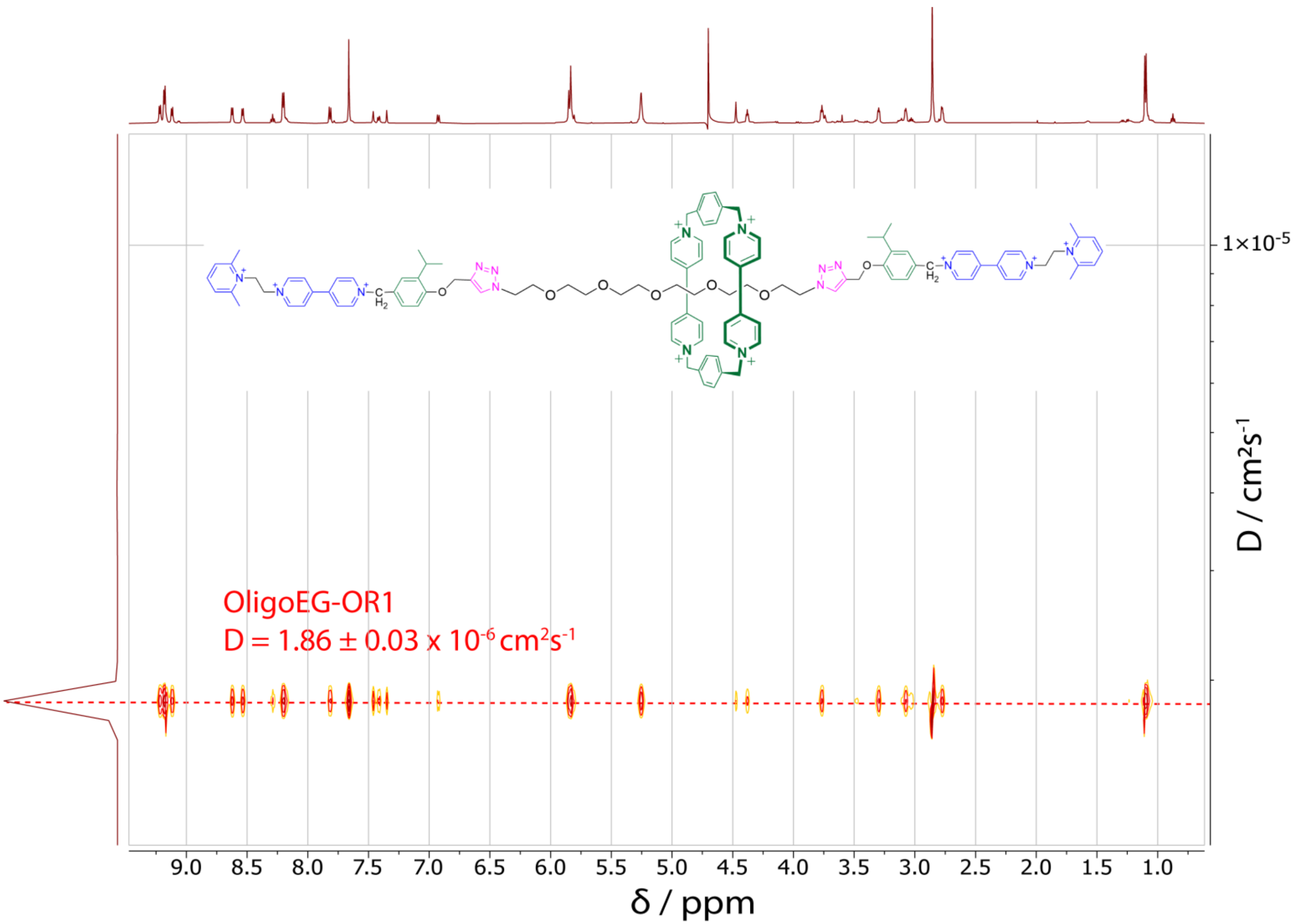

Figure S13 | ^{1}H DOSY NMR spectrum of **OligoEG-OR1**•10Cl (600 MHz, D_2O, 298 K). The crosspeaks marked by the dotted red line are assigned to **OligoEG-OR1**. The peaks corresponding to the molecular pumps and macrocycles diffuse at the same rate, indicating that they belong to the same molecule.

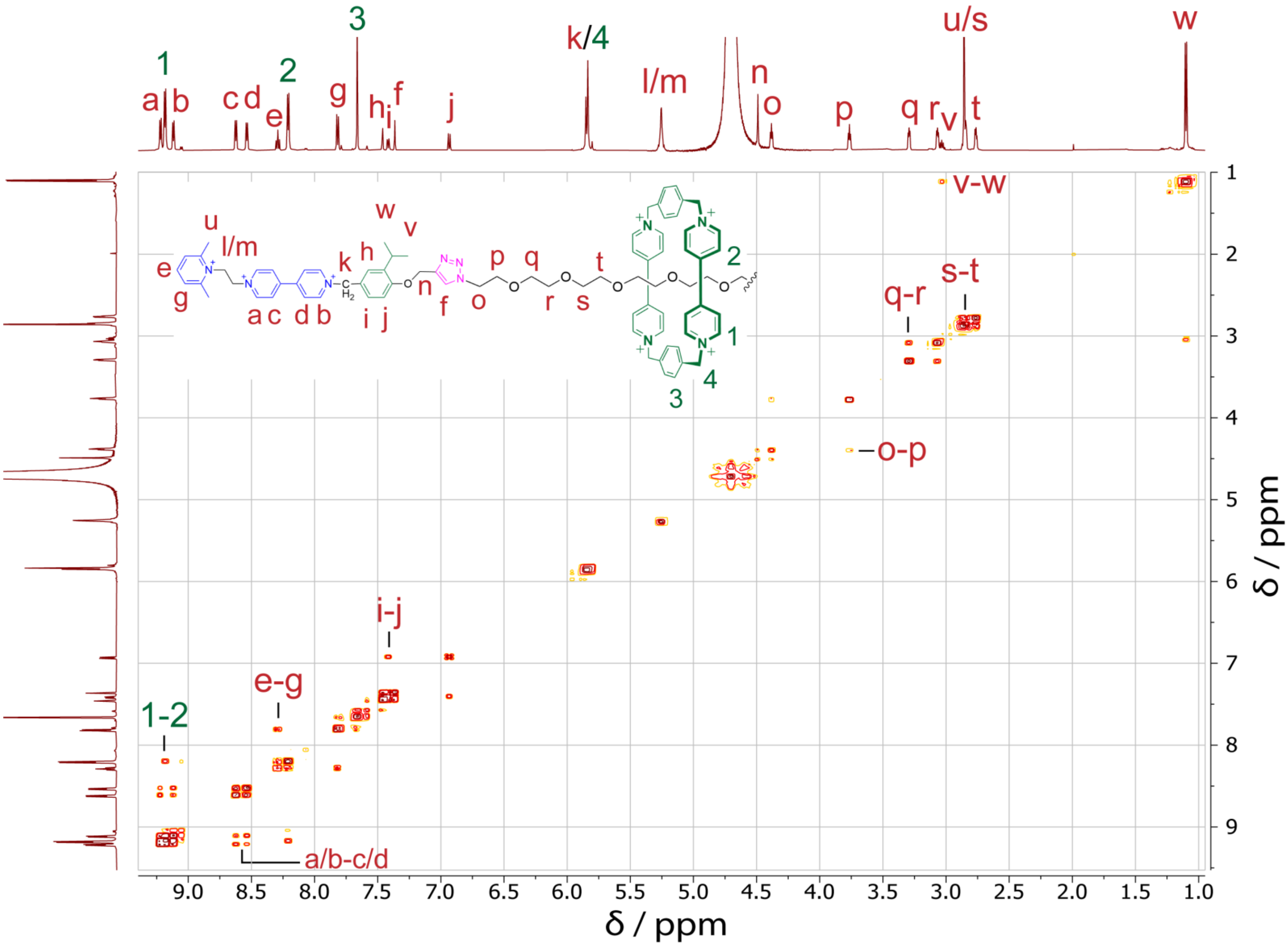

Figure S14 | Annotated ^{1}H-^{1}H COSY NMR spectrum of **OligoEG-OR1**•10Cl (600 MHz, D_2O, 298 K)

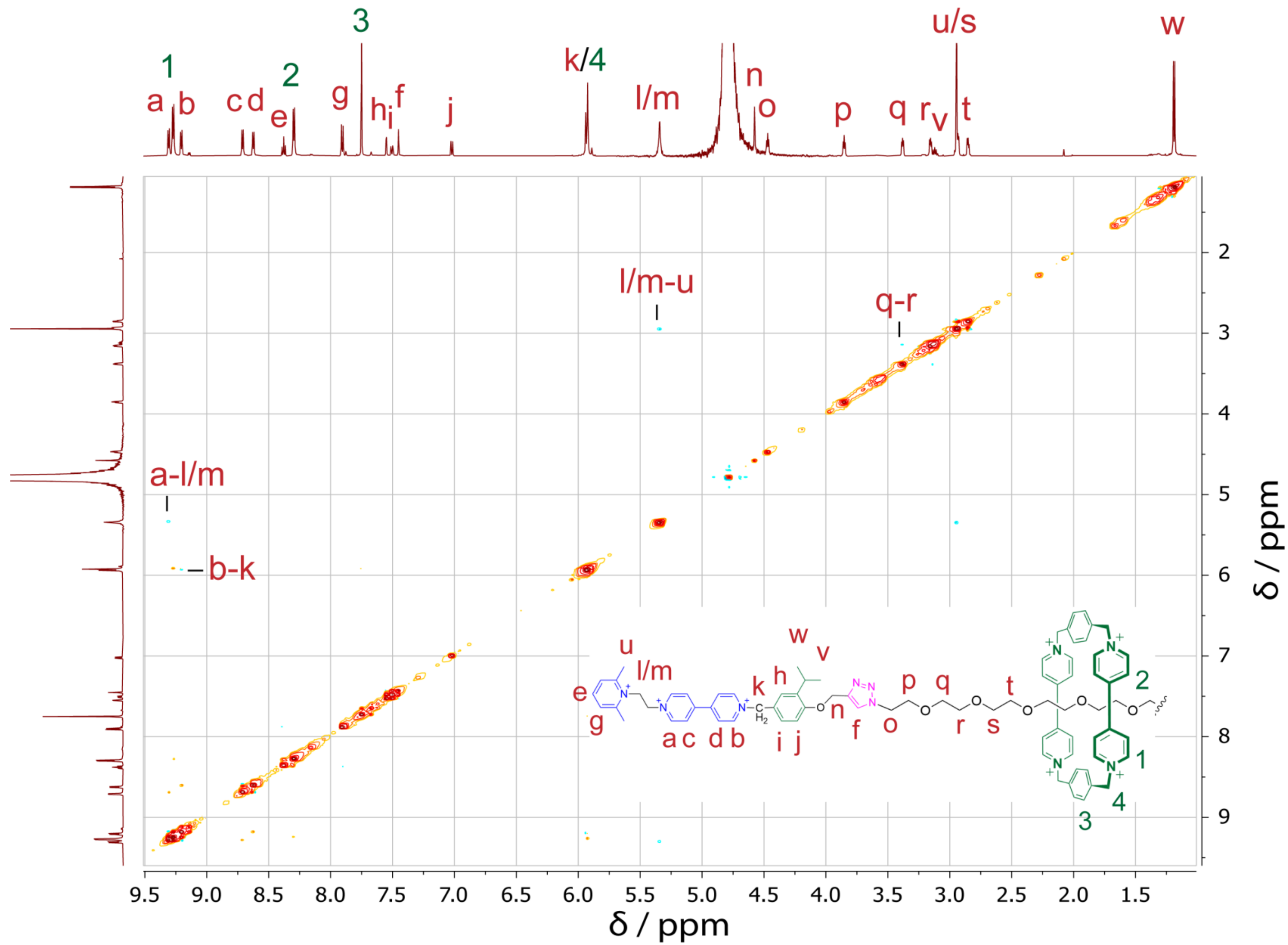

Figure S15 | Annotated ^1H-^1H NOESY NMR spectrum of **OligoEG-OR1**•10Cl (600 MHz, D_2O, 298 K)

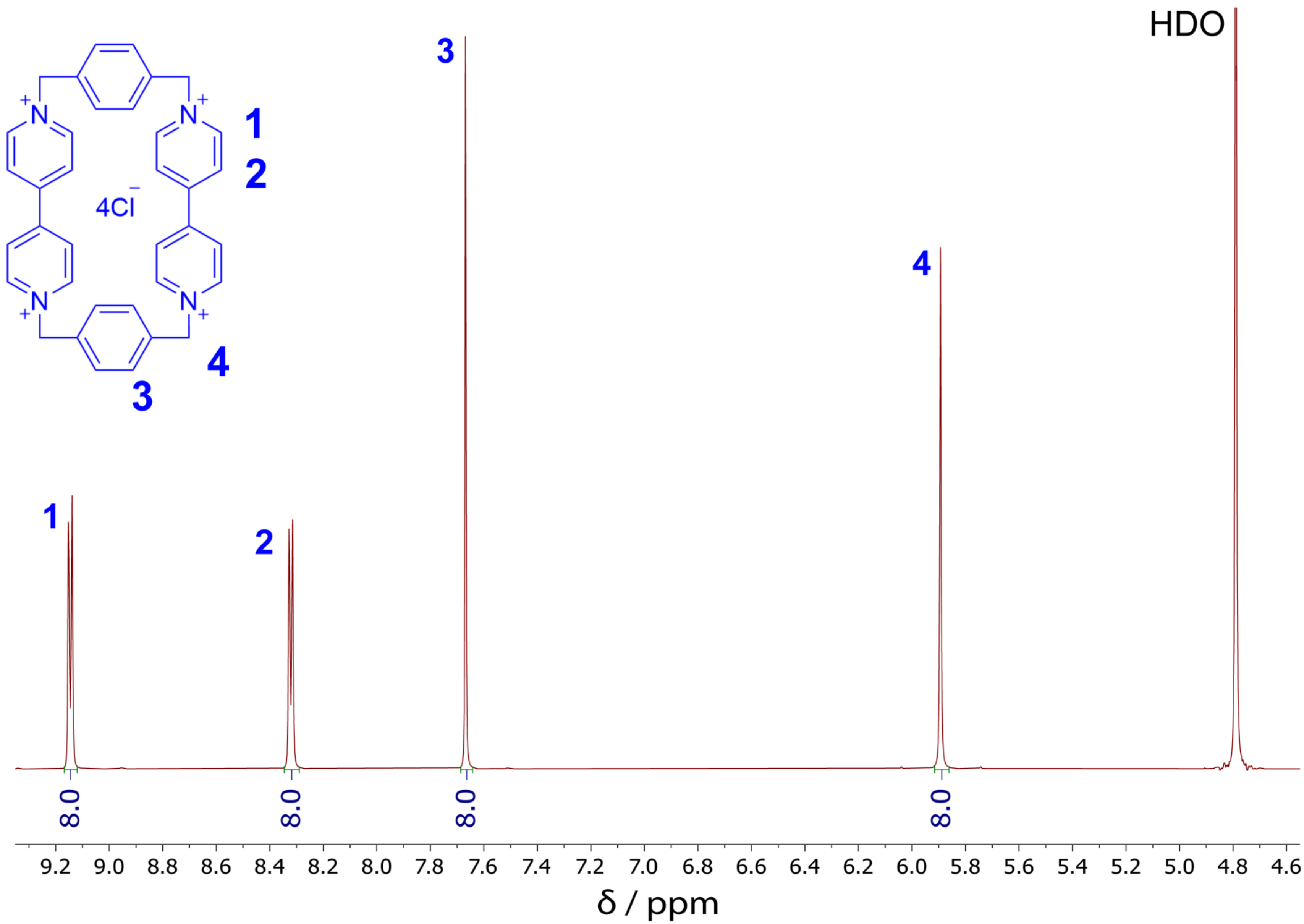

Figure S16 | Annotated ^1H NMR spectrum of **CBPQT**•4Cl (500 MHz, D_2O, 298 K)

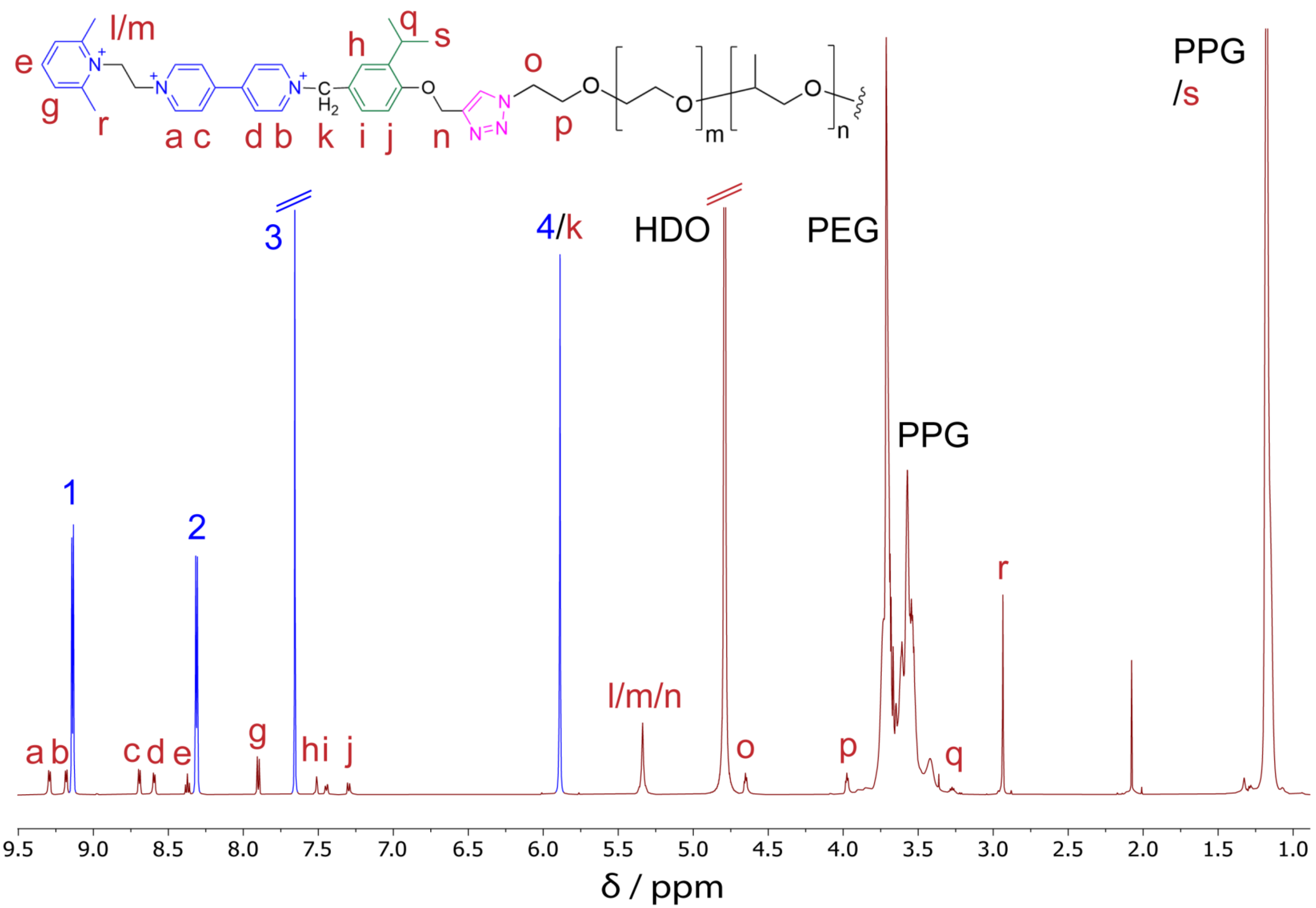

Figure S17 | Annotated ^{1}H NMR spectrum of a **Pre-Pump** micellar solution of **Pluronic-BP**•6TFA and **CBPQT**•4Cl (600 MHz, D_2O, 298 K). The resonance peaks corresponding to the protons of free **CBPQT**•4Cl are coloured blue.

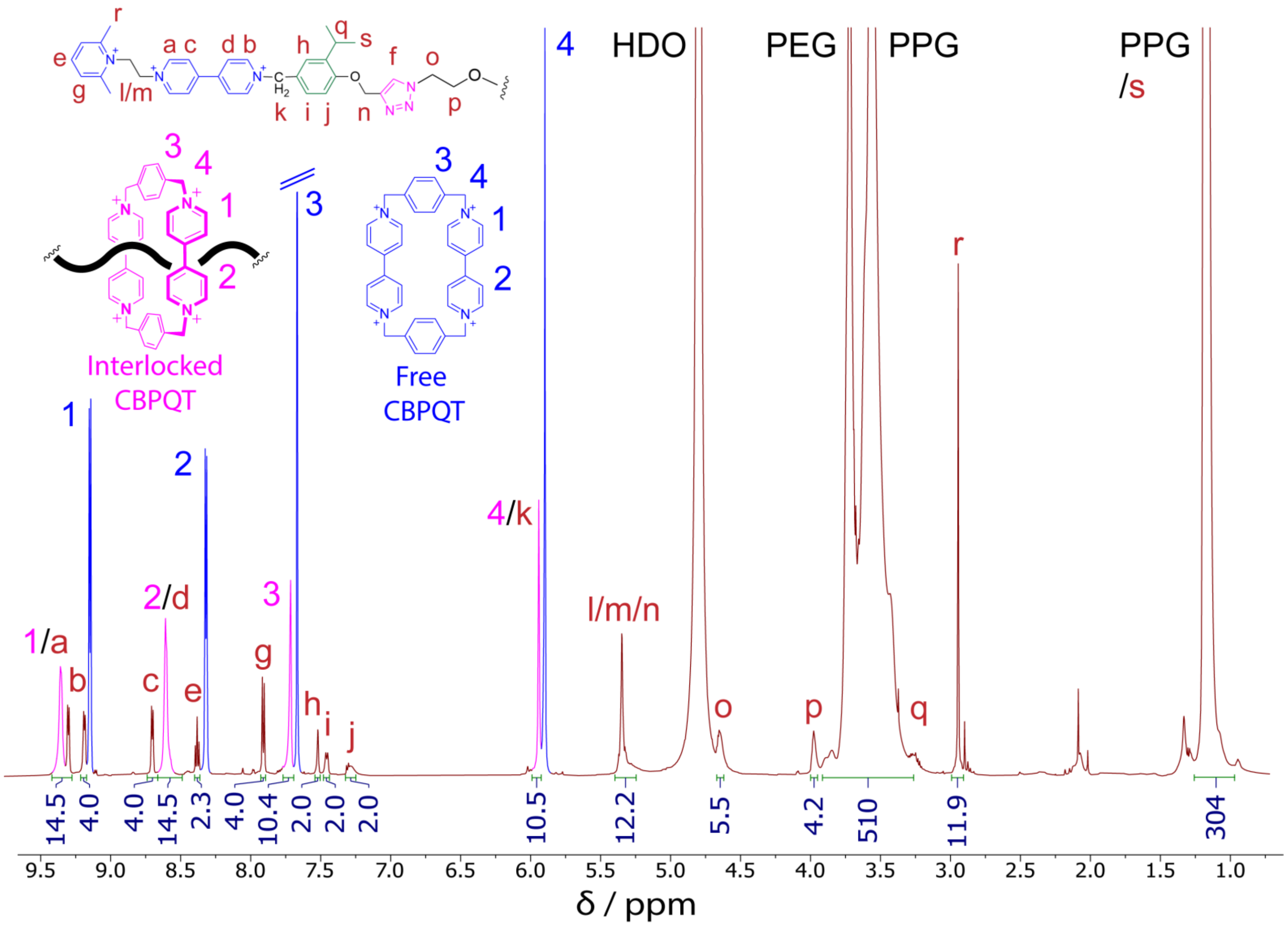

Figure S18 | Annotated ^{1}H NMR spectrum of the **Post-Pump** micellar solution of **Pluronic-BP**•6TFA and **CBPQT**•4Cl following oxidation by air flow (600 MHz, D_2O, 298 K). The resonance peaks corresponding to protons of interlocked **CBPQT**•4Cl are coloured magenta and those corresponding to free **CBPQT**•4Cl are coloured blue. The Pluronic polymer chain threading interlocked CBPQT is represented by a bold black line.

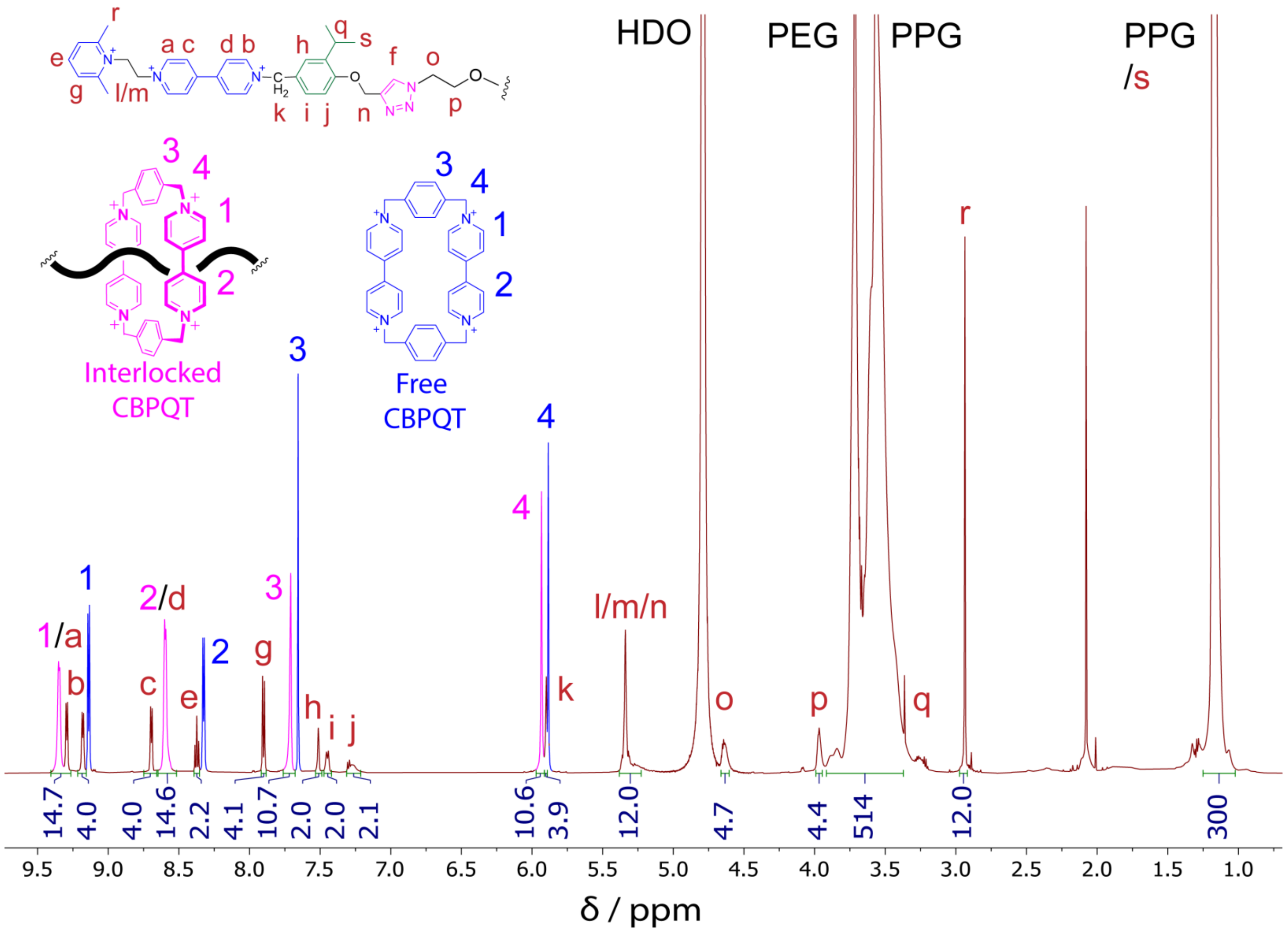

Figure S19 | Annotated ^{1}H NMR spectrum of the **Post-Pump** micellar solution of **Pluronic-BP**•6TFA and **CBPQT**•4Cl following oxidation with elemental I_2 (600 MHz, D_2O, 298 K). The resonance peaks corresponding to protons of interlocked **CBPQT**•4Cl are coloured magenta and those corresponding to free **CBPQT**•4Cl are coloured blue.

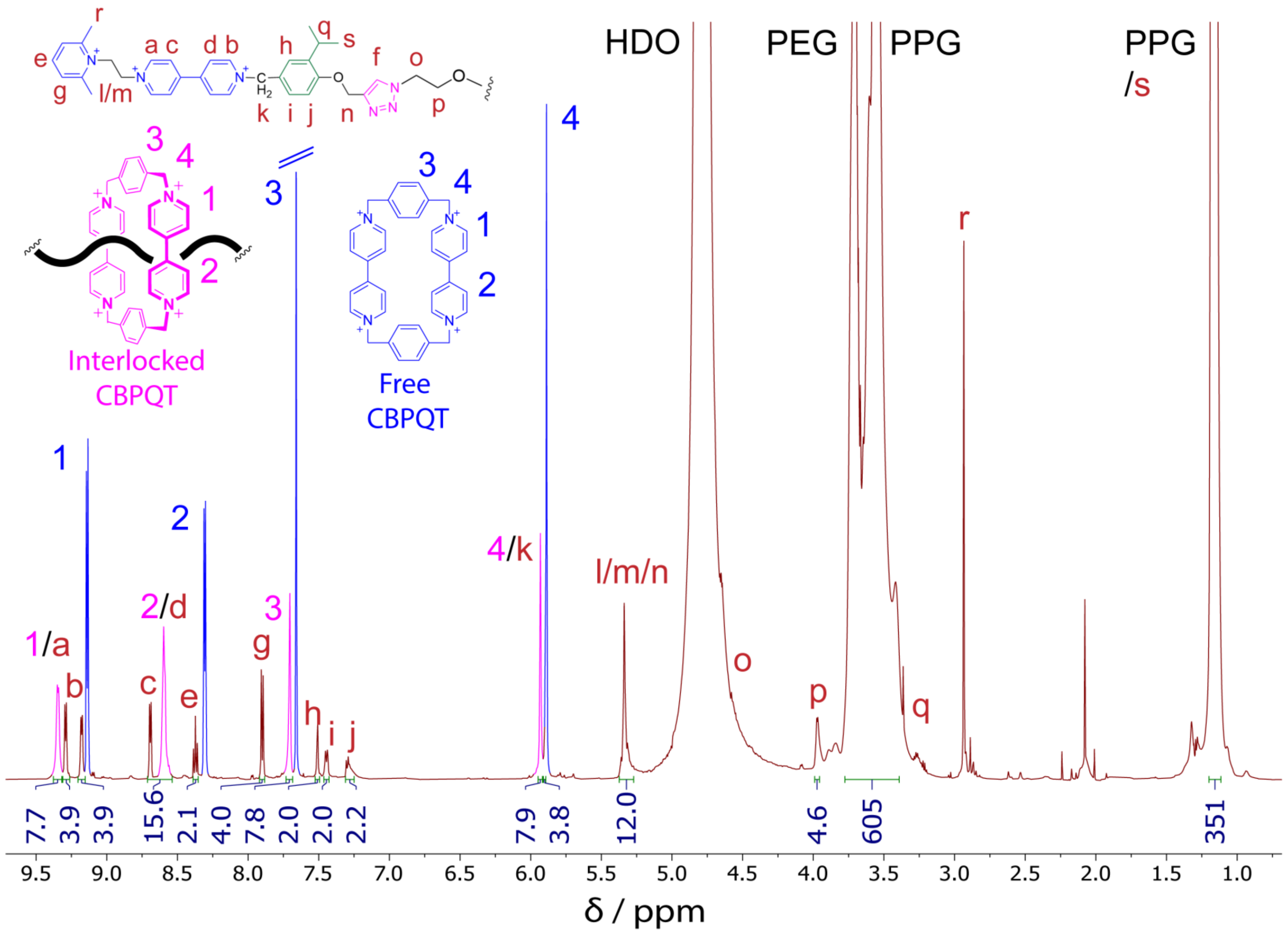

Figure S20 | Annotated ^{1}H NMR spectrum of a **Post-Pump** micellar solution of **Pluronic-BP**•6TFA and **CBPQT**•4Cl produced after slow oxidation in ambient environment (600 MHz, D_2O, 298 K). The resonance peaks corresponding to protons of interlocked **CBPQT**•4Cl are coloured magenta and those corresponding to free **CBPQT**•4Cl are coloured blue.

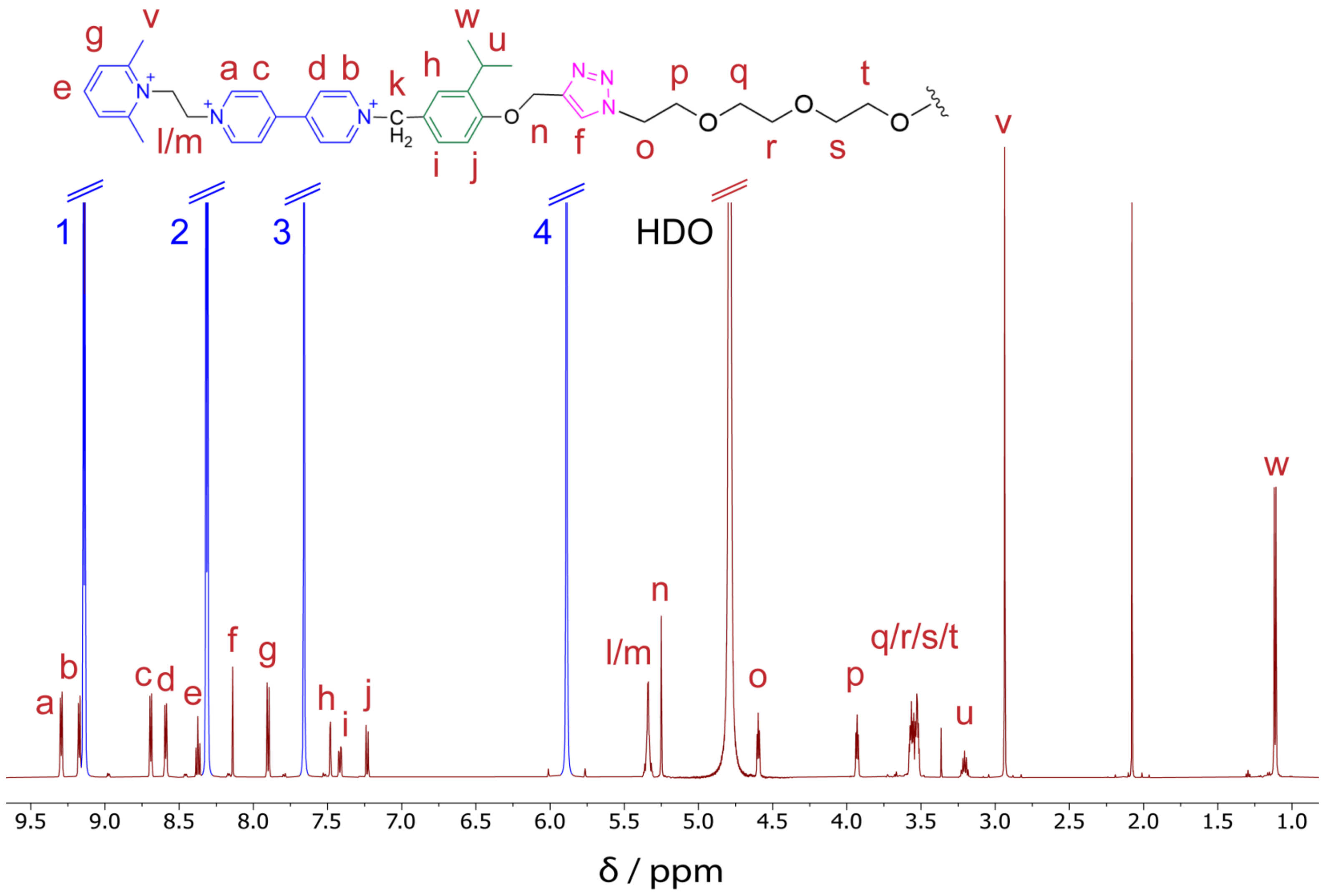

Figure S21 | Annotated ^{1}H NMR spectrum of a solution of **OligoEG-BP**•6TFA and **CBPQT**•4Cl prior to pumping (600 MHz, D_2O, 298 K). The resonance peaks corresponding to the protons of free **CBPQT**•4Cl are coloured blue.

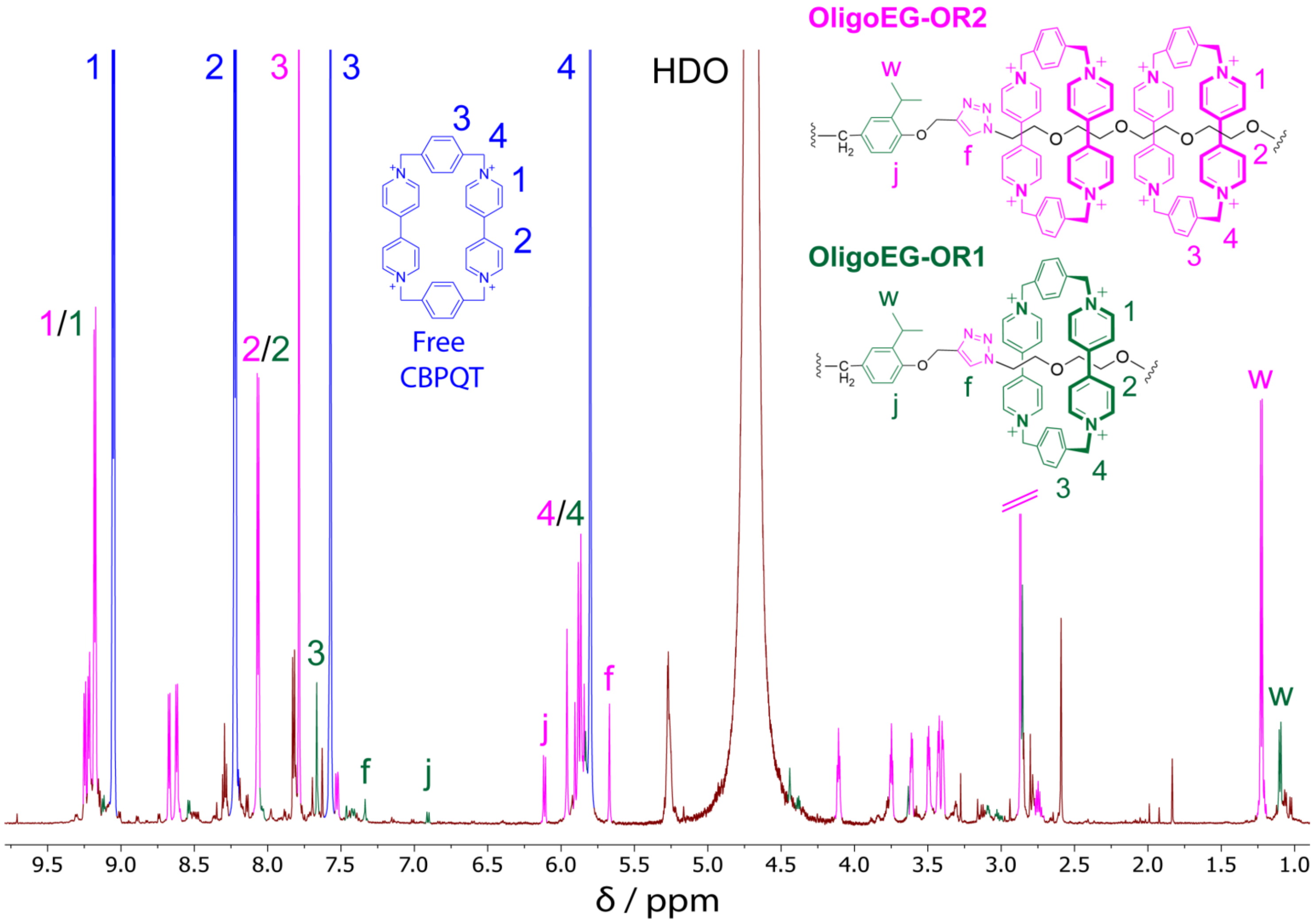

Figure S22 | Annotated ^{1}H NMR spectrum of the crude mixture of **OligoEG-OR2**, **OligoEG-OR1** and free **CBPQT**•4Cl following a pumping experiment (600 MHz, D_2O, 298 K). Certain resonance peaks corresponding to macrocycle protons of **OligoEG-OR2** (magenta), **OligoEG-OR1** (green) and free **CBPQT**•4Cl (blue) have been colour-coded. The relative concentrations of **OR2** and **OR1** can be most easily compared using resonances "f", "j" and "w".

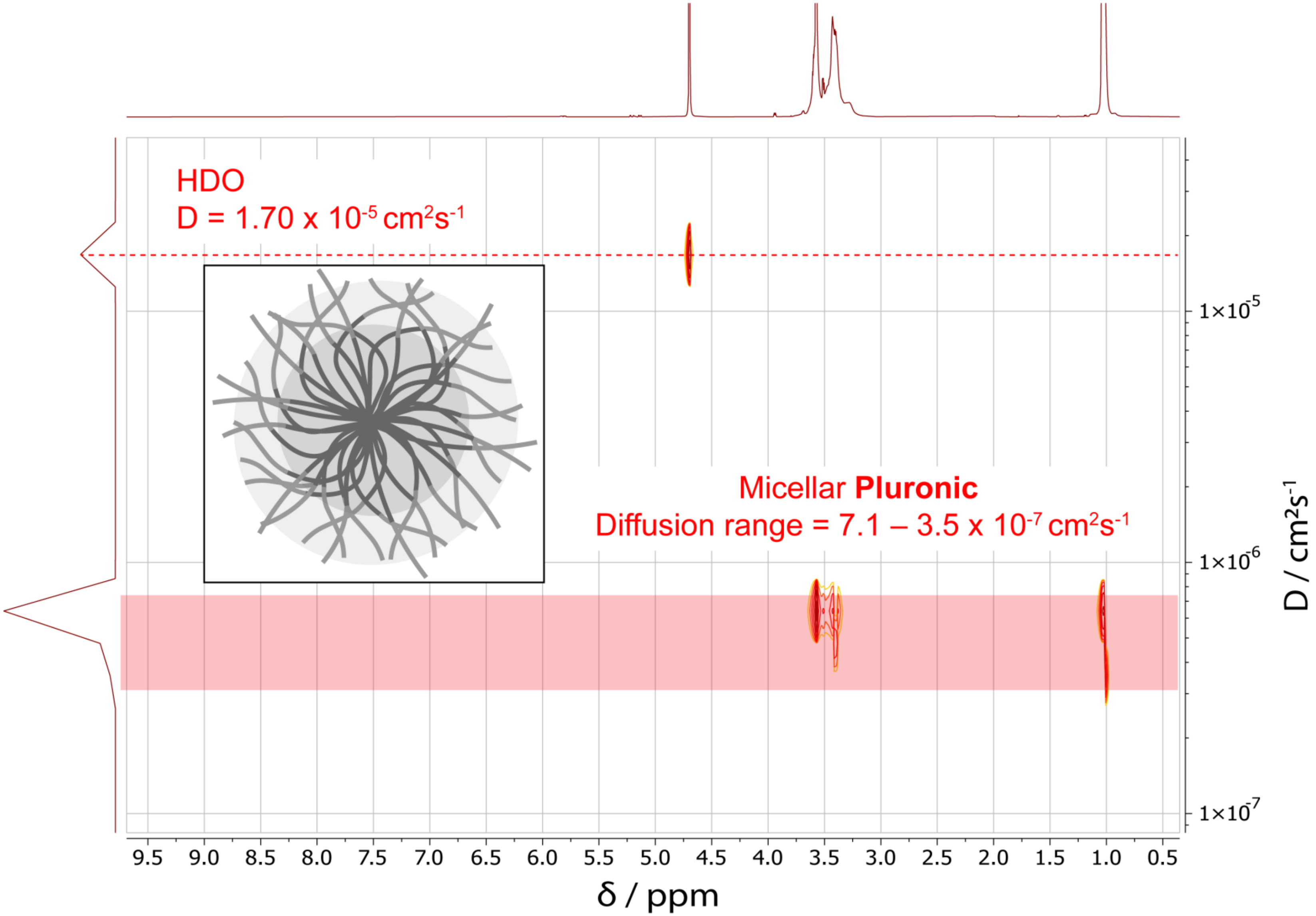

Figure S23 | DOSY NMR spectrum of a micellar solution of **Pluronic** (600 MHz, D_2O, 298 K). A graphical representation of the **Pluronic** micelle is shown.

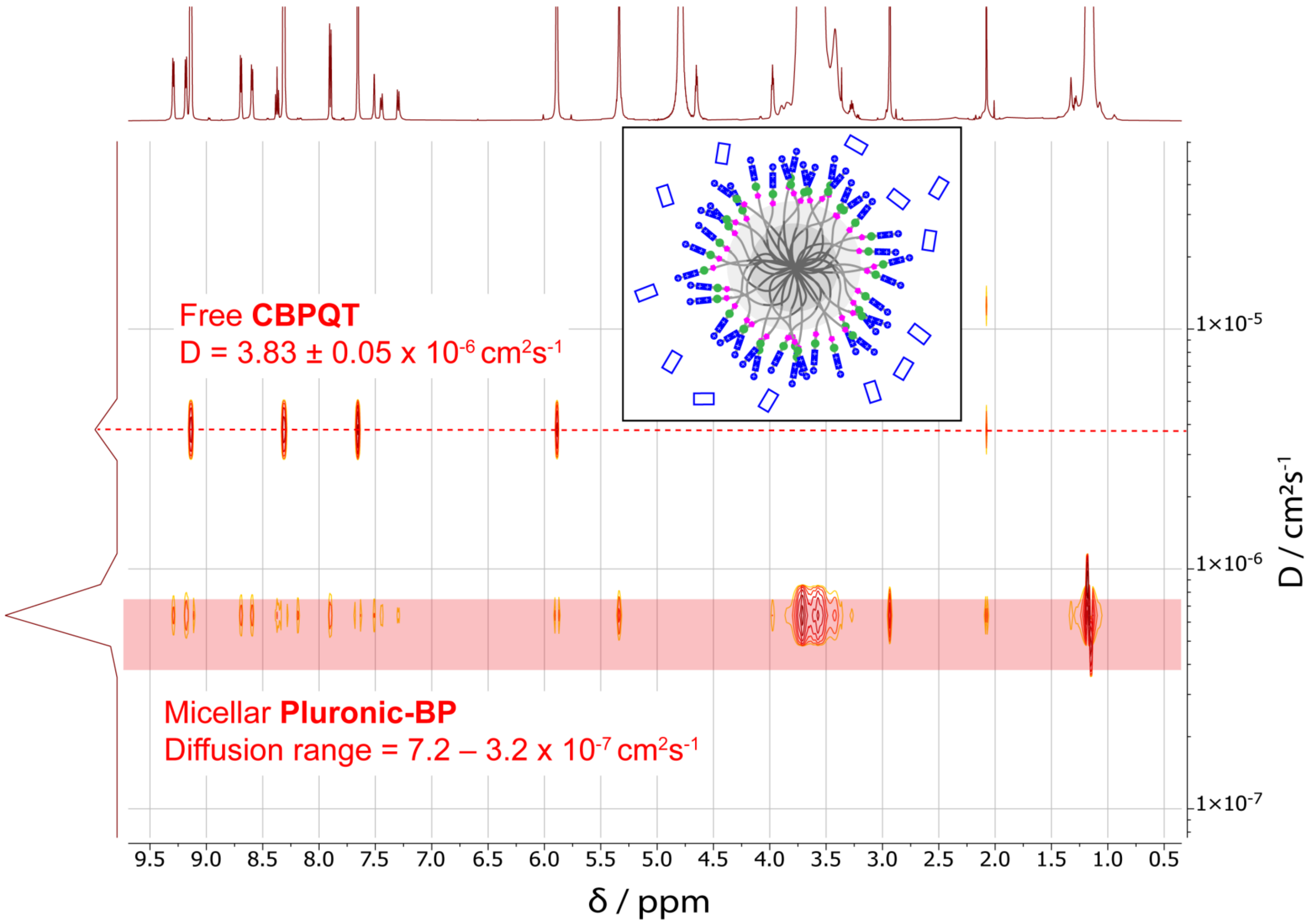

Figure S24 | DOSY NMR spectrum of a **Pre-Pump** micellar solution of **Pluronic-BP**•6TFA and **CBPQT**•4Cl (600 MHz, D_2O, 298 K). A graphical representation of a **Pluronic-BP**•6TFA micelle and free **CBPQT**•4Cl is shown.

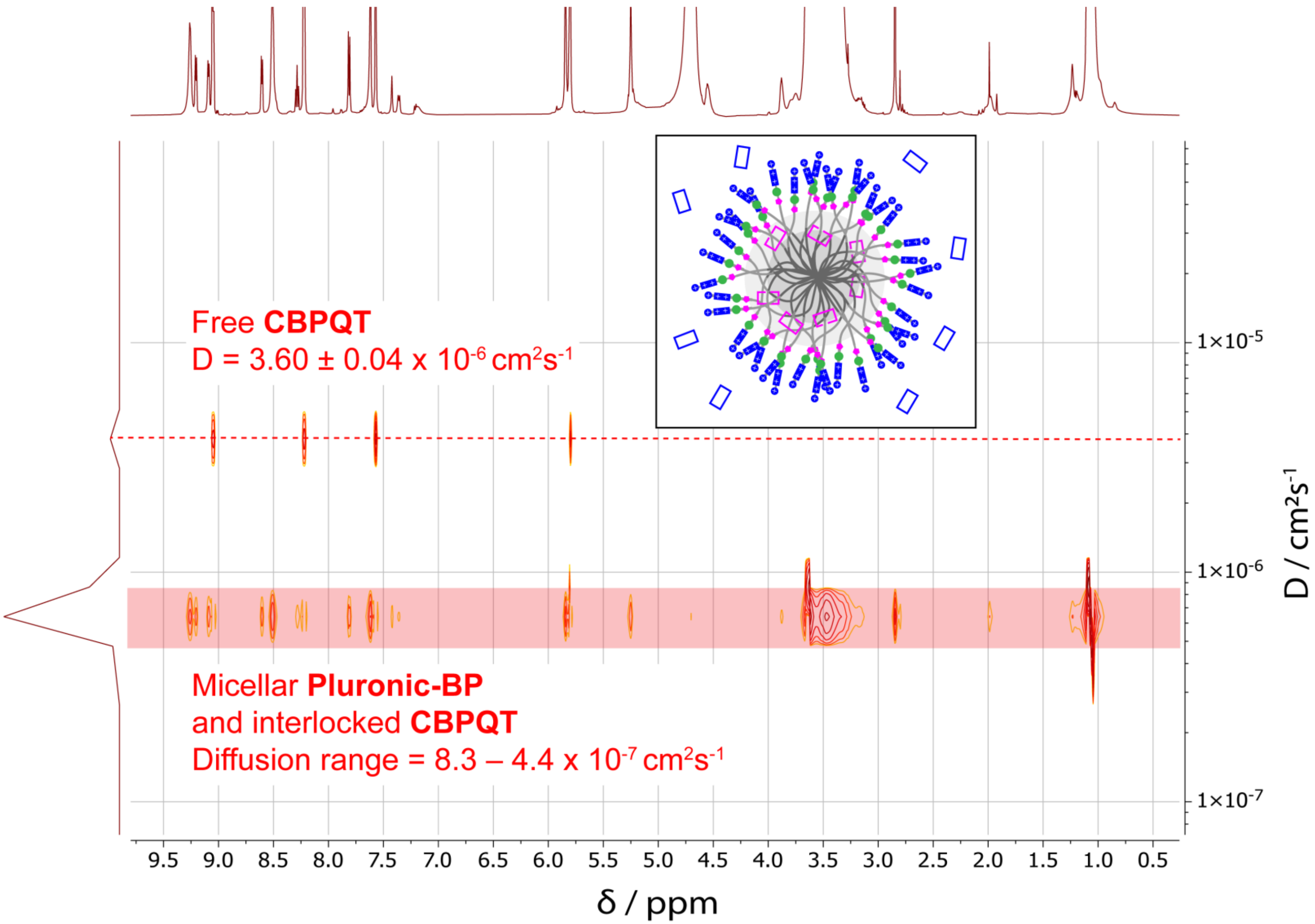

Figure S25 | DOSY NMR spectrum of a **Post-Pump** micellar solution of **Pluronic-BP**•6TFA and **CBPQT**•4Cl following oxidation by air flow (600 MHz, D_2O, 298 K). A graphical representation of a **Pluronic-BP**•6TFA micelle containing interlocked **CBPQT**, surrounded by free **CBPQT**•4Cl, is shown.

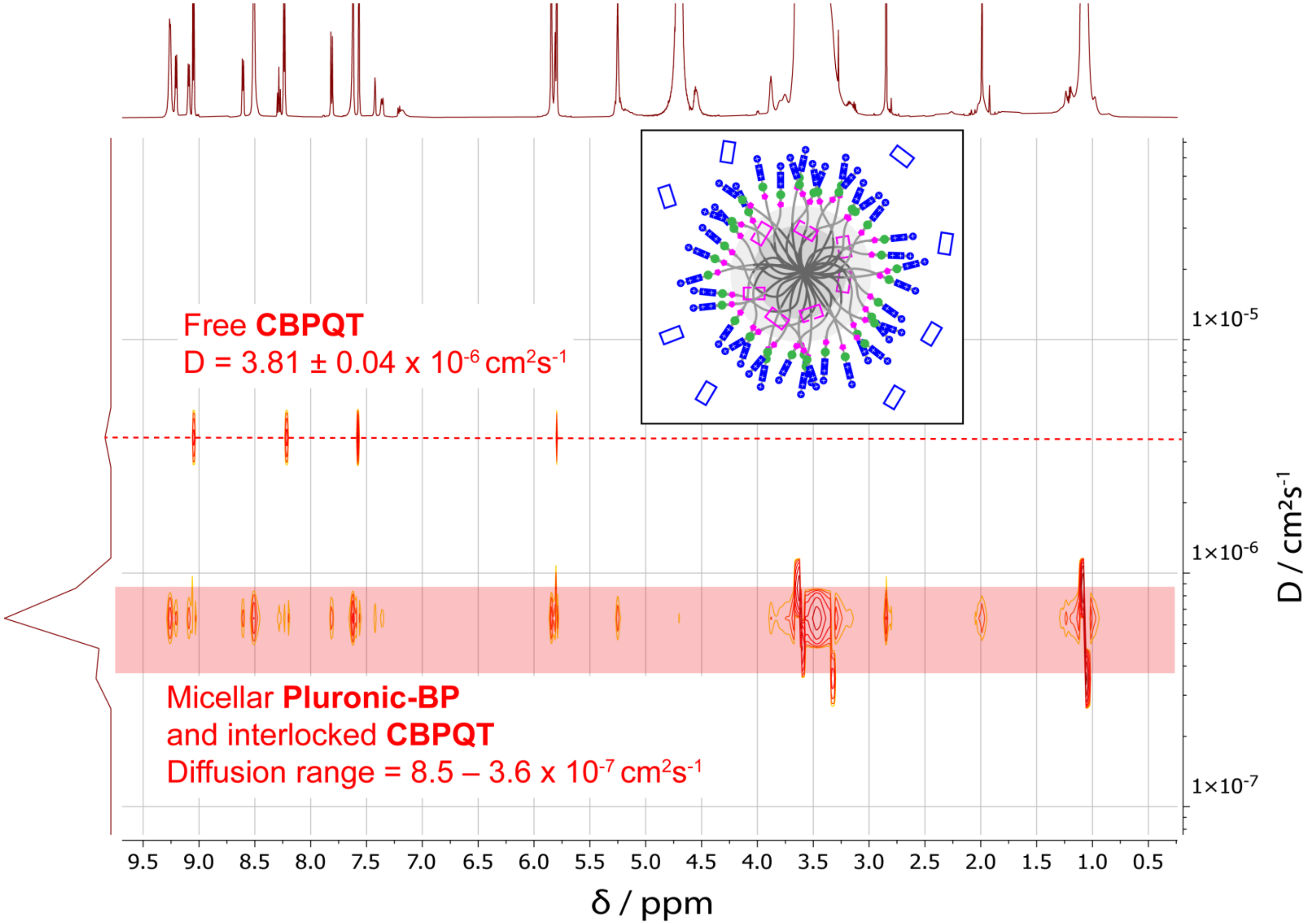

Figure S26 | DOSY NMR spectrum of a **Post-Pump** micellar solution of **Pluronic-BP**•6TFA and **CBPQT**•4Cl following oxidation with elemental I_2 (600 MHz, D_2O, 298 K). A graphical representation of a **Pluronic-BP**•6TFA micelle containing interlocked **CBPQT**, surrounded by free **CBPQT**•4Cl, is shown.

Manual DOSY Diffusion Calculations

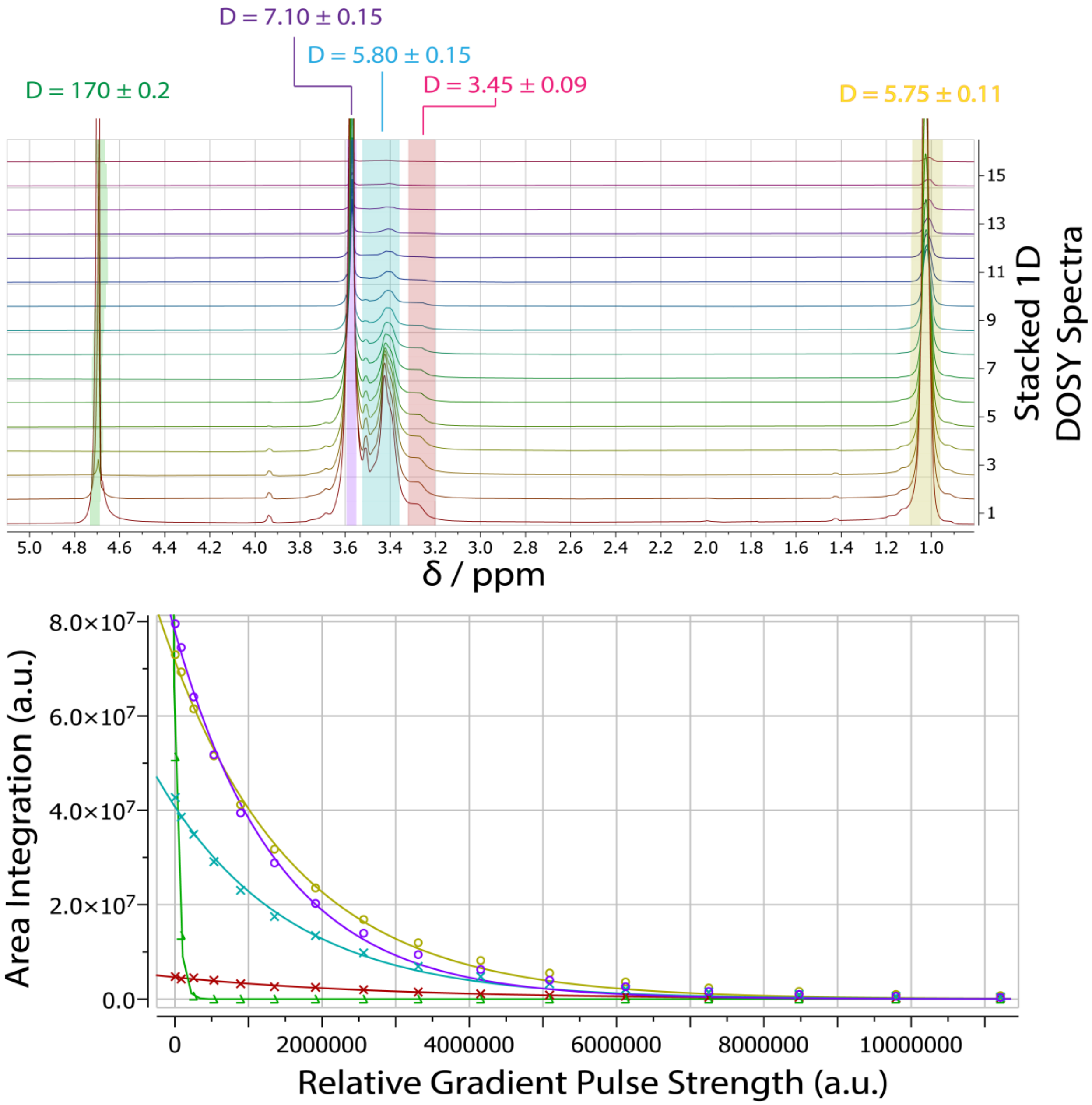

Figure S27 | A stack of 1D ^1H DOSY NMR spectra for a sample of **Pluronic** in D_2O (top) and a fitting graph (bottom). On the 1D DOSY stack, the resonances of interest have been highlighted in colour. The resonance peak integral attenuation was plotted and a monoexponential function was fitted to the data. From this fit, the average diffusion constants corresponding to the resonance peaks were measured. The values are displayed above the NMR stack. This method was applied for the calculation of all diffusion constants.

Sample	Resonance Peak	Average Diffusion Constant ($\times 10^{-7}$ cm^2/s)
**Pluronic-BP**•6TFA in CD_3OD (nonmicellar)	All resonance peaks	16.6 ± 0.4
Micellar **Pluronic**	PEG $-CH_2-$	7.14 ± 0.15
	PPG $-CH_2-$ and -CH-	5.80 ± 0.15
	PPG core resonances	3.45 ± 0.09
	PPG $-CH_3$	5.75 ± 0.11
**Pre-Pump** Micellar Pluronic-BP & free CBPQT	Free CBPQT (avg of 3)	38.3 ± 0.50
	Molecular Pump (avg of 5)	7.21 ± 0.45
	PEG $-CH_2-$	7.03 ± 0.11
	PPG $-CH_2-$ and -CH-	4.90 ± 0.11
	PPG core resonances	3.21 ± 0.11
	PPG $-CH_3$	5.08 ± 0.11
**Post-Pump** *Air-oxidized* Micellar Pluronic-BP, interlocked CBPQT & free CBPQT	Free CBPQT (avg of 3)	36.0 ± 0.37
	Interlocked CBPQT (avg of 4)	7.56 ± 0.21
	Molecular Pump (avg of 5)	8.29 ± 0.37
	PEG $-CH_2-$	7.73 ± 0.17
	PPG $-CH_2-$ and -CH-	6.58 ± 0.13
	PPG core resonances	4.13 ± 0.13
	PPG $-CH_3$	6.51 ± 0.14
**Post-Pump** *I_2-oxidized* Micellar Pluronic-BP, interlocked CBPQT & free CBPQT	Free CBPQT (avg of 3)	38.1 ± 0.41
	Interlocked CBPQT (avg of 4)	7.54 ± 0.32
	Molecular Pump (avg of 5)	8.47 ± 0.46
	PEG $-CH_2-$	7.95 ± 0.18
	PPG $-CH_2-$ and -CH-	6.08 ± 0.12
	PPG core resonances	3.60 ± 0.09
	PPG $-CH_3$	5.99 ± 0.13

Table S2 | Diffusion constants calculated for constituents of nonmicellar and micellar solutions. The Stejskal-Tanner equation[18] was used to relate attenuation of resonance peak integral to average diffusion constant. A monoexponential fit was used to calculate diffusion constants, which results in an averaged value for each resonance. For free and interlocked $CBPQT^{4+}$ and the molecular pump, which afford multiple distinct proton resonances, multiple diffusion constant measurements were made and the average is reported. The reported error is the higher of either (1)

the quadratic error of the fit or (2) the standard deviation for values which are an average of multiple measurements.

Measurement of T_2 Values

**Component**	**Resonance Peak** (assigned letter)	**T_2** (ms)
Polymer chain	PPG core resonances	56 ± 4
	PPG $-CH_3$	132 ± 12
	PPG $-CH_2-$ and -CH-	115 ± 8
	PEG $-CH_2-$	367 ± 16
	External PEG $-CH_2-$ ("p")	336 ± 46
Molecular Pump	"l/m"	261 ± 5
	Lutidinium $-CH_3$ ("r")	676 ± 29
	BIPY ("a")	437 ± 24
	BIPY ("b")	446 ± 50
	BIPY ("c")	453 ± 16
	BIPY ("d")	485 ± 11

Table S3 | T_2 values calculated for constituents of a **Pre-Pump** micellar solution of **Pluronic-BP**•6TFA and **CBPQT**•4Cl. Values were measured by applying a monoexponential fit to the attenuating integration values of resonance peaks in ^{1}H NMR spectra recorded using the cpmgpr pulse sequence. The reported error is the quadratic error of the fit.

Stokes-Einstein Equation

The Stokes-Einstein relation is given by:

$$D = \frac{k_b T}{6\pi\eta r} \tag{1}$$

Where D is diffusion constant, k_b is the Boltzmann constant, T is the temperature (The thermal energy, K_bT, is 4.1164 x 10^{-21} J at 25 °C), η is the absolute viscosity (1.096 x 10^{-9} J·s·cm^{-3} for D_2O at 25 °C)[19] and r is the radius of the spherical particle. Using the diffusion constant measured for the core resonance in the **Pre-Pump** solution, a hydrodynamic diameter of 12.4 nm is calculated for the micelles.

6. Size Exclusion Chromatography

Size-exclusion chromatography of **Pluronic** was performed in triplicate in THF. The polydispersity of the primary peak was determined to be 1.03 ± 3%. A dn/dc value for **Pluronic** of 0.0805 was input to ASTRA software to make this calculation. Only the primary peak eluting at 15.9 min was used to determine the polydispersity. The shoulder peak eluting at 17.1 min is attributed to low molecular weight ether-terminated polymeric impurities and is excluded from the calculation of polydispersity. Since those impurities were removed after the attachment of molecular pumps, the polydispersity value of 1.03 can be considered representative of the polymer backbones of **Pluronic-BP**•6TFA.

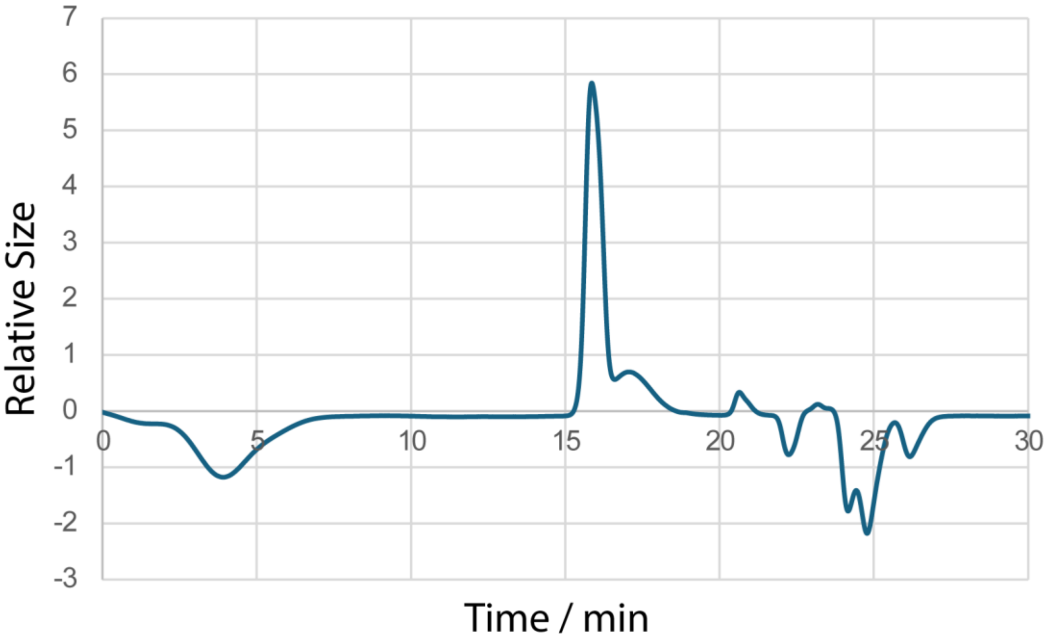

Figure S28 | Elution profile for **Pluronic** dissolved in THF at 3 mg/mL concentration. Elution was monitored by differential refractometry. The primary peak of **Pluronic** elutes at 15.9 min. The shoulder peak at 17.1 min is attributed to low molecular weight polymeric impurities.

SEC profiles of **Pluronic-BP**•6TFA and **Pluronic** were compared in DMF at 1 mg/mL (Figure S27). DMF was chosen as the solvent for this comparison because of its ability to fully dissolve completely both the polymeric chains and the terminal molecular pump cassettes. Note that the molecular pump cassettes are insoluble in THF. The primary peak of **Pluronic-BP**•6TFA elutes earlier than that of **Pluronic**, consistent with the larger mass of the end-capped polymer. The data also indicate the absence of low molecular weight impurities in **Pluronic-BP**•6TFA.

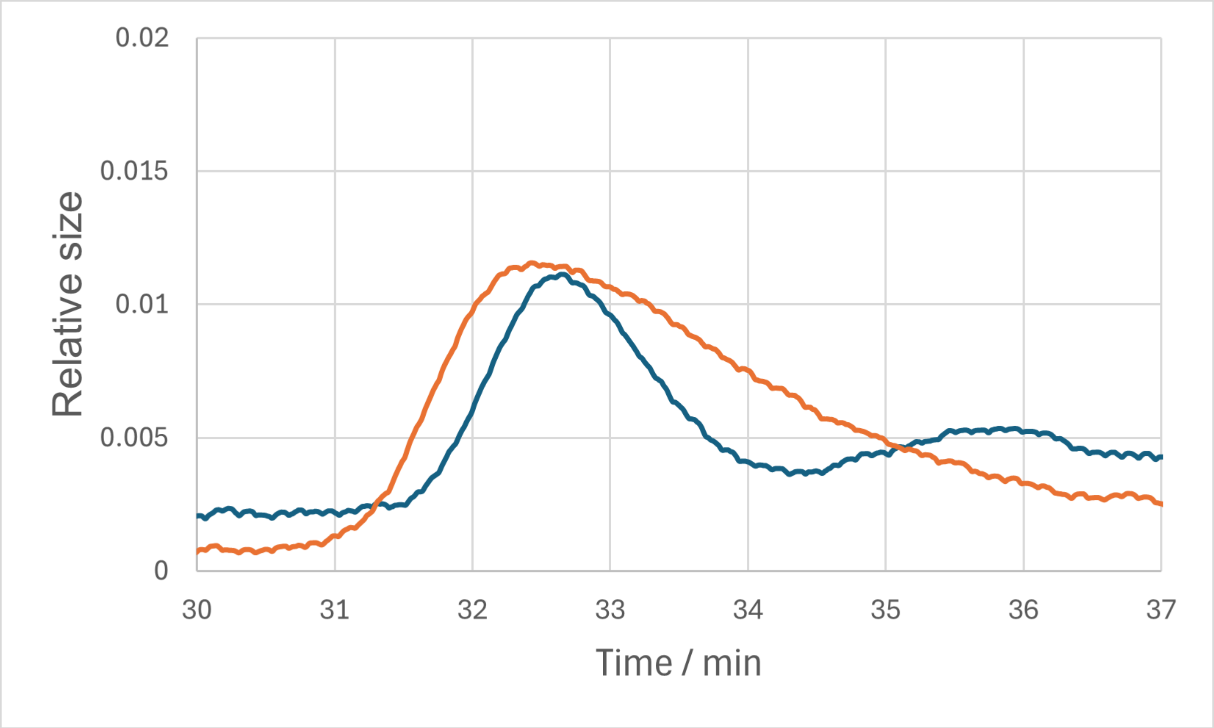

Figure S29 | Elution profile for separate SEC runs of **Pluronic** (blue trace) and **Pluronic-BP**•6TFA (orange trace) dissolved at 1 mg/mL in DMF. Elution was monitored by differential refractometry. The earlier elution onset observed for **Pluronic-BP**•6TFA relative to the **Pluronic** starting material is attributed to the larger size of the end-capped polymer.

7 Dynamic Light Scattering and Zeta Potential

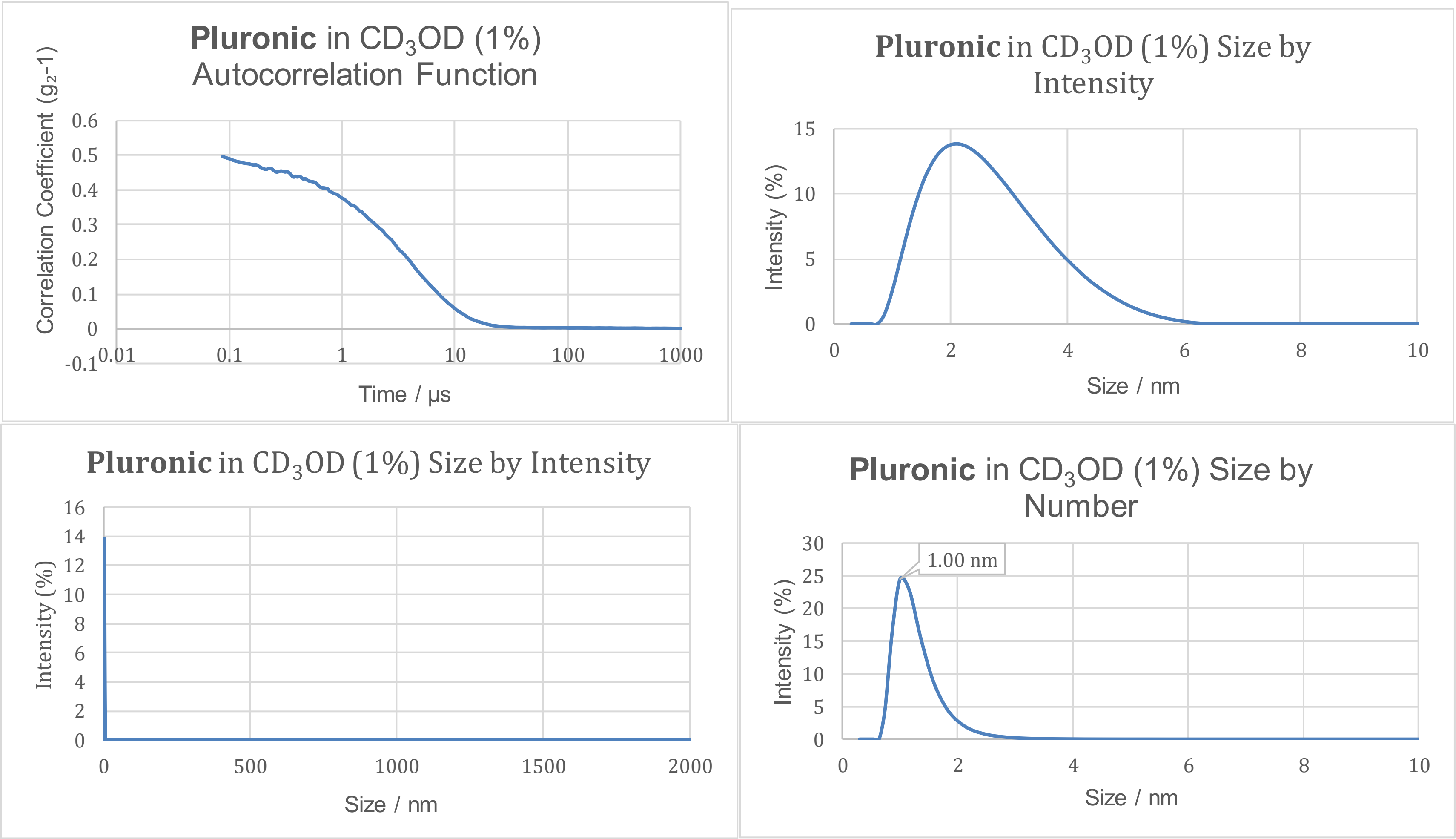

Figure S30 | DLS results for **Pluronic** dissolved in deuterated methanol (1% m/v). An absence of **Pluronic** micelles is observed in this solvent.

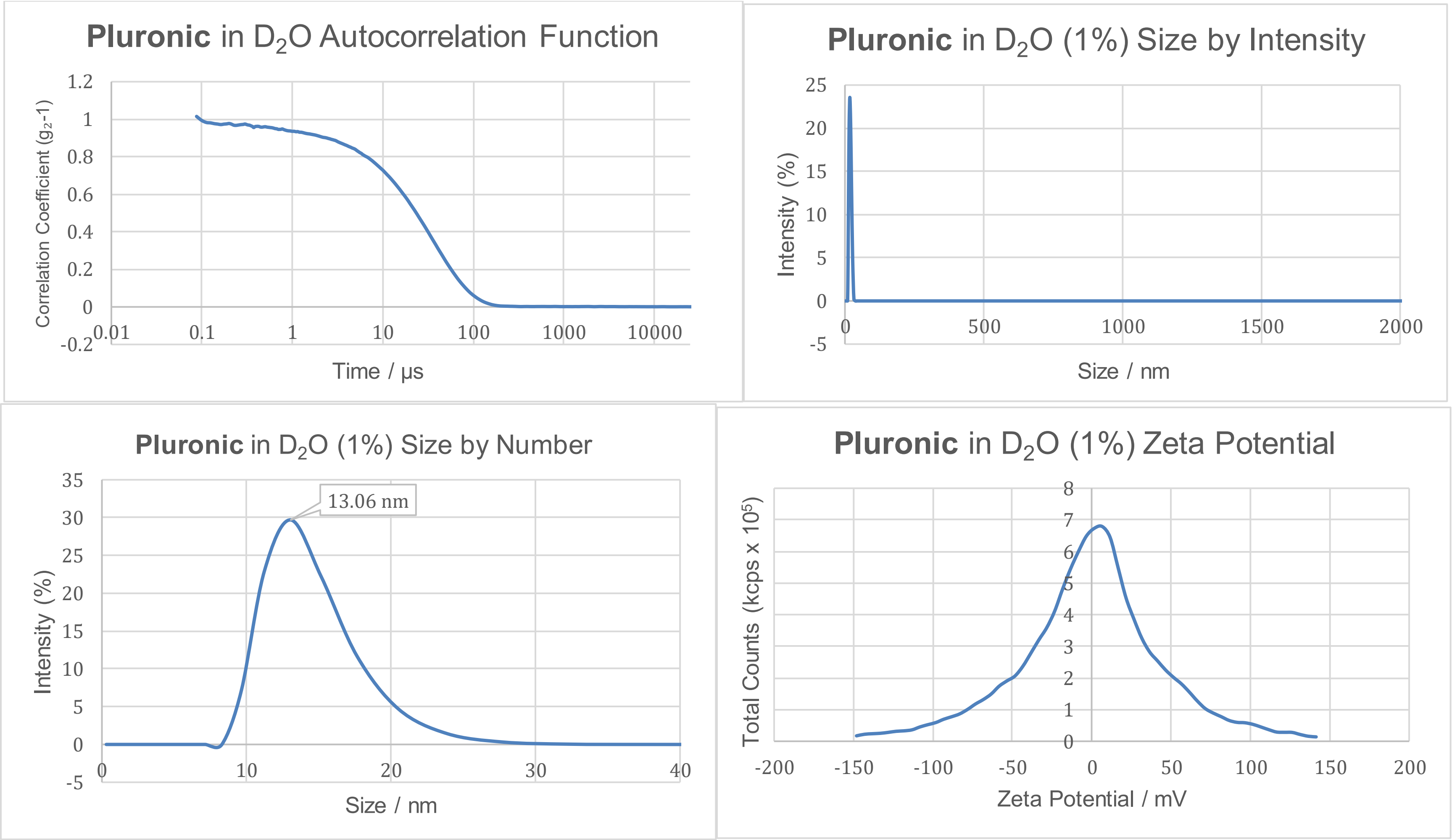

Figure S31 | DLS and zeta potential results of a solution of **Pluronic** in D_2O (1% m/v). The mean particle size is 14.0 nm and the mean zeta potential is –1.1 mV.

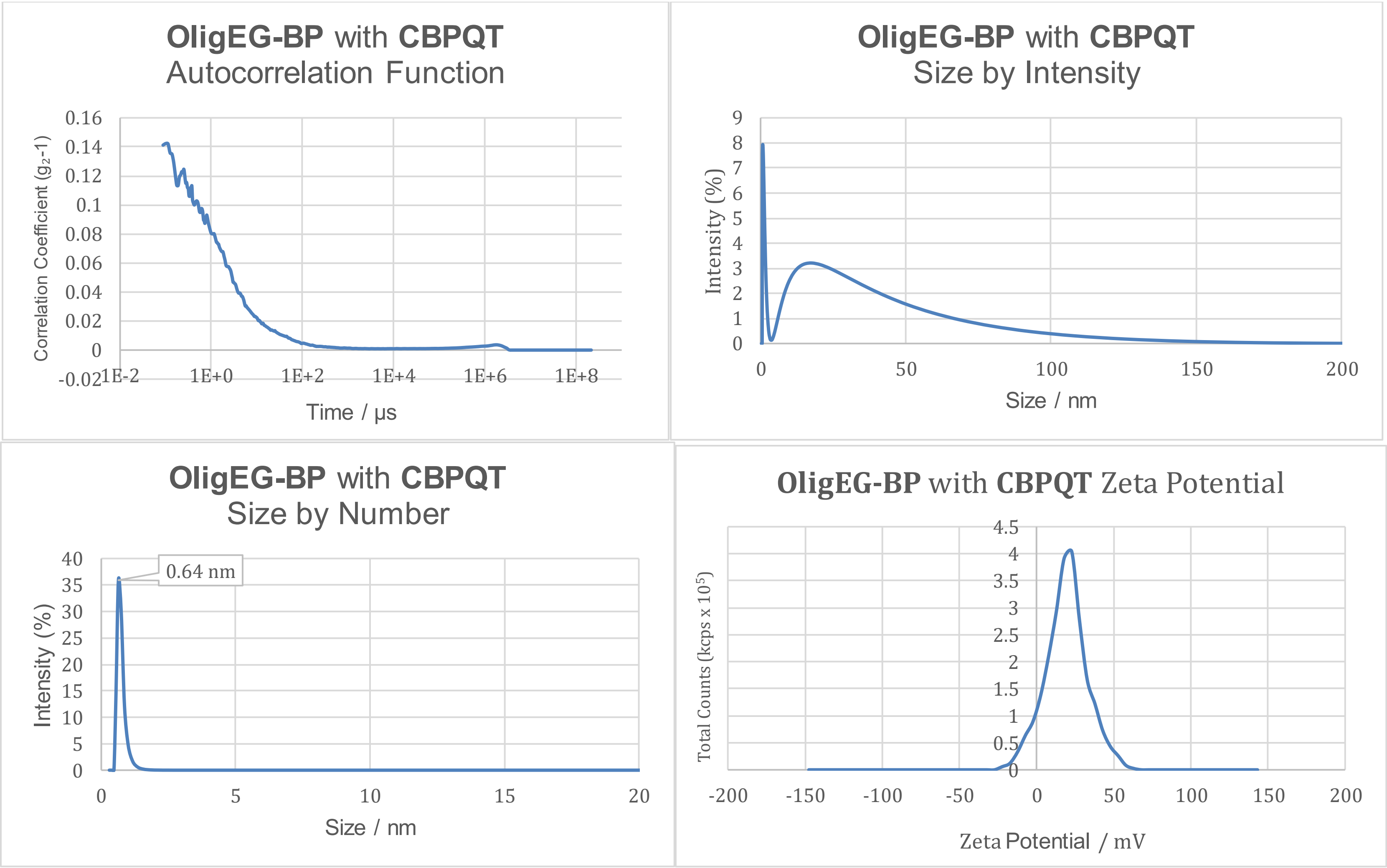

Figure S32 | DLS and zeta potential results for the D_2O solution of **OligoEG-BP**•6TFA (1 mg/mL) and **CBPQT**•4Cl (1.5 mg/mL). An absence of aggregates is observed. The mean zeta potential is +19.3 mV.

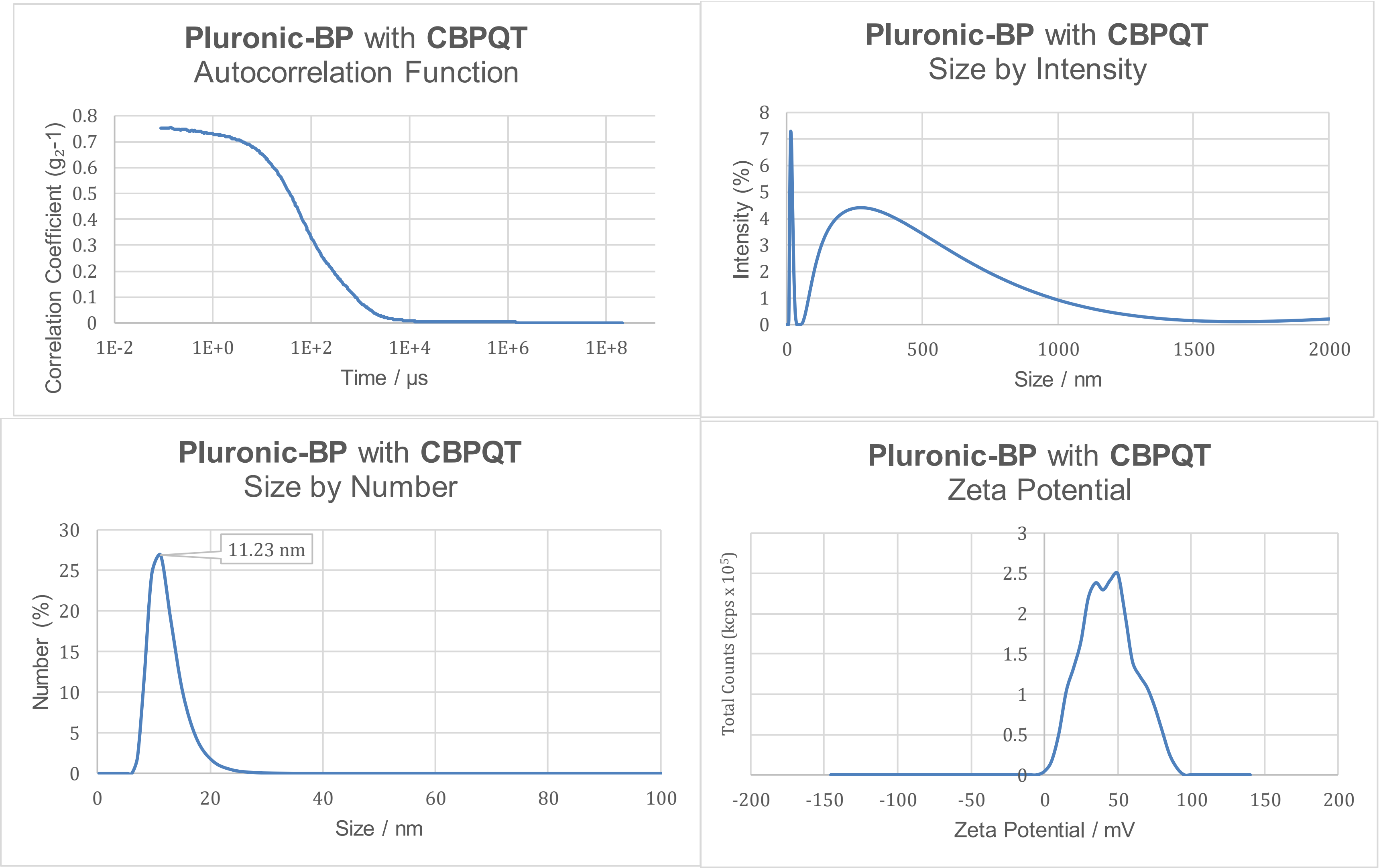

Figure S33 | DLS and zeta potential of a D_2O solution of the **Pre-Pump** micellar solution of **Pluronic-BP**•6TFA (5.0 mg/mL) and **CBPQT**•4Cl (1.5 mg/mL). The mean zeta potential is +43.8 mV.

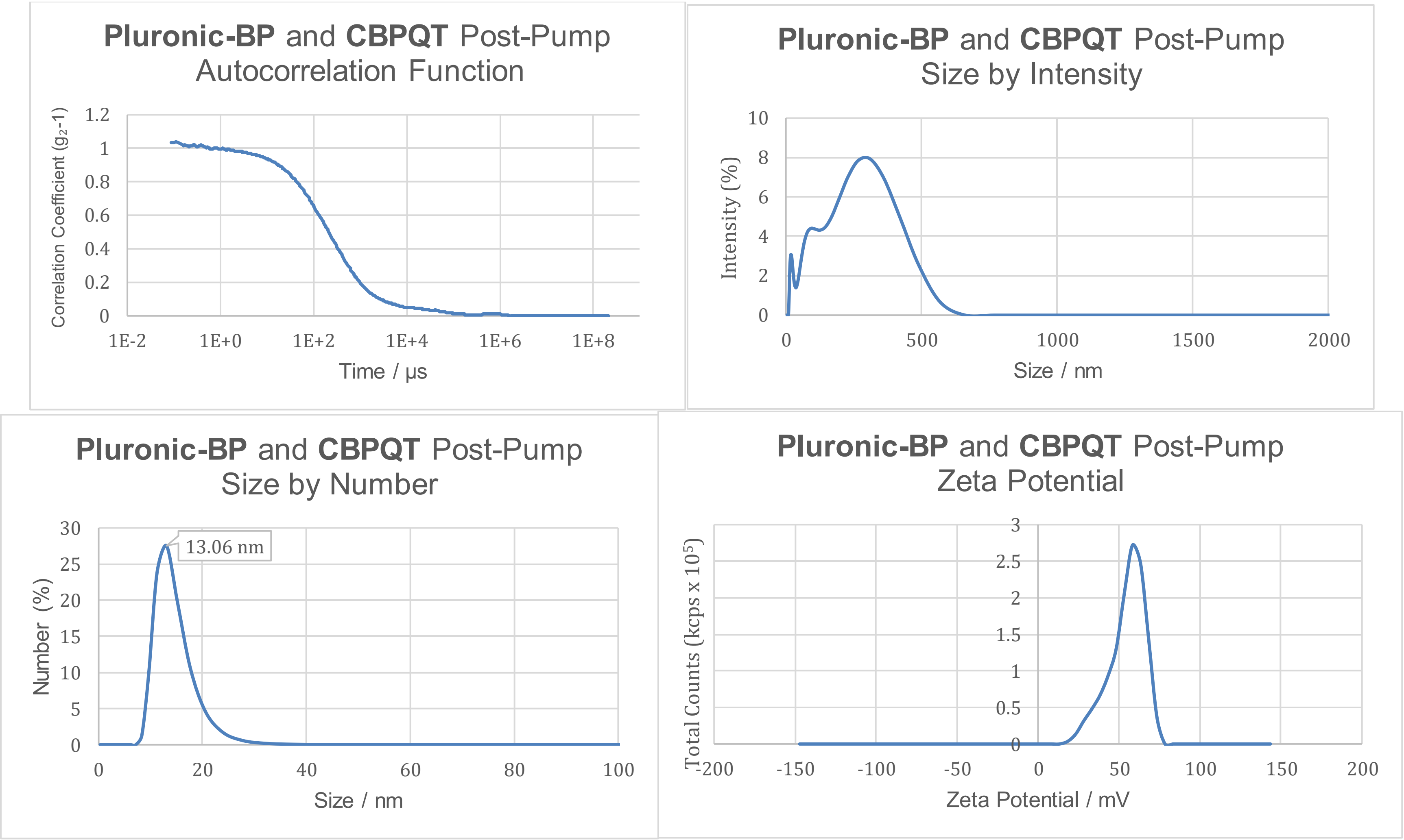

Figure S34 | DLS and zeta potential of the **Post-Pump** solution of micelles oxidized through air flow. The mean zeta potential is +54.7 mV**.**

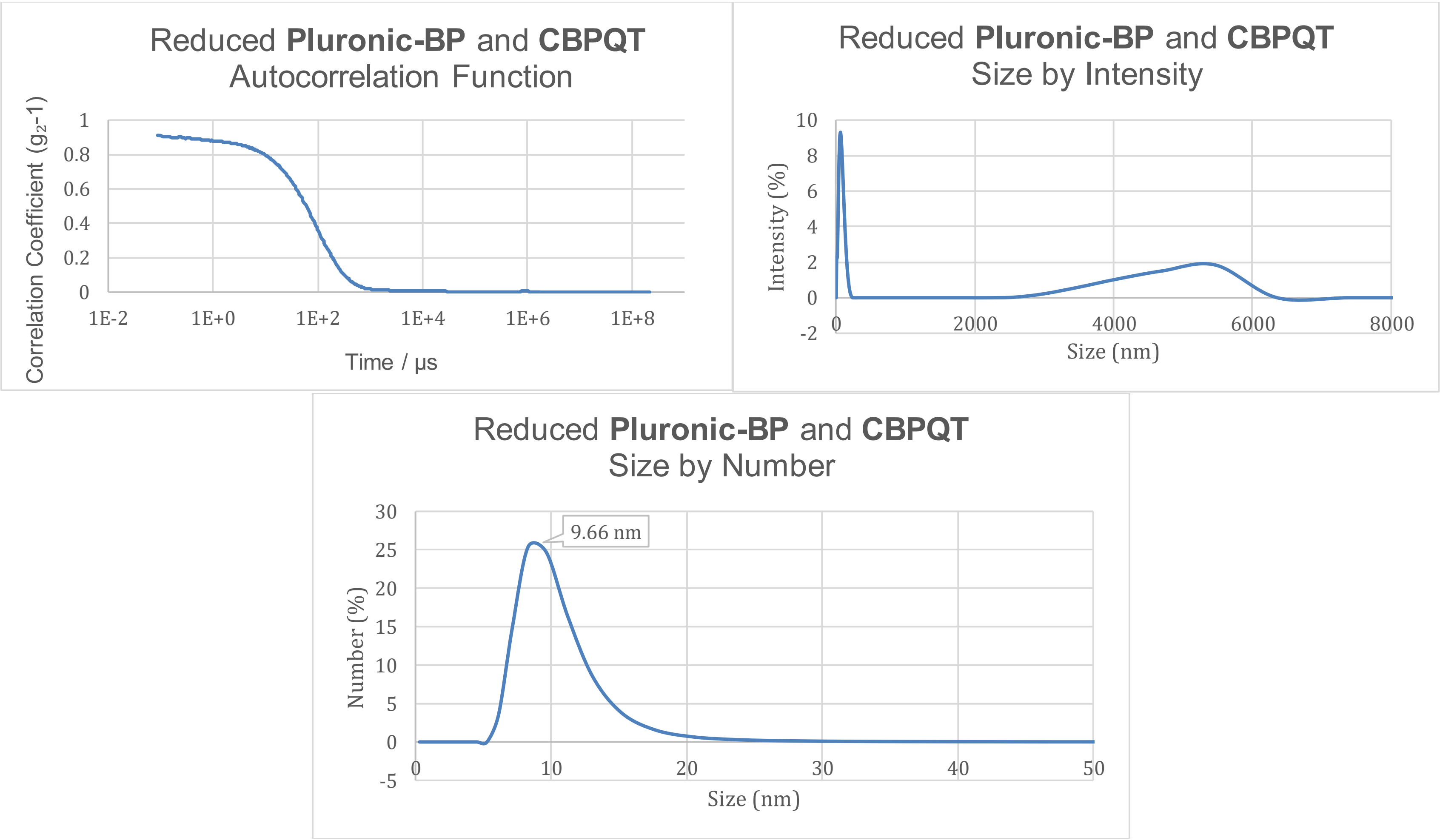

Figure S35 | DLS data for the **Pre-Pump** micellar solution after reduction with zinc powder. The solution was filtered to remove undissolved zinc powder prior to measurement. The data provides a snapshot of the system between the **Pre-Pump** and **Post-Pump** states, during the phase in which rings are captured, though not yet pumped, by the molecular pump cassettes.

8 UV-Vis-NIR Spectroscopy

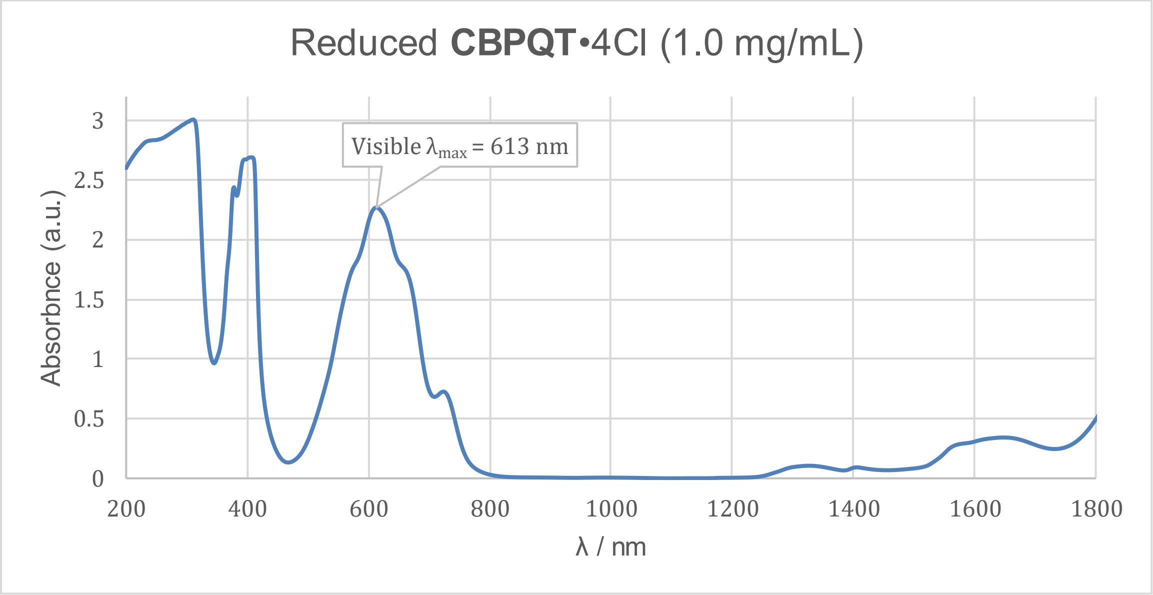

Figure S36 | Annotated UV-Vis-NIR spectrum of a 1.0 mg/mL solution of **CBPQT**•4Cl in D_2O reduced using zinc dust

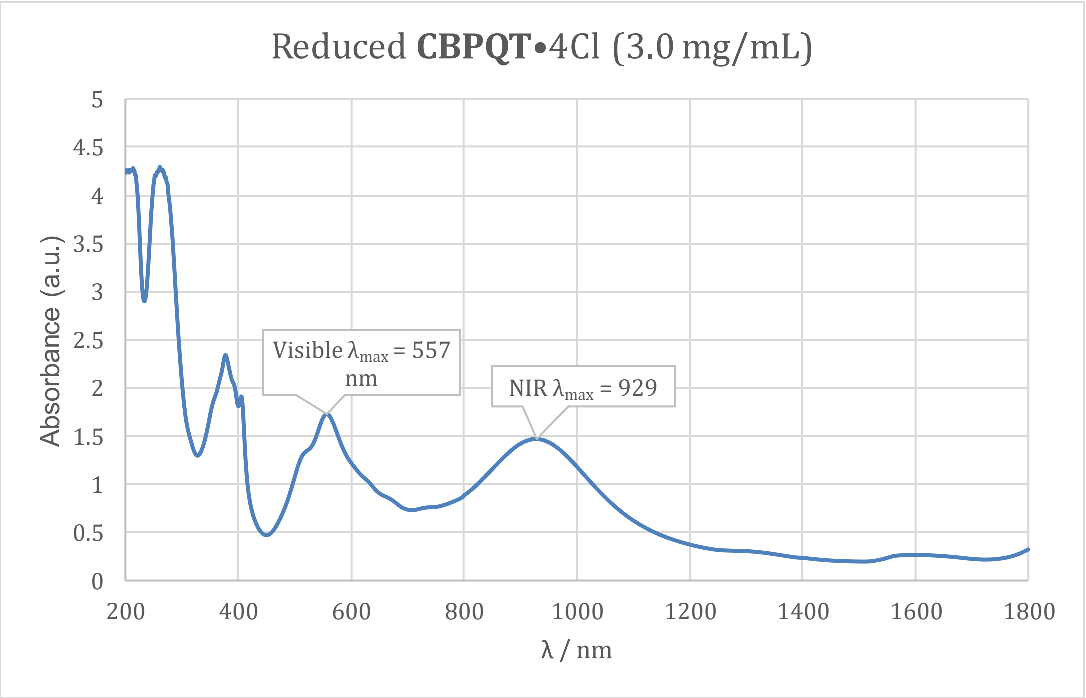

Figure S37 | Annotated UV-Vis-NIR spectrum of a 3.0 mg/mL solution of **CBPQT**•4Cl in D_2O reduced using zinc dust

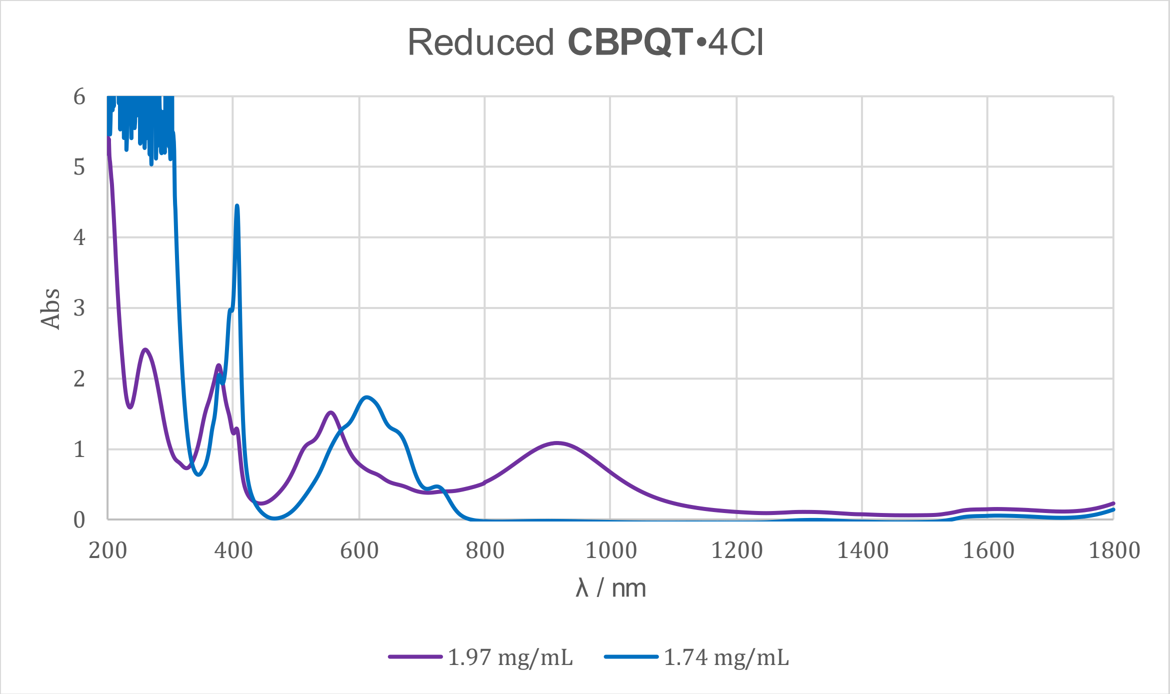

Figure S38 | UV-Vis-NIR spectra of a 1.74 mg/mL (blue trace) and 1.97 mg/mL (purple trace) solution of **CBPQT**•4Cl in D_2O reduced using zinc dust. The critical aggregation concentration in D_2O occurs somewhere in the range 1.74 – 1.97 mg/mL of **CBPQT**•4Cl. See Section 4 for a discussion of this graph.

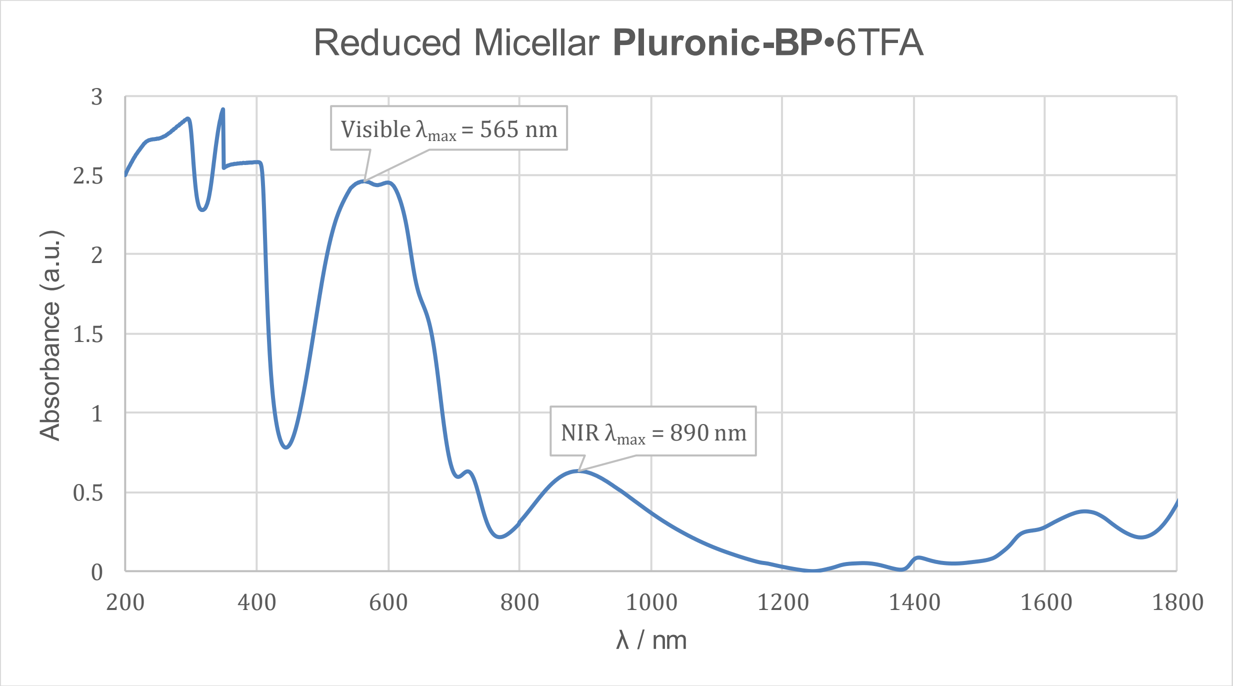

Figure S39 | Annotated UV-Vis-NIR spectrum of a solution of **Pluronic-BP**•6TFA reduced using zinc dust

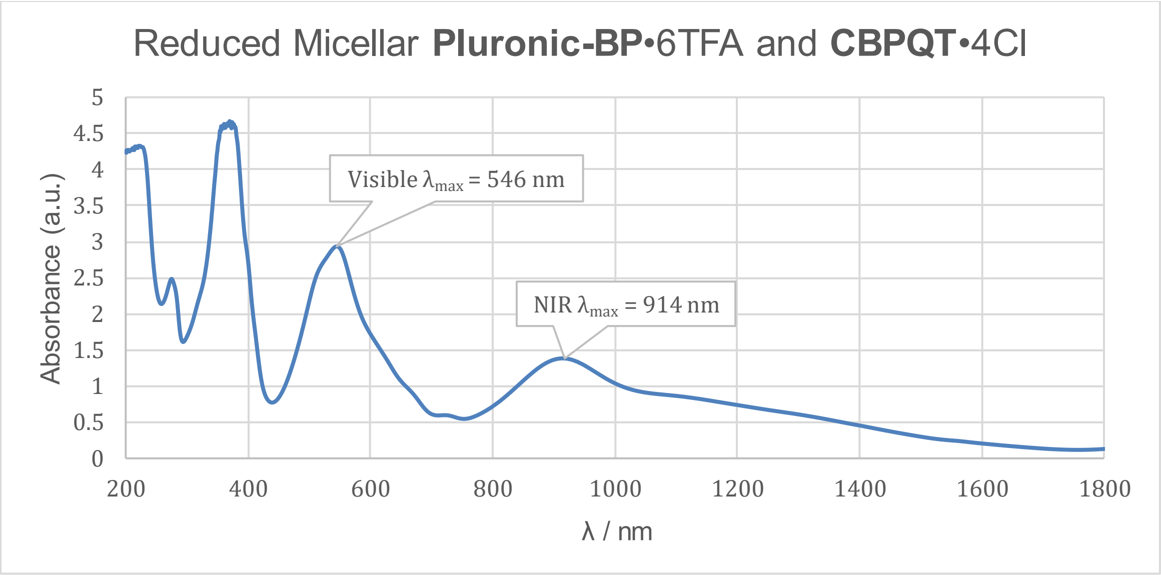

Figure S40 | Annotated UV-Vis-NIR spectrum of a micellar solution of **Pluronic-BP**•6TFA and **CBPQT**•4Cl reduced using zinc dust

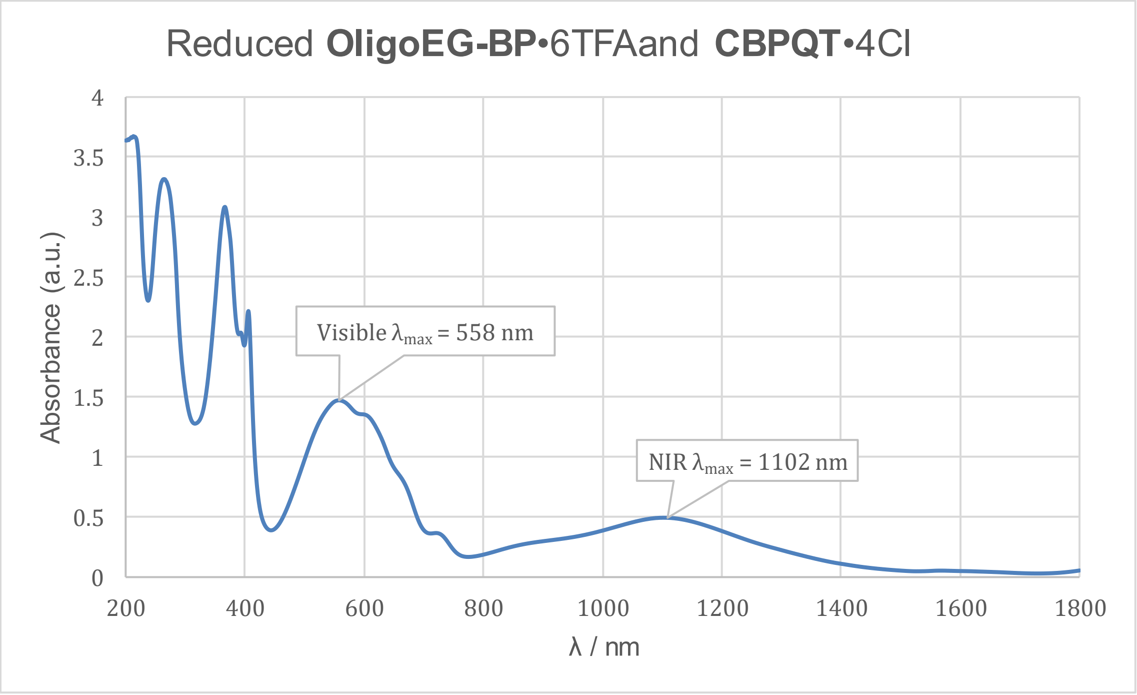

Figure S41 | Annotated UV-Vis-NIR spectrum of a solution of **OligoEG-BP**•6TFA and **CBPQT**•4Cl reduced using zinc dust

9 Mass Spectrometry

MALDI Mass Spectrometry

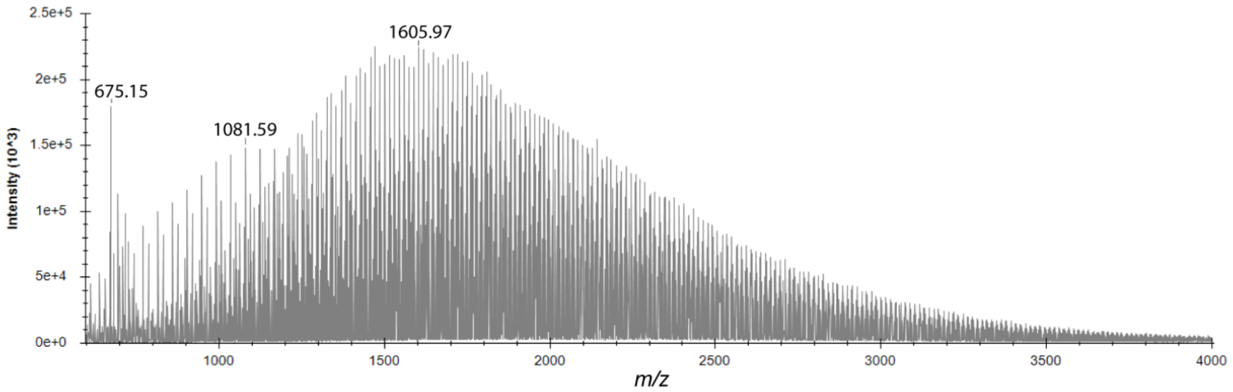

Figure S42 | MALDI Mass spectrum of **Pluronic** in the *m/z* 500 – 4000 range showing mass distributions for the ether-terminated impurities.

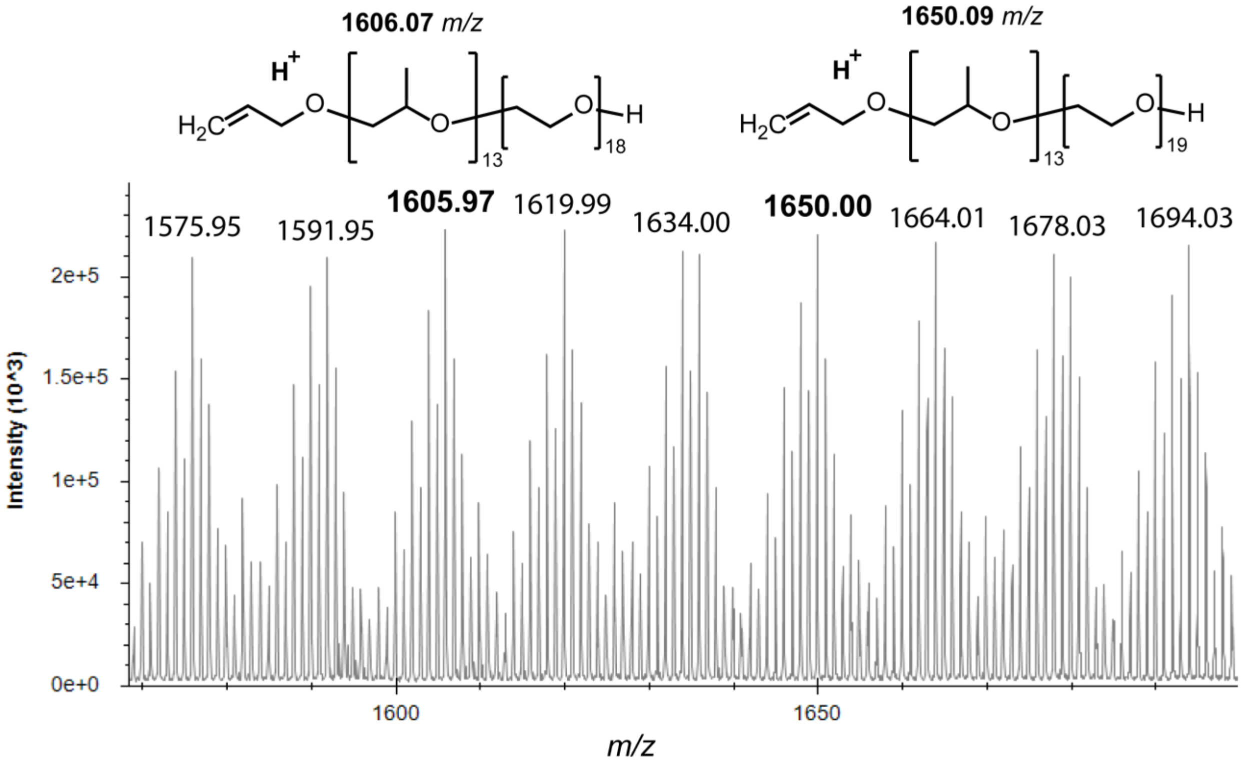

Figure S43 | MALDI Mass spectrum of **Pluronic** magnified to the region *m/z* 1570 – 1700. Two assignments are proposed for peaks at *m/z* 1606 and 1650.

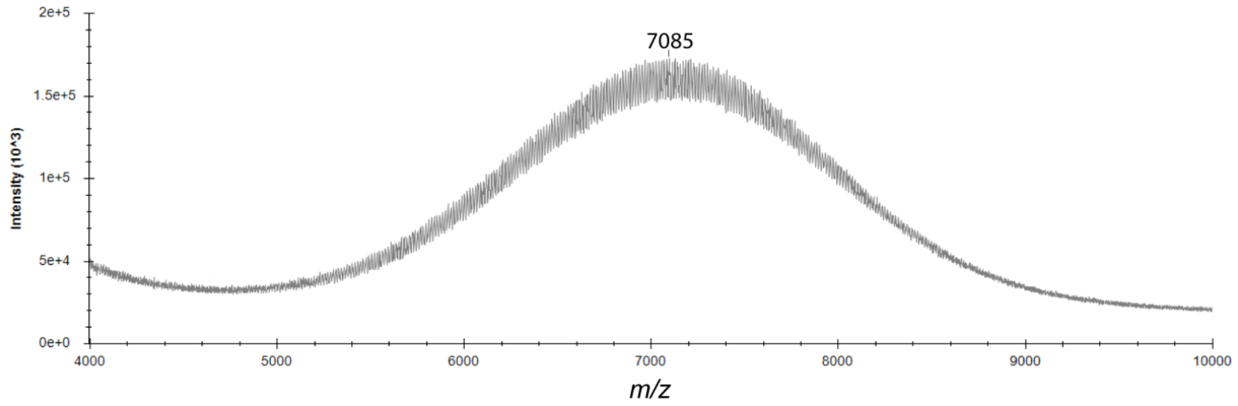

Figure S44 | MALDI Mass spectrum of **Pluronic** in the *m/z* 4 – 10K range showing the mass distribution for the triblock copolymer that is the principal component of **Pluronic.**

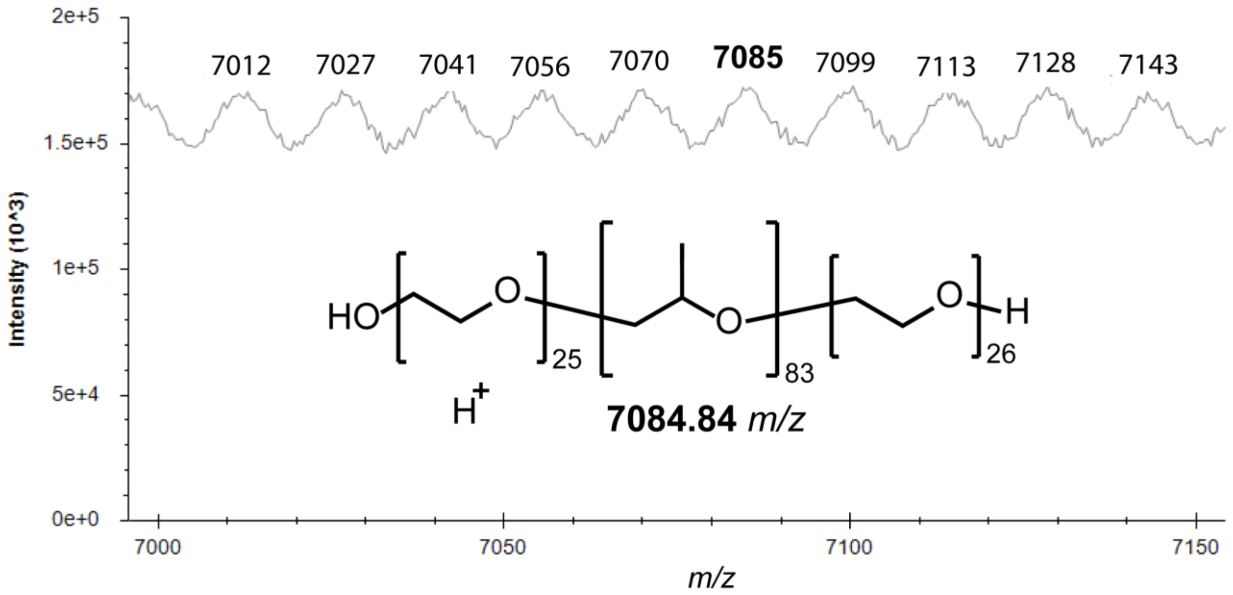

Figure S45 | MALDI Mass spectrum of **Pluronic** magnified to the *m/z* 7000 – 7150 range. Each peak corresponds to a Pluronic triblock copolymer of a different molecular weight. The *m/z* 14 or 15 spacings between peaks of are typical for pluronic triblock copolymers. The peak of highest intensity at *m/z* 7085 is assigned to the structural formula shown in the graph above its exact mass.

10. Transmission Electron Microscopy

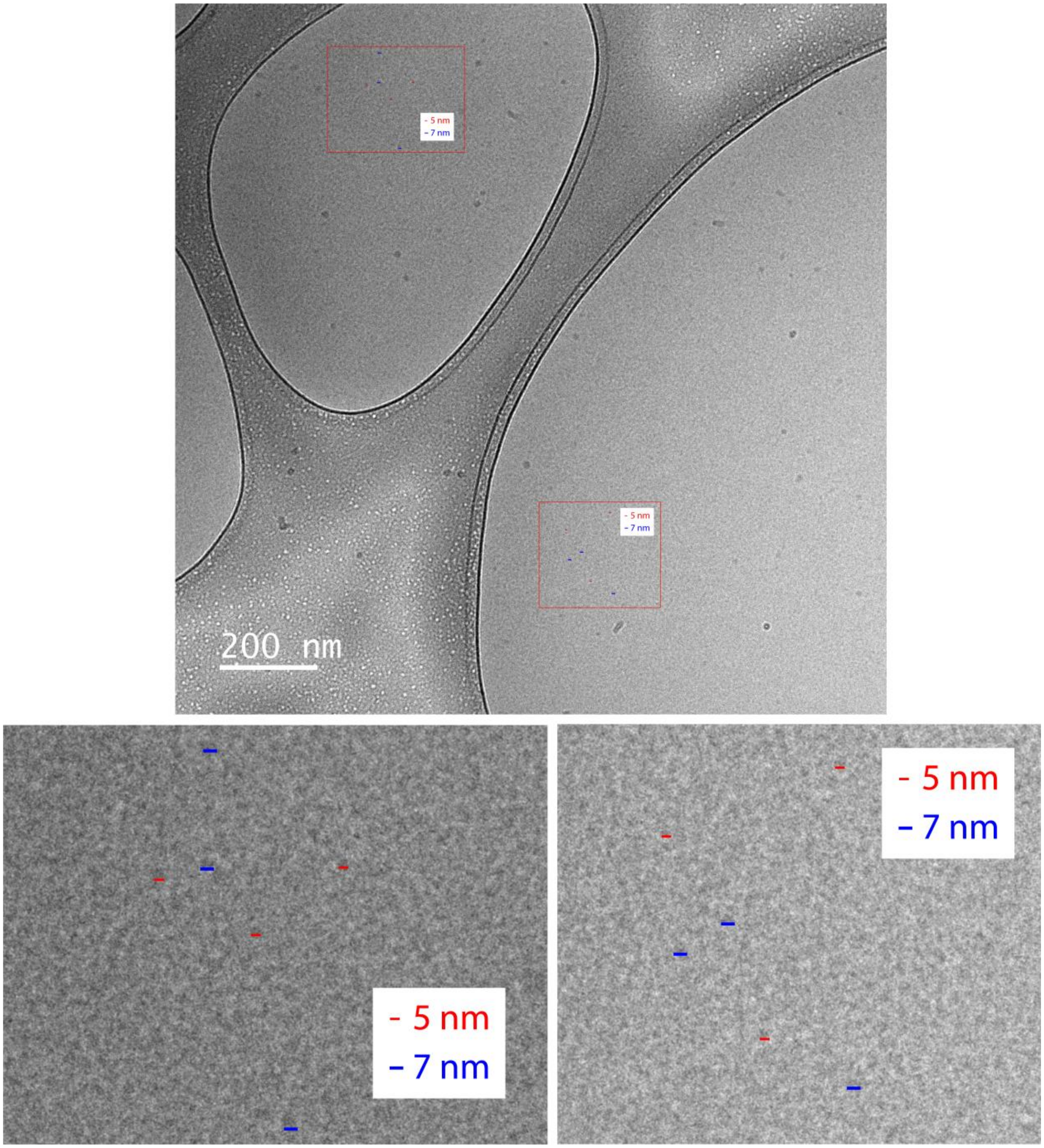

Figure S46 | Cryo-TEM image of the **Pre-Pump** micellar solution of **Pluronic-BP**•6TFA and **CBPQT**•4Cl taken at 30K magnification. (A) shows the entire image. (B) and (C) are magnifications of (A). The images show spherical or sphere-like micelles in the size range of 5 – 7 nm.

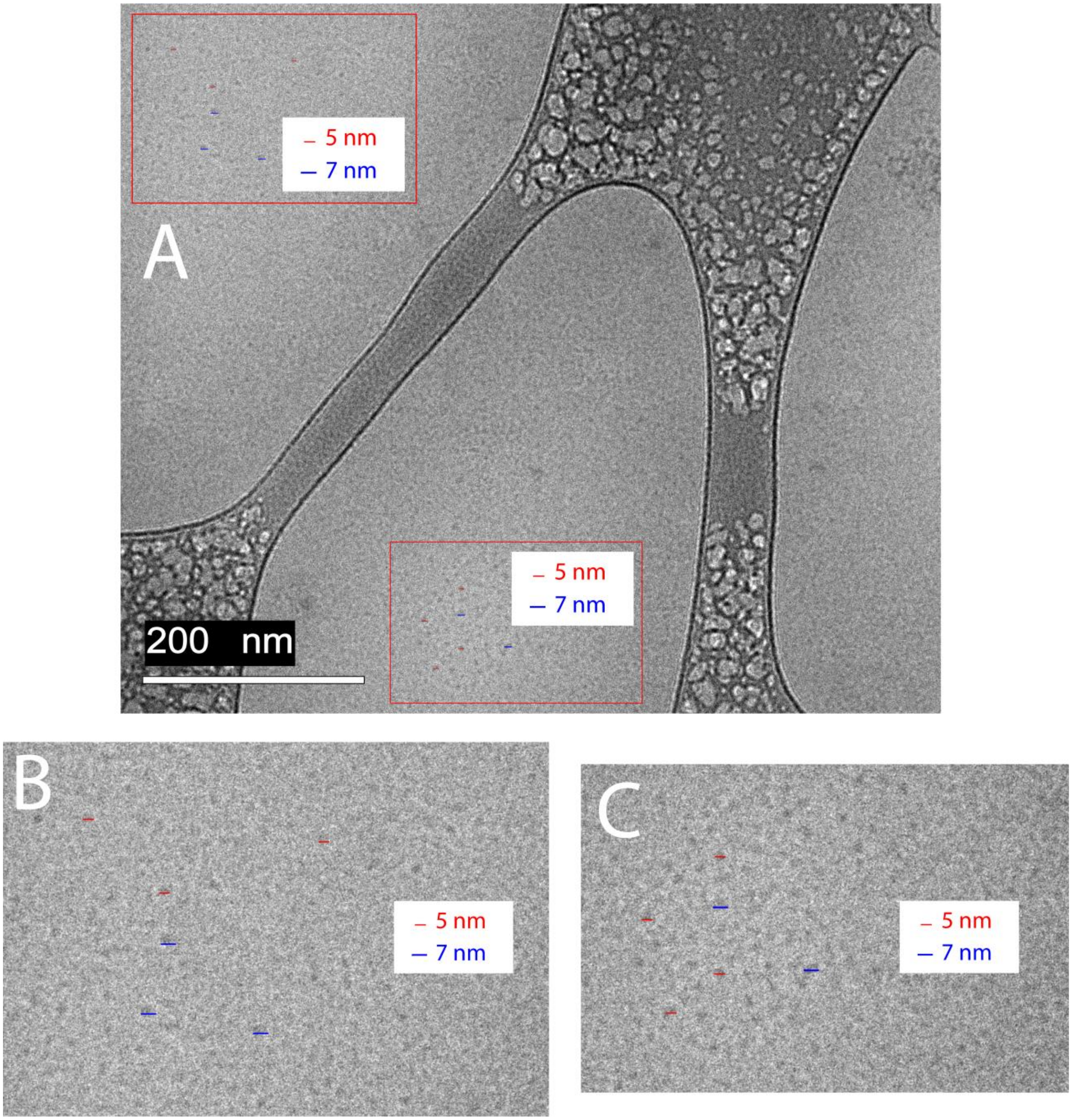

Figure S47 | Cryo-TEM image of the **Post-Pump** micellar solution taken at 60K magnification. (A) shows the entire image. (B) and (C) are magnifications of (A). The images show spherical or sphere-like micelles in the size range of 5 – 7 nm.

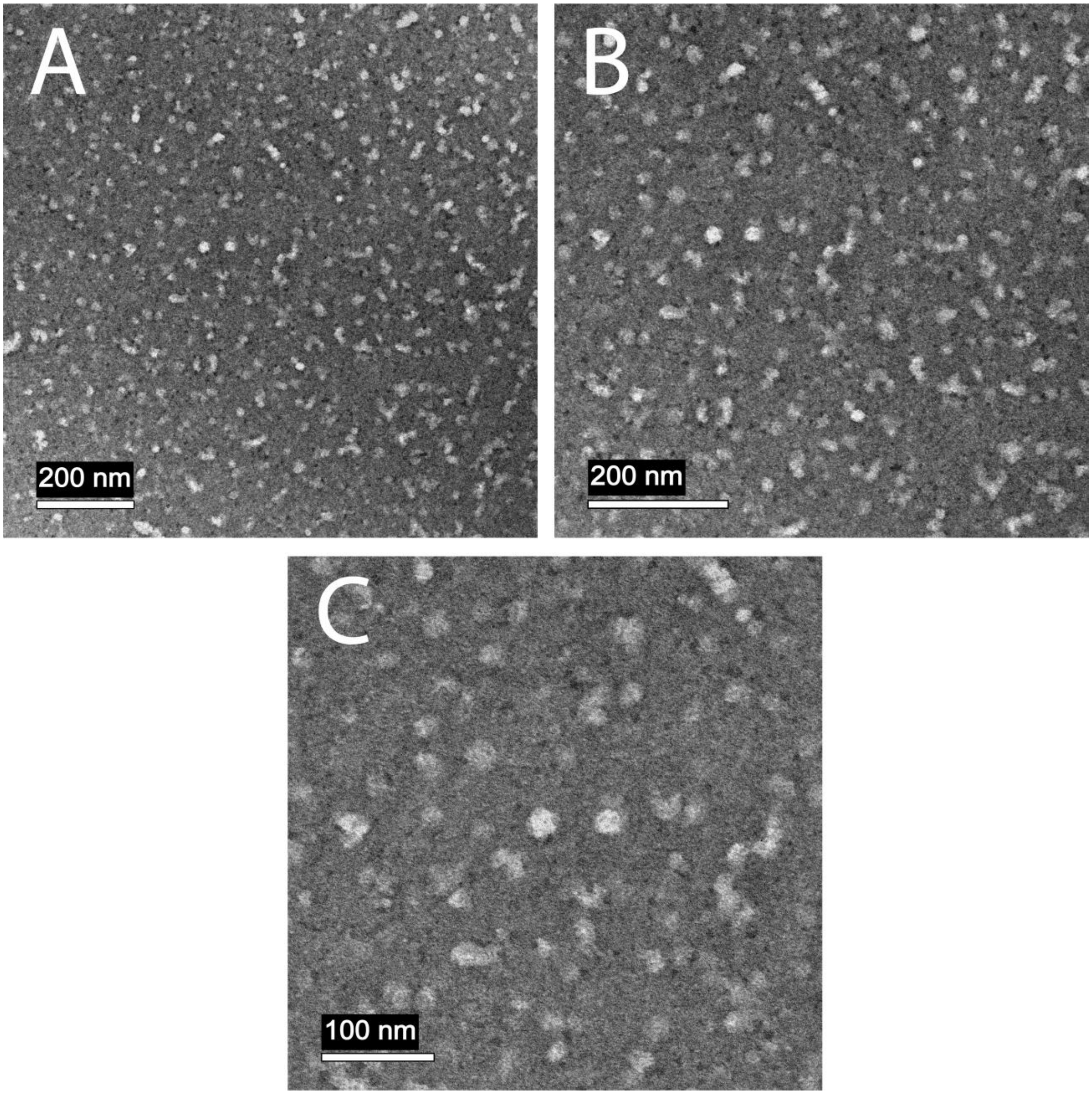

Figure S48 | TEM Images of the **Pre-Pump** micellar solution of **Pluronic-BP**•6TFA and **CBPQT**•4Cl taken at (A) 40K magnification, (B) 60K magnification and (C) 100K magnification. The images primarily show spherical particles in the size range of 10–15 nm. Wormlike features appear to be aggregates of the spherical particles.

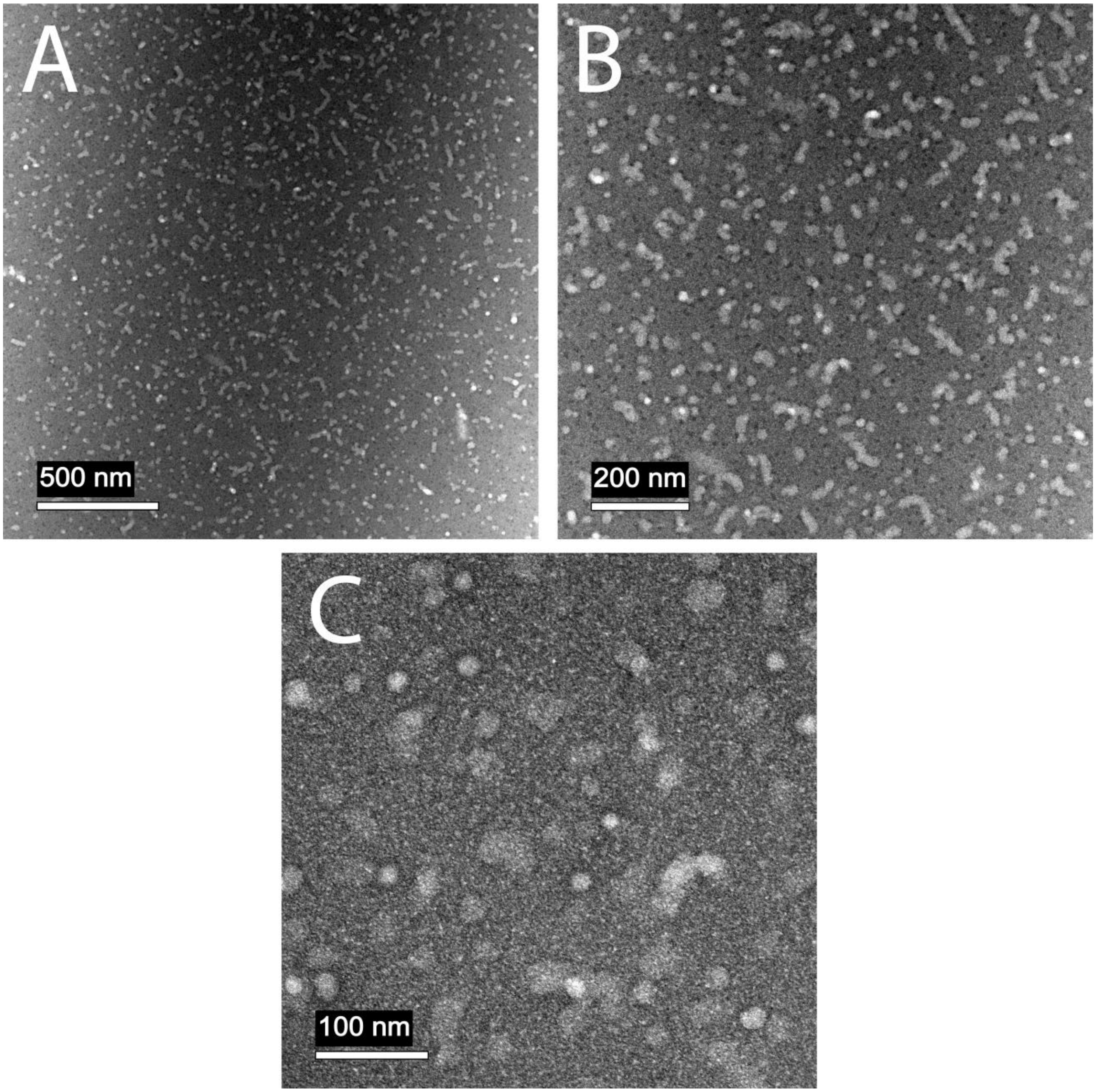

Figure S49 | TEM images of the **Post-Pump** micellar solution after oxidation using air flow. Images are shown at (A) 20K magnification, (B) 40K magnification and (C) 100K magnification. The images show spherical particles in the size range of 15–20 nm and wormlike features that may be chained aggregates of the spherical particles.

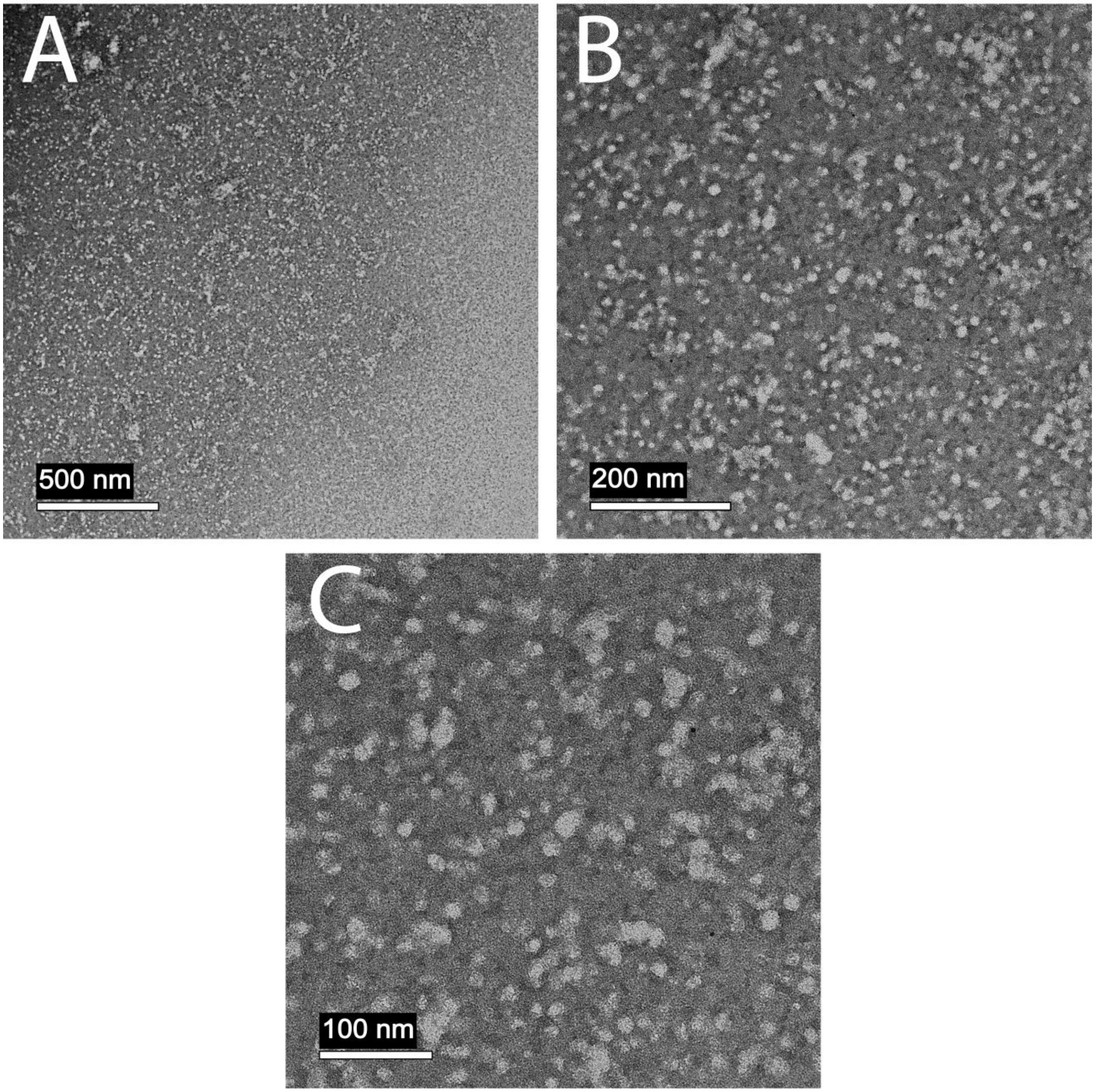

Figure S50 | TEM Images of the **Post-Pump** micellar solution after oxidation with elemental I_2. Images are shown at (A) 20K magnification, (B) 60K magnification and (C) 100K magnification. The images show spherical particles in the size range 10–20 nm and some wormlike features that may be aggregates of the spherical particles.

Discrepancy in micelle diameter measured by different techniques

The micellar diameters measured by Cryo-TEM are lower than those measured by ordinary TEM and by DLS. It has been noted previously[23] that it is typical for Cryo-TEM images to register low diameters for polymeric micelles, and we believe this discrepancy arises here as an artefact of the method, rather than from an actual discrepancy in micellar diameter.

11. Thermodynamic Analysis

The chemical potential difference between rings mechanically interlocked in micelles and rings in free solution ($\Delta\mu$) is given by the equation,

$$\Delta\mu = RTln(\frac{[r_{sol}]_{eq}}{[r_{mic}]_{eq}}\frac{[r_{mic}]_{ps}}{[r_{sol}]_{ps}}) \quad (1)$$

Where R is the gas constant, T is the temperature (approximately 298.15 K for all pumping experiments), and $[r]$ is the concentration of rings under given conditions. The subscripts *sol* and *mic* refer to rings in free solution and rings in micelles respectively, and the subscripts *eq* and *ps* refer to the equilibrium (pre-pump) state and the pumped state, respectively. The concentration $[r_{sol}]_{eq}$ is known from experiment, while the concentrations $[r_{mic}]_{eq}$, $[r_{mic}]_{ps}$ and $[r_{sol}]_{ps}$ were calculated from NMR spectroscopy data (calculation method shown below). Inserting these values we find $\Delta\mu$ > 17.3 kJ/mol.

The estimation of micelle aggregation number and volume

To calculate the concentration of **CBPQT**•4Cl rings within micelles, it was necessary to know the volume of the micelles (V_{mic}), which in turn required knowledge of the aggregation number of the micelles (N_{agg}). Three other studies of aqueous Pluronic micelles, performed at temperatures within the range 20 – 25 °C, found N_{agg} values for Pluronic micelles of: 92 [24], 86 [25], and 81 [26] (NB: the exponents are references). We used the rounded average value, N_{agg} = 86, for calculations.

Prior to pumping, the micelles have a mean diameter of 11.6 nm as indicated by the DLS results. The concentration of **Pluronic-BP**•6TFA chains is 0.55 mM, equating to 0.55 micromoles of chains per 1.0 cm^3. This converts to 3.3 x 10^{17} chains per 1 cm^3. Applying N_{agg} = 86, we find 3.9 x 10^{15} micelles per 1 cm^3 solution. Assuming the micelles to be, on average, spheres of diameter

11.6 nm, we calculate the volume of an average micelle to be 8.17 x 10^{-19} cm^3. Therefore, in 1 cm^3 of **Pre-Pump** solution, we obtain the micellar volume, $V_{mic,eq}$ = 3.1 x 10^{-3} cm^3. After pumping, the micelles have a mean diameter of 13.8 nm. We assume the concentration of **Pluronic-BP**•6TFA chains to have remained the same at 0.55 mM, equating to 0.55 micromoles of chains per 1 cm^3. This converts to 3.3 x 10^{17} chains per 1 cm^3. Applying N_{agg} = 86, we find 3.9 x 10^{15} micelles per 1 cm^3 solution. Assuming the **Post-Pump** micelles to be, on average, spheres of diameter 13.8 nm (as per the DLS results), we calculate the volume of an average micelle to be 1.38 x 10^{-18} cm^3. Therefore, in 1 cm^3 of **Post-Pump** solution, we obtain a micellar volume of $V_{mic,ps}$ = 5.4 x 10^{-3} cm^3.

The estimation of the concentration of CBPQT rings in micelles

NMR spectroscopy data was used to obtain an upper limit for the parameter $[r_{mic}]_{eq}$. The NMR resonance peaks for mechanically interlocked rings are distinct from those of rings in free solution, and in the **Pre-Pump** micellar solution, no resonance peaks corresponding to mechanically interlocked rings are visible (see Figure S17). We can therefore state that the number of mechanically interlocked rings in the **Pre-Pump** micellar solution (i.e., at equilibrium) is lower than the detection limit of our NMR method.

In the NMR spectrum of a **Pre-Pump** micellar solution, the signal-to-noise ratio (SNR) of the resonance peak at 7.66 ppm (corresponding to the phenylene protons of **CBPQT**$^{4+}$**)** was compared to baseline noise using Mestrenova's *SNR Calculation* tool, returning the average value SNR_{rings} = 11814 ± 1133. Let $C_{rings,det}$ be the minimum detectable concentration of mechanically interlocked **CBPQT**$^{4+}$ rings. To calculate this value for our NMR conditions, we apply the rule of thumb that a signal peak must be at least three times larger than the baseline noise to be defined as a detectable peak. We can write,

$$C_{rings,det} = C_{rings,free}\frac{3}{\mathrm{SNR}} \tag{2}$$

Where $C_{rings,free}$ is the concentration of rings in the **Pre-Pump** solution. This **Pre-Pump** solution used to calculate the SNR contained 1.6 mg/mL **CBPQT**•4Cl, which equates to a concentration of 2.41 mM. Inserting the values into Eq. 2, we find $C_{rings,min}$ = 0.612 μM. The value $C_{rings,min}$ represents the upper-limit concentration of mechanically interlocked rings in the *entire* NMR solution. In order to obtain $[r_{mic}]_{eq}$, the upper-limit concentration of rings within just the micelles, we multiply $C_{rings,min}$ by $\frac{V_{sol,eq}}{V_{mic,eq}}$. This returns the inequality $[r_{mic}]_{eq}$ < 0.195 mM. We expect that the actual value of $[r_{mic}]_{eq}$ may be significantly lower than this upper limit concentration.

The calculation of $[r_{mic}]_{ps}$ is simpler. From the synthetic pumping efficiency of 65% measured by NMR spectroscopy, we know that the concentration of mechanically interlocked rings in the solution is 65% of the concentration of molecular pumps in the solution: 0.715 mM. Multiplication of this concentration by $\frac{V_{sol,ps}}{V_{mic,ps}}$ gives $[r_{mic}]_{ps} = 132$ mM.

Note that here, $[r_{sol}]_{eq}$, and $[r_{sol}]_{ps}$, the concentrations of rings in free solution, have been taken to be equal to the concentration of rings in the entire NMR solution: the small adjustment to concentration arising from the excluded volume occupied by the micelles has been disregarded.

Energy Stored by the Pumping of Rings into Micelles

Active transport and the operation of energy ratchets have been evaluated in other research works in terms of *stored energy*[27], a figure of merit often[28,29] expressed in units of $J \cdot L^{-1}$. The chemical potential change of pumped rings calculated here (>17.3 kJ Mol^{-1}) can be converted into a value of stored energy (expressed in J L^{-1}) through the knowledge that the concentration of pumped rings in the **Post-Pump** solution is 0.715 mmol L^{-1}. This value is calculated based on the known

concentration of molecular pumps in the micellar solutions and the measured pumping efficiency. The calculation returns a stored energy of > 12.4 J L^{-1}.

The assumptions and limits in this quantitative treatment

The application of N_{agg} data from literature studies of P123 micelles to the micellar system used in this paper requires two assumptions: that neither (1) the use of slightly larger Pluronic chains (of composition PEG_{24}-PPG_{90}-PEG_{25} in this study as compared to PEG_{20}-PPG_{70}-PEG_{20} for standard Pluronic P123) nor (2) the end-capping of the polymers with molecular pumps greatly affects the N_{agg} of micelles. We have also assumed that the N_{agg} value remains the same after the pumping of rings into micelles. It is possible for **CBPQT**$^{4+}$ rings to exist in micelles in two states: as mechanically interlocked components of the polymer chains, or 'free' within the micelle. In our treatment, only the mechanically interlocked scenario is considered.

12. References

[1] I. B. Butler, M. A. A. Schoonen, D. T. Rickard, *Talanta* **1994**, *41*, 211–215.
[2] P. Groves, *Polym. Chem.* **2017**, *8*, 6700–6708.
[3] J. Horský, Z. Walterová, *Macromol. Symp.* **2014**, *339*, 9–16.
[4] C. Pezzato, M. T. Nguyen, C. Cheng, D. J. Kim, M. T. Otley, J. F. Stoddart, *Tetrahedron* **2017**, *73*, 4849–4857.
[5] J. C. Barnes, M. Juríček, N. A. Vermeulen, E. J. Dale, J. F. Stoddart, *J. Org. Chem.* **2013**, *78*, 11962–11969.
[6] D. S. Treitler, S. Leung, *J. Org. Chem.* **2022**, *87*, 11293–11295.
[7] N. Z. Fantoni, A. H. El-Sagheer, T. Brown, *Chem. Rev.* **2021**, *121*, 7122–7154.
[8] Y. Qiu, B. Song, C. Pezzato, D. Shen, W. Liu, L. Zhang, Y. Feng, Q.-H. Guo, K. Cai, W. Li, H. Chen, M. T. Nguyen, Y. Shi, C. Cheng, R. D. Astumian, X. Li, J. F. Stoddart, *Science* **2020**, *368*, 1247–1253.
[9] Y. Jiao, Y. Qiu, L. Zhang, W.-G. Liu, H. Mao, H. Chen, Y. Feng, K. Cai, D. Shen, B. Song, X.-Y. Chen, X. Li, X. Zhao, R. M. Young, C. L. Stern, M. R. Wasielewski, R. D. Astumian, W. A. Goddard, J. F. Stoddart, *Nature* **2022**, *603*, 265–270.
[10] H. Luftmann, G. Rabani, A. Kraft, *Macromolecules* **2003**, *36*, 6316–6324.
[11] I. R. Schmolka, *J. Biomed. Mater. Res.* **1972**, *6*, 571–582.
[12] A. Pitto-Barry, N. P. E. Barry, *Polym. Chem.* **2014**, *5*, 3291–3297.
[13] E. M. Kosower, J. L. Cotter, *J. Am. Chem. Soc.* **1964**, *86*, 5524–5527.
[14] P. M. S. Monk, N. M. Hodgkinson, S. A. Ramzan, *Dyes Pigments* **1999**, *43*, 207–217.
[15] M. R. Geraskina, A. S. Dutton, M. J. Juetten, S. A. Wood, A. H. Winter, *Angew. Chem. Int. Ed.* **2017**, *56*, 9435–9439.

[16] E. M. Kosower, J. Hajdu, *J. Am. Chem. Soc.* **1971**, *93*, 2534–2535.
[17] J. S. W. Seale, B. Song, Y. Qiu, J. F. Stoddart, *J. Am. Chem. Soc.* **2022**, *144*, 16898–16904.
[18] D. Sinnaeve, *Concepts Magn. Reson. Part A* **2012**, *40A*, 39–65.
[19] F. J. Millero, Roger. Dexter, Edward. Hoff, *J. Chem. Eng. Data* **1971**, *16*, 85–87.
[20] H. Li, A. C. Fahrenbach, A. Coskun, Z. Zhu, G. Barin, Y.-L. Zhao, Y. Y. Botros, J.-P. Sauvage, J. F. Stoddart, *Angew. Chem. Int. Ed.* **2011**, *50*, 6782–6788.
[21] A. Trabolsi, N. Khashab, A. C. Fahrenbach, D. C. Friedman, M. T. Colvin, K. K. Cotí, D. Benítez, E. Tkatchouk, J.-C. Olsen, M. E. Belowich, R. Carmielli, H. A. Khatib, W. A. Goddard, M. R. Wasielewski, J. F. Stoddart, *Nat. Chem.* **2010**, *2*, 42–49.
[22] Y. Wang, M. Frasconi, W.-G. Liu, Z. Liu, A. A. Sarjeant, M. S. Nassar, Y. Y. Botros, W. A. I. Goddard, J. F. Stoddart, *J. Am. Chem. Soc.* **2015**, *137*, 876–885.
[23] K. Mortensen, Y. Talmon, *Macromolecules* **1995**, *28*, 8829–8834.
[24] J. Bhattacharjee, G. Verma, V. K. Aswal, V. Patravale, P. A. Hassan, *RSC Adv.* **2013**, *3*, 23080–23089.
[25] G. Wanka, H. Hoffmann, W. Ulbricht, *Macromolecules* **1994**, *27*, 4145–4159.
[26] S. Manet, A. Lecchi, M. Impéror-Clerc, V. Zholobenko, D. Durand, C. L. P. Oliveira, J. S. Pedersen, I. Grillo, F. Meneau, C. Rochas, *J. Phys. Chem. B* **2011**, *115*, 11318–11329.
[27] L. Binks, S. Borsley, T. R. Gingrich, D. A. Leigh, E. Penocchio, B. M. W. Roberts, *Chem* **2023**, *9*, 2902–2917.
[28] T. Marchetti, D. Frezzato, L. Gabrielli, L. J. Prins, *Angew. Chem. Int. Ed.* **2023**, *62*, e202307530.
[29] K. Liang, F. Nicoli, S. A. Shehimy, E. Penocchio, S. Di Noja, Y. Li, C. Bonfio, S. Borsley, G. Ragazzon, *Angew. Chem. Int. Ed.* **2025**, *64*, e202421234.
